# Post‐Synthetic Zinc Modifications to Correct Speciation in Ti‐MWW Zeolites for Catalytic Oxidation

**DOI:** 10.1002/anie.2516685

**Published:** 2026-08-23

**Authors:** Christoph J. Kaul, Lukas Lätsch, Alexander V. Yakimov, Milivoj Plodinec, Xing Huang, Trees De Baerdemaeker, Jaroslaw Mormul, Peter Oechsle, Andrei‐Nicolae Parvulescu, J. Henrique Teles, Christophe Copéret

**Affiliations:** ^1^ Department of Chemistry and Applied Biosciences ETH Zurich Zurich Switzerland; ^2^ Scientific Center for Optical and Electron Microscopy (ScopeM) ETH Zurich Zurich Switzerland; ^3^ BASF SE Ludwigshafen Germany

**Keywords:** DFT modelling, NMR spectroscopy, oxidation catalysis, x‐ray absorption, zeotype catalysts

## Abstract

Ti‐MWW is an important member of Ti‐containing zeolites, industrially employed as heterogeneous catalyst for selective oxidations. However, the development of more active variants is often hampered by the lack of characterization methods able to track transformations and interplay between a multitude of species; this is further exacerbated by the use of post‐synthetic modifications. Combining microscopy and various spectroscopic techniques show that the promotional role of Zn is closely linked to its healing of hydrophilic silanol nests, while simultaneously generating small TiO_x_ clusters. Notably, these changes are accompanied by Ti weight loading dependent modifications of the extra‐framework TiO_2_ and formation of Zn species. Overall, our study not only highlights the interplay between a plethora of species, but enables a more rational development of post‐modification of Ti‐containing zeolites.

## Graphical abstract

Post‐synthetic Zn modifications of Ti‐MWW zeolite catalysts exhibit improved catalytic properties in the selective oxidation reaction, namely the ammoximation reaction. The origin of the beneficial effect of Zn is related via an array of spectroscopic and microscopic techniques to the modification of extra‐framework TiO2 and TiOx clusters and the engineering of silanol defect sites, leading toward faster formation and more stable of Ti μ_2_‐η^2^:η^2^‐peroxo species.

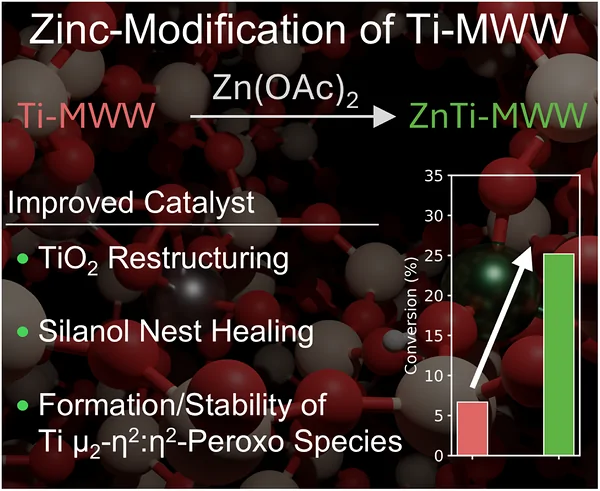

## 1 Introduction

Ti‐zeotypes are an important class of heterogeneous catalysts, where titanium is incorporated into a crystalline microporous SiO_2_ matrix [1, 2]. They enable selective oxidation reactions using hydrogen peroxide as a primary oxidant; the most prominent examples are the epoxidation of propylene (HPPO process) and the ammoximation of cyclohexanone or butanone, often referred to in industry as methyl ethyl ketone (MEK) (see Scheme 1a) [3, 4, 5]. These processes run on a megaton scale since propylene oxide and cyclohexanone oxime are key building blocks for the polymer industry, for polyether polyols and nylon‐6, respectively [6, 7], while methyl ethyl ketoxime (MEKO) is widely used as an antiskinning agent in paints, for surface coatings and in adhesives [8]. Similarly to the HPPO process, the ammoximation of ketones has also experienced tremendous growth rates in recent years (with >5% annual growth rate forecasted until 2030), yielding multi‐billion industries [9, 10, 11].

**SCHEME 1:**
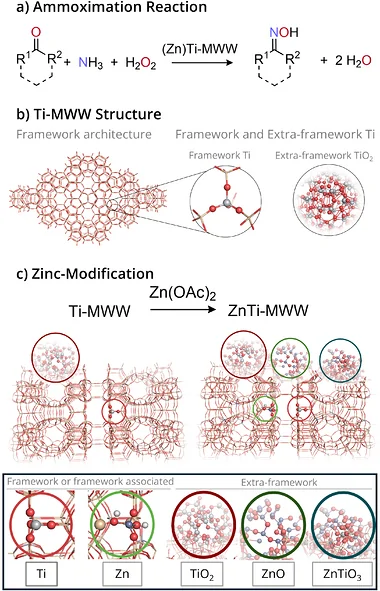
(a) Industrial ammoximation of ketones. (b) Ti‐MWW structure: framework architecture and Ti speciation (framework and extra‐framework). (c) Schematic depiction of the post‐synthetic zinc modification.

Notably, while the epoxidation of propylene and the ammoximation of cyclohexanone rely on titanium silicalite‐1 (TS‐1) as the preferred catalyst, Ti‐MWW displays superior catalytic performances for the ammoximation of MEK [12, 13, 14]. Even though MEK and MEKO are small enough to enter the pores of TS‐1, their slow diffusion increases the residence time, ending in considerable overoxidation to ultimately yield 2‐nitrobutane. Ti‐MWW outperforms TS‐1 because of its enhanced pore accessibility, but it has required catalyst optimization to increase catalyst lifetime (limited to 200 h) and to minimize regeneration cycles [15]. Recent studies have also shown that Ti‐MOR (with even larger pores and the Ti located in defective T3 positions) enables efficient ammoximation [14, 16, 17]. Controlling the preferential T‐siting in Ti‐MWW is however more complicated due to the specific complexity of the MWW framework (see Scheme 1b) [18]. Thus alternative strategies have been explored to stabilize Ti‐MWW, such as the introduction of a secondary metal, most notably zinc. Zn‐modification has been achieved for both TS‐1 and Ti‐MWW through impregnation of the Ti‐zeotypes with zinc‐acetate solutions [19, 20].

For Ti‐zeotypes, H_2_O_2_ efficiency in the epoxidation of 1‐hexene has been reported to increase from ca. 75% to 95% upon zinc treatment, regardless of Ti weight loading, while maintaining high activity [20]. The origin of superior catalytic performance in epoxidation reactions has been ascribed to a combination of factors, including the neutralization of acid sites with Zn species, the incorporation of zinc into the framework yielding locally more relaxed and reactive species [19, 20]. Incorporated Zn adjacent to Ti has also been proposed to alter beneficially the electropositivity of Ti [19, 20]. These hypotheses originate from insights obtained from UV–vis diffuse reflectance spectroscopy (DRS) and powder x‐ray diffraction (pXRD), that show an additional absorption band at 228 nm, while no formation of ZnO is evidenced via pXRD [19]. In addition, it was proposed that the introduction of zinc also restrains the formation of extra‐framework anatase TiO_2_ as monitored by UV–vis DRS and Fourier transform infrared (FT‐IR) spectroscopy [20]. Overall, while the post‐synthetic introduction of zinc into Ti‐MWW increases catalytic performance, the molecular origin of this promotional effect has so far largely remained unclear.

Therefore, access to meaningful spectroscopic descriptors for the catalytic activity in ammoximation reaction catalyzed by Ti‐zeotypes are needed, highlighting the necessity for detailed and expanded characterization approaches with the goal to track the presence and/or interconversion of a multitude of species, building on recent findings from our group to assess Ti sites in TS‐1 catalysts [21, 22, 23, 24, 25]. Most notable, ^17^O solid‐state nuclear magnetic resonance (NMR) spectroscopy has been established to track dinuclear peroxo species in TS‐1 catalyst, formed as a resting state analogously to homogeneous Ti‐based epoxidation catalysts [21, 26]. Moreover the formation and stability of the Ti µ_2_‐η^2^:η^2^‐peroxo and the Ti accessibility probed by ^15^N pyridine has been found to relate to catalytic activity [22, 24]. Additionally, the use of soft x‐rays at the Ti L_2,3_‐edge has enabled to selectively probe Ti sites close to the catalyst surface [23].

In this study, we expand these methods to employ the µ_2_‐η^2^:η^2^‐peroxo formation and stability as well as the Ti site accessibility and speciation as descriptors in another selective oxidation reaction with Ti‐zeotypes, namely the ammoximation reaction. These efforts combined an array of advanced spectroscopic characterization methods, including ^17^O and ^15^N solid‐state NMR, soft x‐rays (at the Ti L_2,3_‐edge), and hard x‐rays (at the Ti K‐ and Zn K‐edges), in combination with microscopy, FT‐IR, and diffuse reflectance (DR) UV–vis spectroscopy enable to elucidate the Ti‐ and Zn‐speciation on the molecular level (see Scheme 1c).

## 2 Results

### 2.1 Catalytic Performance of (Zn)Ti‐MWW Samples

For this study, we select four (Zn)Ti‐MWW samples:

- Ti‐MWW L (1.8 wt% Ti),
- ZnTi‐MWW L (1.8 wt% Ti, 1.3 wt% Zn),
- Ti‐MWW H (2.8 wt% Ti) and,
- ZnTi‐MWW H (2.4 wt% Ti, 1.4 wt% Zn) [27, 28].

For simplicity, the (H)igh weight Ti loading samples (>2 wt%) are referred to as (Zn)Ti‐MWW H in this work, and the (L)ow weight Ti loading (<2 wt%) samples as (Zn)Ti‐MWW L. Ti‐MWW L and ZnTi‐MWW L, as well as Ti‐MWW H and ZnTi‐MWW H represent pairs, where the parent Ti‐MWWs have been impregnated with an aqueous zinc acetate (Zn(OAc)_2_) solution, as previously described (see Scheme 1c) [27, 28]. The decrease in Ti weight loading for the ZnTi‐MWW H compared its parent Ti‐MWW sample (see Table 1) is ascribed to leaching of a small amount of Ti during the post‐synthetic modification. A summary of the synthesis and characterization via pXRD can be found in Sections SA and SB.1. The zeotypes are chosen to investigate the effect of the zinc treatment on samples where Ti is fully incorporated into the framework ((Zn)Ti‐MWW L), and where TiO_x_‐domains are present ((Zn)Ti‐MWW H).

**TABLE 1:** Summary of Ti and Zn weight loadings and the catalytic performance data of the (Zn)Ti‐MWW library studied in this work. *k*_80_ refers to the pseudo‐first order decomposition rate constant of H_2_O_2_. The ammoximation reaction of acetone is used as a representative model reaction.

Samples	Ti (wt%)	Zn (wt%)	*k*_80_ (h^−1^)^a^	Acetone conversion in the ammoximation reaction (%)^b^
**Ti‐MWW L**	1.8	—	0.66	6.7
**ZnTi‐MWW L**	1.8	1.3	0.28	25.2
**Ti‐MWW H**	2.8	—	0.14	2.9
**ZnTi‐MWW H**	2.4	1.4	0.40	23.3

^a^ The error of *k*_80_ is in the order of 0.05 h^−1^.

^b^ The ammoximation conversion was determined by running duplicates and the maximum deviation from the mean is 1.5%.

To compare catalytic performances across the four samples and to develop a structure‐activity relationship, the ammoximation of acetone is selected as a representative model reaction (for experimental details see Section SC.2). While all samples are catalytically active, the conversion of acetone changes by up to one order of magnitude for the ZnTi‐MWW samples—in comparison to their parent Ti‐MWW samples (6.7% → 25.2% ammoximation conversion for (Zn)Ti‐MWW L and 2.9% → 23.3% ammoximation for (Zn)Ti‐MWW H, see Table 1). The improved performance and Zn‐promotional effect are in line with previous reports for other selective oxidation reactions (epoxidation of 1‐hexene) catalyzed with zinc treated Ti‐MWW samples [20].

In addition to the observed enhanced catalytic performances, the decomposition rate constant (*k*_80_) of hydrogen peroxide (standardized test, pseudo‐first‐order, performed at 80°C, as reported earlier [22], see Section SC.1 for further details) is reduced for the low weight loading Zn treated sample (ZnTi‐MWW L) compared to its parent Ti‐MWW sample from 0.66 h^−1^ to 0.28 h^−1^, hence increasing the overall selectivity. In sharp contrast, the beneficial effect of Zn on H_2_O_2_ decomposition is reversed for the high weight loading sample, changing from a very minimal decomposition rate constant of 0.14 h^−1^ for the parent Ti‐MWW H to 0.40 h^−1^ for the ZnTi‐MWW H.

### 2.2 Peroxo Formation and Stability via ^17^O NMR Quantification

With these results in hand, we next focus on understanding the molecular origin of the Zn‐promotional effect and deconvolute the different influences toward the decomposition rate (focusing on peroxo formation and stability).

We have previously shown that it is possible to relate the formation and stability of µ_2_‐η^2^:η^2^‐peroxo species formed on Ti‐zeotypes to catalytic performances (for the decomposition reaction of hydrogen peroxide, relating to the overall selectivity of the catalyst). Using the same approach (for details see Section SD.1 and the original publication) [22], we thus examine the impact of the zinc treatment on both the formation and stability of Ti‐peroxo species.

Focusing on the high Ti weight loading (Zn)Ti‐MWW H samples, a clear difference in the ^17^O NMR signatures after 15 min of impregnation with 1 equiv. of H_2_^17^O_2_ per Ti is observed between the zinc treated sample and the parent Ti‐MWW (see Figure 1a). Besides, a signature assigned to stable Ti‐peroxo, Ti‐MWW H displays additional features attributed to unconsumed H_2_^17^O_2_ and H_2_^17^O/Si‐^17^O‐Si (arising from decomposition of the peroxo to H_2_^17^O and subsequent framework incorporation). In contrast the zinc treated sample ZnTi‐MWW H displays significantly larger fraction of the stable Ti‐peroxo sites. The clear change upon zinc treatment in the distribution of the species motivates us to investigate also a Ti‐free ZnSi‐MWW sample after impregnation with H_2_^17^O_2_. Notable for ZnSi‐MWW the obtained ^17^O NMR spectrum after 15 min displays mainly unconsumed H_2_^17^O_2_, accompanied with decomposition products (see Figure 1a), indicating that no stable peroxo is formed without Ti, in agreement with its inactivity in the ammoximation reaction (0.9% acetone conversion). Yet, the decomposition rate of H_2_O_2_ on the Zn sites is within error of the baseline, as evidenced by the low *k*_80_ value of 0.03 h^−1^.

**FIGURE 1:**
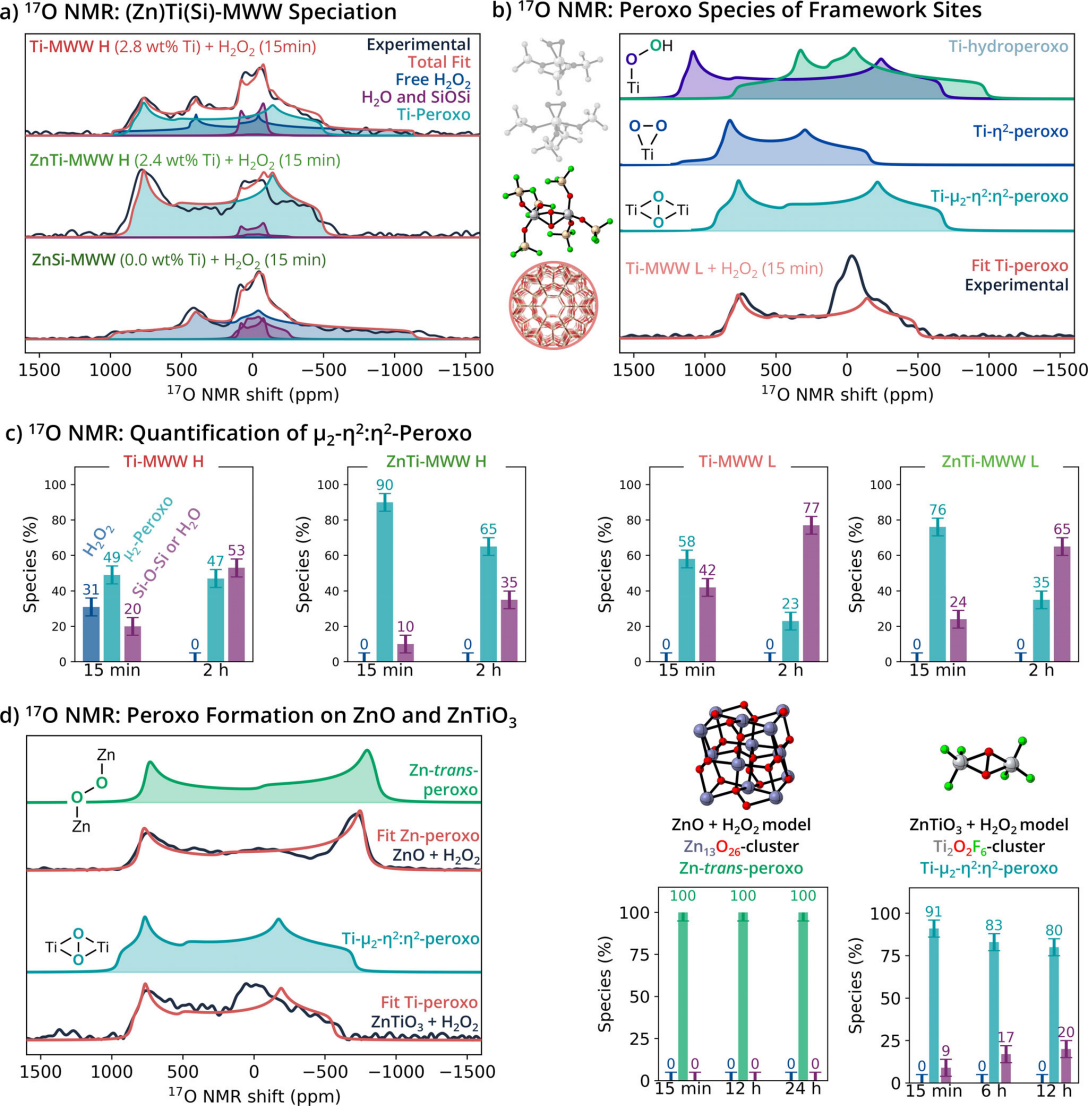
(a) Deconvolution of ^17^O NMR spectra (WCMPG, 14.1 T, 100 K, static, referenced to H_2_O at 0 ppm at room temperature) of (Zn)Ti‐MWW H and ZnSi‐MWW impregnated with aqueous H_2_^17^O_2_. (b) Assignment of the peroxo species formed with framework Ti sites as evidenced from ^17^O NMR and DFT computations. (c) Evaluation of peroxo formation and stability for Ti‐MWW H,L and ZnTi‐MWW H,L via ^17^O NMR (samples impregnated with aqueous H_2_^17^O_2_, 1 equiv. per Ti, species quantification plotted with estimated error bars). (d) ^17^O NMR of ZnTiO_3_ and ZnO impregnated with aqueous H_2_^17^O_2_ (4 H_2_^17^O_2_/nm^2^) to assess peroxo formation and stability. The assignment to a *trans*‐peroxo on ZnO vs. a µ_2_‐η^2^:η^2^‐peroxo on ZnTiO_3_, is in line with simulated NMR lineshapes based on DFT calculated parameters.

In the next step the ^17^O NMR spectroscopic feature of the Ti‐peroxo species is investigated, where the Ti‐MWW L (extra‐framework TiO_2_‐free sample, vide infra) is selected as prototypical Ti‐MWW sample. Similar to TS‐1 [21], the ^17^O NMR spectroscopic signature observed can be best described by a bridging µ_2_‐η^2^:η^2^‐peroxo, because η^2^‐ and hydroperoxos are associated with distinct ^17^O NMR signatures according to density functional theory (DFT) calculations (see Figure 1b, for computational details see Section SG.1).

When comparing the relative amount of unconsumed H_2_^17^O_2_, the µ_2_‐η^2^:η^2^‐peroxo and H_2_^17^O/Si‐^17^O‐Si, significant differences can be noted across (Zn)Ti‐MWW H and L (see Figure 1a,c). Ti‐MWW L shows fast formation of the µ_2_‐η^2^:η^2^‐peroxo (full consumption of 1 equiv. of H_2_^17^O_2_ per Ti within 15 min after impregnation), whereas for Ti‐MWW H ca. 31% of H_2_^17^O_2_ remain, that are only consumed within 2 h. Notably, upon zinc treatment, the formation of the µ_2_‐η^2^:η^2^‐peroxo is accelerated for both ZnTi‐MWW H and L compared to the parent Ti‐MWW H and L catalyst, with a drastic change for ZnTi‐MWW H (49% → 90% µ_2_‐η^2^:η^2^‐peroxo, see Figure 1a,c). In terms of stability, both samples display a higher lifetime of the µ_2_‐η^2^:η^2^‐peroxo upon zinc modification (47% → 65% µ_2_‐η^2^:η^2^‐peroxo remaining for (Zn)Ti‐MWW H and 23% → 35% µ_2_‐η^2^:η^2^‐peroxo remaining for (Zn)Ti‐MWW L, see Figure 1c and Section SD.2).

Notably, ZnTi‐MWW H displays very high peroxo stability, even after 2 h. To further support these findings, we next assess the long‐term stability of this species via Ti K‐edge x‐ray absorption near edge structure (XANES). Ti K‐edge XANES offers the advantage that the same sample can be measured in a continuous fashion, whereas ^17^O NMR samples have to be prepared for every time‐step. In addition, for samples with very low peroxo content, NMR measurement times become rather long due to the low signal‐to‐noise level associated with broad signals. In XANES, the stability of the peroxo species can be monitored via the pre‐edge intensity, e.g. evaluating the time after impregnation with aqueous H_2_O_2_ until the pre‐edge intensity returns to the level measured for the same sample under ambient conditions (hydrated sample, for details see Section SB.5.3) [24]. For Ti‐MWW H, the time until full decomposition of 1 equiv. H_2_O_2_ per Ti is ca. 8 h. In contrast, for ZnTi‐MWW H, the time reaches ca. 16 h (Figure S11). Thus, the zinc treatment leads to an increase in stability of the peroxo species by a factor of ca. two. Furthermore, these findings corroborate the stability deducted from ^17^O NMR spectroscopy. Notably, the pre‐edge intensity for the hydrated sample (both initial and after peroxo decomposition) is significantly lower than reported for TS‐1 (about 50%), implying the formation of higher coordinated Ti species than in TS‐1 [24]. Noteworthy, such a difference is not observed when comparing pyridine adsorbed TS‐1 and Ti‐MWW, suggesting that Ti‐MWW allows for higher coordination upon contact with water, whereas the Ti sites do not allow for the adsorption of two pyridine molecules due to steric hinderance (Figure S10).

Next, we try to disentangle the effects of the potential Zn involvement in the peroxo formation via additional ^17^O NMR experiments. As shown above, according to ^17^O NMR and the H_2_O_2_ decomposition rate, the majority of Zn sites (Zn sites anchored to the framework, vide infra) in Ti‐free ZnSi‐MWW do not form stable peroxo sites and only marginally decompose H_2_O_2_. However, since additional extra‐framework Zn sites are proposed to be formed (infra vide), we independently investigate the peroxo formed on ZnO and ZnTiO_3_, respectively. Notably, in contrast to ZnSi‐MWW, well‐defined peroxo species are formed on both ZnTiO_3_ and ZnO within 15 min of impregnation (see Figure 1d, full coverage of 4 H_2_^17^O_2_/nm^2^). Note however that their spectroscopic signatures are clearly distinct, hinting toward the formation of different peroxo species, for example with and without involvement of Zn in the first coordination sphere. In terms of stability, both peroxo species display remarkably high stability — with ZnO decomposing almost no H_2_O_2_, and ZnTiO_3_ only ca. 20%, even after 24 h. However, we observe two different peroxo species. The peroxo species formed on ZnTiO_3_ have similar ^17^O NMR parameters as the bridging µ_2_‐η^2^:η^2^‐peroxo species observed for Ti‐MWW, hinting toward a similar µ_2_‐η^2^:η^2^‐peroxo configuration on the ZnTiO_3_ surface; this hypothesis is further supported by a computational model system (see Figure 1d), which is constructed to reflect the Ti‐O‐Ti sites in ZnTiO_3_ but terminated by fluoride anions (Ti_2_O_2_F_6_‐cluster). The preferential binding of H_2_O_2_ to Ti over Zn can also be inferred from the low binding affinity of H_2_O_2_ to Zn [29], even though mixed metal‐zinc peroxides have been reported [30]. In contrast, the peroxo formed on ZnO exhibits significantly different ^17^O NMR parameters, in line with the formation of a *trans*‐peroxo species. In fact, the structure of ZnO_2_ (zinc peroxide) displays such a structural motif (Zn‐O‐O‐Zn) [31], showcasing that ^17^O NMR spectroscopy can — not only — distinguish between hydroperoxos and the type of peroxo (terminal vs. bridging) [21], the remaining coordination sphere of the µ_2_‐η^2^:η^2^‐peroxo (µ_2_‐η^2^:η^2^‐peroxo on framework sites vs. on TiO_2_) [22], but also between different types of bridging peroxos (µ_2_‐η^2^:η^2^‐ vs. *trans*‐peroxo, as reported here). Our ^17^O NMR study also shows that both the peroxo formed on ZnTiO_3_ and ZnO are very stable, suggesting that these species are likely spectator species during catalysis. We have also measured the ZnTiO_3_ catalyzed decomposition rate constant of H_2_O_2_, that shows a similar value (*k*_80_ = 0.45 h^−1^) than for anatase nanoparticles (*k*_80_ = 0.38 h^−1^) [22] both being an order of magnitude lower than in the case of rutile nanoparticles (*k*_80_ = 3.21 h^−1^) [22]. Notably, zinc peroxide is even commercially used in dusting powders and as a deodorant [32]. Therefore we conclude, that Zn treatment leads to Zn species only marginally decomposing H_2_O_2_, accompanied with Zn species forming stable spectator peroxo species.

Overall, the analysis via ^17^O NMR supported by Ti K‐edge XANES clearly shows for both ZnTi‐MWW H and L, a faster (evidenced via ^17^O NMR) and more stable generation of the Ti‐peroxo species (evidenced via ^17^O NMR and Ti K‐edge XANES, the origin of the increased stability and its effect on the catalytic activity will be discussed in the next sections).

### 2.3 Zn Speciation and Defect Silanol Nest Healing

Intrigued by the Zn promoted catalytic activity and its effect on the formation and stability of the Ti µ_2_‐η^2^:η^2^‐peroxo species, we set out to directly probe the Zn speciation via Zn K‐edge XANES (see Section SB.7 for details) [33]. Previous analyses of Zn containing materials have shown that the overall Zn K‐edge XANES spectroscopic signature can be dominated by hydrated Zn species with high coordination spheres (≥ 6), which lead to high white line intensities [34, 35].

Therefore, we next study the dehydrated materials (see Figure 2a), yielding for both ZnTi‐MWW H,L samples Zn K‐edge XANES signatures which are reminiscent of Zn single sites (Zn@SiO_2_ was synthesized according to literature procedure [36]). Moreover, the white line features of both ZnTi‐MWW H,L are increased compared to the dispersed Zn sites, attributed to the presence of extra‐framework Zn in the form of ZnO or ZnTiO_3_ (vide infra). Based on the finding that ZnTi‐MWW L shows a slightly higher white line intensity compared to ZnTi‐MWW H (see Figure 2a), we rationalize that a marginally larger fraction of these extra‐framework sites is present in ZnTi‐MWW L.

**FIGURE 2:**
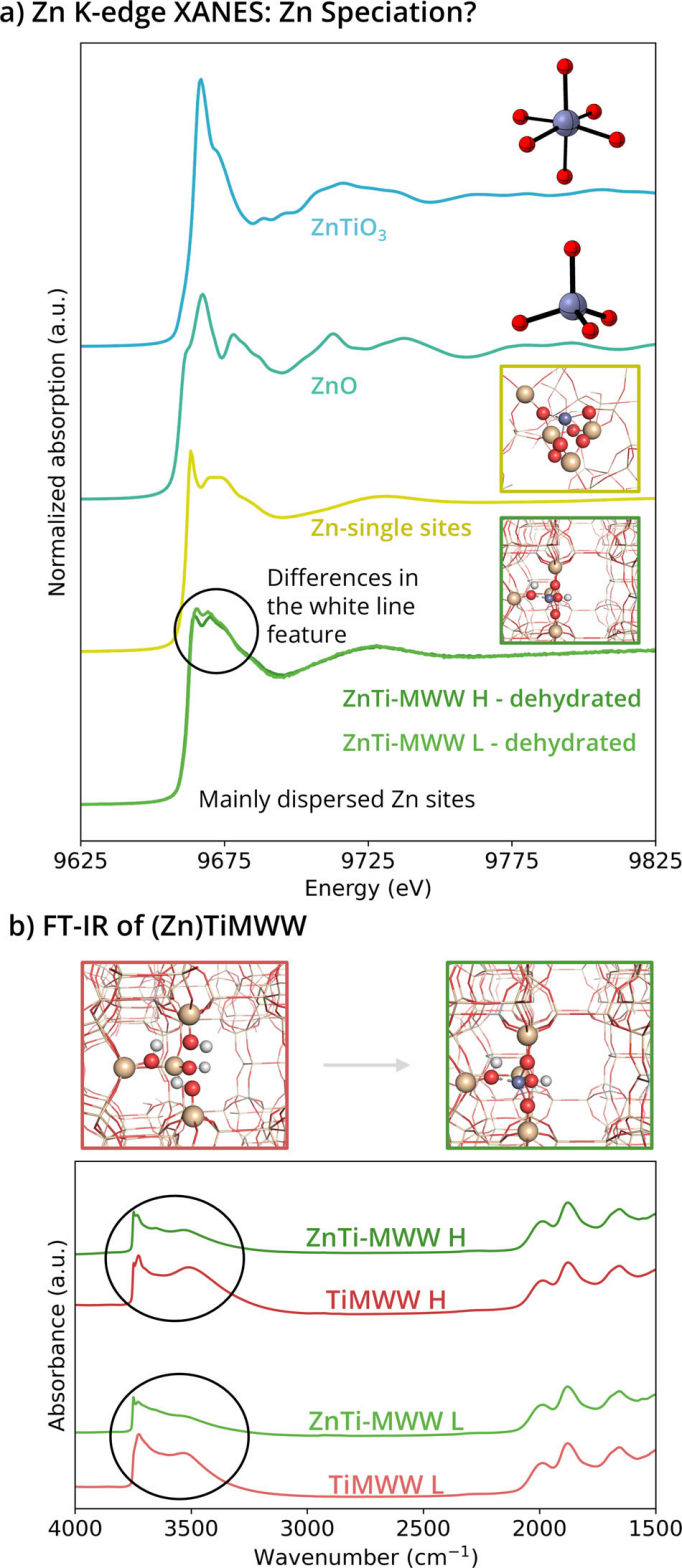
(a) Zn K‐edge XANES of dehydrated ZnTi‐MWW H and ZnTi‐MWW L in comparison with Zn references (Zn‐single sites, ZnO, and ZnTiO_3_). (b) FT‐IR spectra of dehydrated (Zn)Ti‐MWW H,L (absorbance normalized to siloxane bridges at 1900 cm^−1^) and the proposed healing mechanism of silanol nests by zinc.

Next focusing on the possible Zn incorporation mechanism, we turn to FT‐IR spectroscopy of dehydrated samples. Thereby we find that the IR stretches in the silanol region (3200 — 3800 cm^−1^) sites are highly dependent on whether the parent Ti‐MWW or Zn treated samples are investigated (see Figure 2b). Ti‐MWW samples display significant amounts of external and hydrogen‐bounded silanols, whereby the latter are attributed to the presence of silanol nests. Upon Zn treatment, these strongly interacting silanols are significantly decreased, in agreement with previous reports of zinc treated TS‐1 catalysts [20], presumably due to the exchange of Si‐OH^…^HO‐Si to SiO‐Zn‐OSi sites.

Overall, Zn K‐edge XANES combined with FT‐IR shows that zinc mainly forms dispersed Zn sites with small amount of extra‐framework ZnO_x_ sites. This finding is accompanied by a reduction of silanol defect sites, suggesting the healing of silanol nests (interacting Si‐OH) by Zn.

### 2.4 Ti Framework Site Accessibility

As recently established, ^15^N low temperature (LT) NMR analysis of pyridine adsorbed Ti‐zeotype samples can also be used as a catalytic performance descriptor [24]. In particular, the ratio of pyridine adsorbed on Lewis acid Ti sites (Ti‐py) with respect to on silanol moieties (Si‐OH‐py) probes the accessibility of Ti sites, hence correlates to catalytic performances (both in terms of activity and selectivity in the epoxidation reaction). Note that we have also previously shown that slower formation rates are typically a consequence of unavailable Ti‐sites, for example the presence of extra‐framework TiO_2_ [24]. Since for Ti‐MWW H and Ti‐MWW L, a large difference in the initial formation rate of the µ_2_‐η^2^:η^2^‐peroxo is observed, we thus evaluate the availability of Ti sites by using the aforementioned approach.

The amount of available Ti can be estimated from the Ti‐py to Si‐OH‐py ratio (this approach is only semi‐quantitative, due to the measurement conditions and considering the variation of silanol amounts for Zn treated and parent Ti‐MWW catalysts): the higher the ratio, the more available Ti sites are present. Based on this analysis we find that Ti‐MWW H shows a lower ratio (0.8, see Figure 3) than Ti‐MWW L (1.3), hence contains substantially less available Ti sites. Interestingly, the ratio is largely unaffected for ZnTi‐MWW L (1.5), whereas ZnTi‐MWW H displays in contrast to its parent Ti‐MWW H a high ratio (1.5), indicating that, for the high Ti weight loading samples, the zinc treatment is, at least partially, able to break up existing TiO_2_ domains. Notably, the higher amount of available Ti sites in ZnTi‐MWW H than Ti‐MWW H can also be derived from the Ti K‐edge XANES pre‐edge peak intensity, where the intensity is also larger for ZnTi‐MWW H (vide infra, see Figure 4b).

**FIGURE 3:**
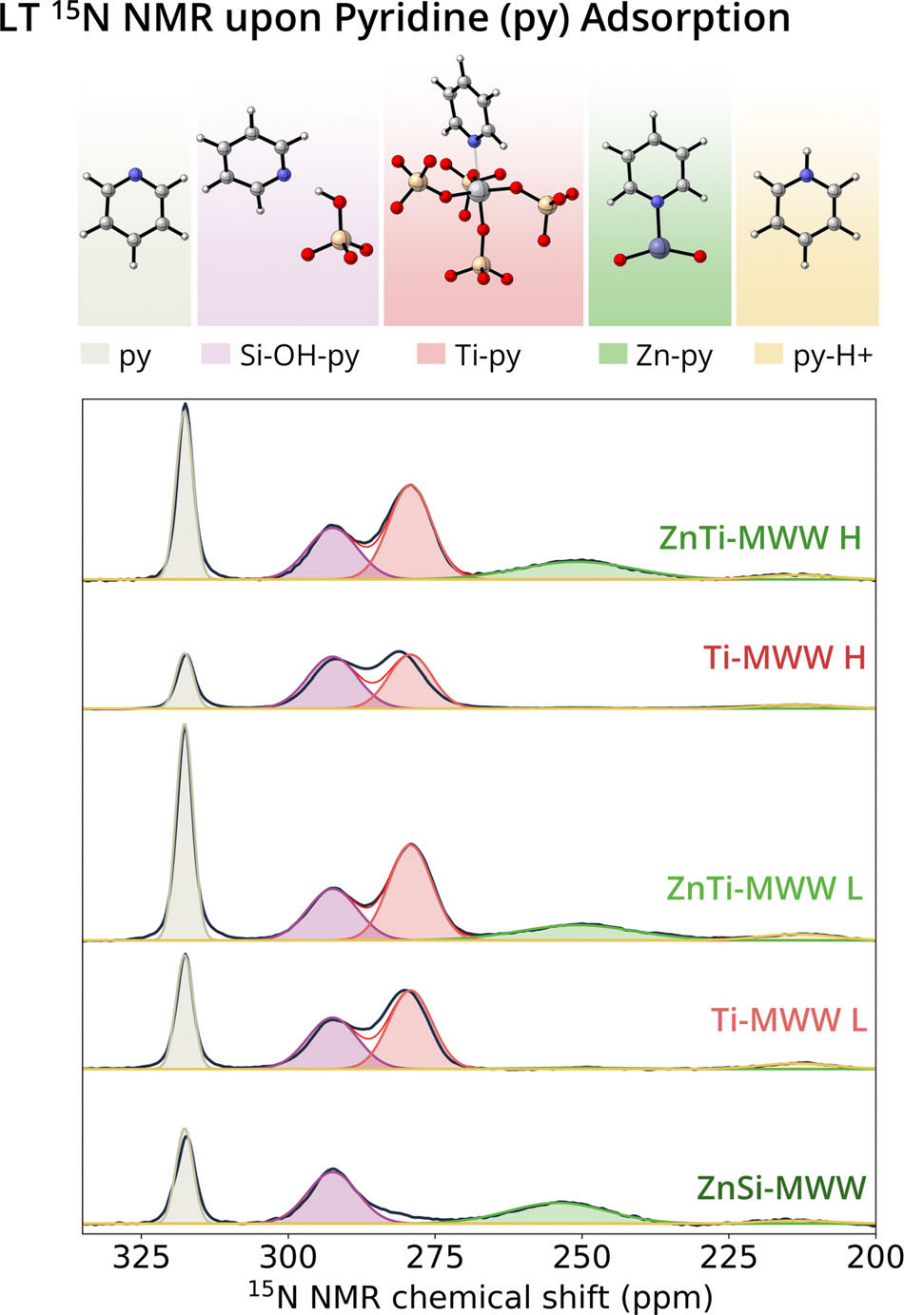
^1^H–^15^N LT CPMAS NMR spectra (8 kHz, 14.1 T, 100 K, referenced with respect to liquid NH_3_ at 0 ppm via the secondary reference glycine at 33.4 ppm at room temperature) of pyridine adsorbed on dehydrated Ti‐MWW H,L and ZnTi‐MWW H,L to assess available Ti coordination sites via Ti‐py and Si‐OH‐py ratio (normalized to the Si‐OH‐py peak) and identify Zn sites. For comparison the spectrum of pyridine on dehydrated ZnSi‐MWW is shown.

**FIGURE 4:**
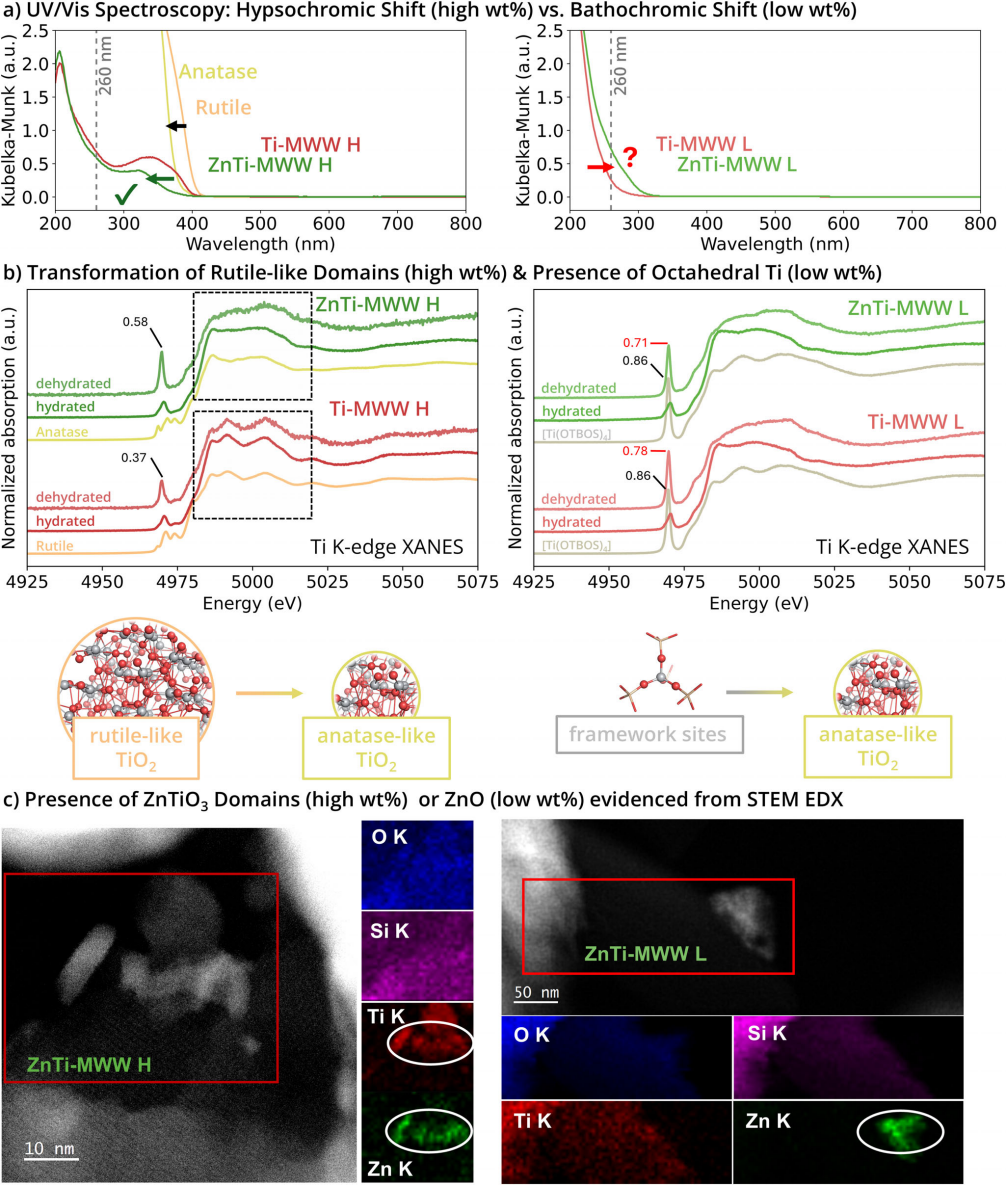
(a) DR UV–vis spectra of Ti‐MWW H,L and ZnTi‐MWW H,L and comparison to anatase and rutile nanoparticle references. (b) Ti K‐edge XANES spectra of hydrated and dehydrated Ti‐MWW H,L and ZnTi‐MWW H,L together with the molecular ([Ti(OTBOS)_4_]) and TiO_2_ nanoparticle references. (c) Representative STEM‐EDX images of ZnTi‐MWW H,L indicating the presence of ZnTiO_3_ for ZnTi‐MWW H and ZnO for ZnTi‐MWW L.

Besides, the measure of Ti site accessibility the ^15^N‐pyridine NMR enables us to identify an additional Lewis acidic species for all zinc treated samples (251 ppm). These sites are attributed to pyridine adsorbed on dispersed Lewis acidic zinc sites based on the observation of a similar peak in titanium‐free ZnSi‐MWW sample (Figure 3), closely matching the reported the ^15^N‐pyridine signal on Zn single sites [37], and in line with the Zn K‐edge spectra depicted above. However, as discussed before, Zn sites on their own are inactive in terms of the ammoximation reactivity.

Overall, ^15^N NMR data clearly shows that the amount of accessible Lewis acidic Ti‐sites can be increased upon zinc treatment (evidenced via ^15^N NMR for the high Ti weight loading samples), indicating the modification of TiO_2_ domains and substantial reorganization during the post‐treatment. Additionally, we observe the generation of Zn sites for both ZnTi‐MWW H and L identified by pyridine adsorption and in agreement with Zn K‐edge XANES.

### 2.5 Analysis of Extra‐Framework TiO_2_ in (Zn)Ti‐MWW

The performance and Ti accessibility analysis of the (Zn)Ti‐MWW samples studied herein suggests also an alteration of extra‐framework TiO_2_ for Ti‐MWW H upon zinc treatment. We have recently established that not the presence, but the specific surface structure of TiO_2_ domains defines catalytic properties [22]. In particular, whereas anatase and anatase‐like domains are able to form stable µ_2_‐η^2^:η^2^‐peroxo species, rutile, and rutile‐like domains do not stabilize peroxo surface species and are particularly efficient in decomposing H_2_O_2_ (resulting in overall low selectivity). Insights on the size and structure of extra‐framework TiO_2_ domains can be obtained from a combined analysis of UV–vis DRS and Ti K‐edge XANES white line spectroscopic signatures (for experimental details see Sections SB.4 and SB.5). For (Zn)Ti‐MWW H, the DR UV–vis spectrum shows a hypsochromic shift of the absorption edge from ca. 405 to 380 nm (see Figure 4a). Both the absorption feature itself and the magnitude of the shift are consistent with a transformation of rutile‐like TiO_2_ domains into anatase‐like TiO_2_ domains (rutile TiO_2_ has an absorption edge at 405 nm whereas anatase TiO_2_ exhibits an absorption edge at 380 nm, see Figure 4a). The corresponding Ti K‐edge XANES spectra of the (Zn)Ti‐MWW H samples as well as anatase and rutile nanoparticle references are shown in Figure 4b. The low intensity pre‐edges for the Ti‐MWW samples (accompanied by a small pre‐edge energy shift to higher energies) indicates the formation of octahedral Ti sites in the framework under ambient conditions (in contrast to TS‐1, where the hydrated sites adopt a penta‐coordinated structure) [24, 38, 39], in addition to the octahedral Ti present in the extra‐framework TiO_2_.

Thus, for the analysis of the structure of the extra‐framework TiO_2_ it is crucial to study the dehydrated materials. Ti‐MWW H displays characteristic features in the white line consistent with the presence of rutile‐like TiO_2_. For ZnTi‐MWW H, these features are no longer present (in line with the hypsochromic shift in DR UV–vis spectrum). In addition, the pre‐edge intensities of the dehydrated samples (0.37 for Ti‐MWW H and 0.58 for ZnTi‐MWW H) also suggest a size‐reduction of the TiO_2_ domains (in line with the increased Ti‐py to Si‐OH‐py ratio observed in ^15^N NMR). The combined UV–vis DRS and Ti K‐edge XANES analysis for (Zn)Ti‐MWW H suggests that zinc interacts with the extra‐framework TiO_2_. These findings can also be corroborated via scanning transmission electron microscopy and spectroscopic mapping by energy dispersive x‐ray spectroscopy (STEM‐EDX). Figure 4c shows a representative image of ZnTi‐MWW H (for additional data see Figure S30 and for STEM‐EDX of the parent Ti‐MWW H see Figure S34). As evident from the EDX mapping, zinc is concentrated in regions where also extra‐framework TiO_2_ is present, suggesting the formation of ZnTiO_3_. This is noteworthy, since ^17^O NMR shows that stable peroxo species can be formed on both anatase and ZnTiO_3_, but not on rutile (present in the parent Ti‐MWW H catalyst) where it readily decomposes. Next, we apply the same characterization approach to (Zn)Ti‐MWW L. For the low‐weight loading samples, both ^17^O NMR (fast formation of the µ_2_‐η^2^:η^2^‐peroxo) and ^15^N NMR (high Ti‐py to Si‐OH‐py ratio for both Ti‐MWW L and ZnTi‐MWW L) do not imply the presence of (larger) TiO_2_ domains (the more isolated Ti sites that pyridine can bind to, the higher the ratio of Ti‐py to Si‐OH‐py). Indeed, the DR UV–vis spectrum of Ti‐MWW L does not show any absorption feature >260 nm. Interestingly, an absorption feature at ca. 270 nm is observed for ZnTi‐MWW L (see Figure 4a), previously assigned to either isolated octahedral titanium species or amorphous hexa‐coordinated TiO_x_ species [40, 41]. The zinc treatment has thus the opposite effect: For the high Ti weight loading sample (ZnTi‐MWW H) a hypsochromic shift is observed, whereas for the low weight loading sample (ZnTi‐MWW L) a bathochromic shift is noted. The origin of the bathochromic shift is further investigated via Ti K‐edge XANES, more specifically via the analysis of pre‐edge intensities of the (Zn)Ti‐MWW L pair. Full framework incorporation is achieved for samples that show a pre‐edge intensity of 0.86 (as compared to the tetrahedral siloxide reference compound [Ti(OTBOS)_4_] (OTBOS = *tris*(*tert*‐butoxy)siloxy)) [42, 43]. While Ti‐MWW L shows pre‐dominant framework incorporation (pre‐edge intensity of 0.78, see Figure 4b), ZnTi‐MWW L shows a reduced pre‐edge intensity (0.71). The reduction of the pre‐edge intensity in Ti K‐edge XANES in combination with the bathochromic shift in the DR UV–vis spectra hence suggests that upon zinc treatment, some of the Ti is extracted from the framework to form small amorphous TiO_x_ domains or octahedral defect Ti sites. Interestingly EDX mapping also shows that ZnO domains are formed and notably, that the newly formed TiO_x_ and ZnO domains do not mix (see Figure 4c).

To summarize, analysis of the extra‐framework TiO_2_ via UV–vis DRS, Ti K‐edge XANES, and STEM‐EDX shows that for high weight loading samples zinc treatment modifies existing rutile‐like TiO_2_ domains and reduces the size of TiO_2_‐domains (with some additional redispersion of Ti‐sites). Zinc itself is shown to partially agglomerate in regions with extra‐framework TiO_2_ present. In contrast, for low weight loading samples, zinc treatment leads to the extraction of framework incorporated Ti to form small TiO_x_ domains or octahedral defect Ti sites. Crucially, zinc does not agglomerate on the newly formed TiO_2_ domains but instead forms ZnO domains. Overall, ZnTi‐MWW H and ZnTi‐MWW L become more similar after the zinc treatment, respectively, as evidenced by the ratio between framework incorporated and extra‐framework TiO_2_, suggesting dynamics between the formed species.

Next, we address the structure of the TiO_2_ surface domains via Ti L_2,3_‐edge NEXAFS (for experimental details, see Section SB.6). Ti L_2,3_‐edge spectroscopy is particularly sensitive toward octahedral species that are often challenging to assign via Ti K‐edge XANES alone (no p/d mixing in octahedron, yielding only low intensity pre‐edges). Typically, up to seven peaks can appear in Ti L_2,3_‐edge NEXAFS (for derivation, see the original publication) [23], that are denoted A1‐A5 (L_3_‐edge) and B1‐B2 (L_2_‐edge). Although Ti L_2,3_‐edge NEXAFS does not give a quantitative analysis of all species present, it readily gives insights into the surface species due to the low penetration depth of soft x‐rays. Analyzing the (Zn)Ti‐MWW H samples show a unification of the A3 feature and a small shift to lower energy for the A4 feature (see Figure 5). These results indicate that for ZnTi‐MWW H a mixture of TiO_2_ containing rutile and anatase is no longer present (unified A3) and that the resulting Ti‐speciation is overall more anatase‐like. In contrast, for (Zn)Ti‐MWW L neither of these observations is found, however, the integral ratio of A3:A5 is higher for ZnTi‐MWW L than for Ti‐MWW L. This implies that during zinc treatment small TiO_x_ domains or octahedral defect Ti sites are formed and that these species are located on the external surface of the material. Overall, Ti L_2,3_‐edge NEXAFS results are consistent with the findings of the Ti K‐edge XANES and DR UV–vis spectroscopic analysis, and indicate that the changes observed are primarily on the external zeotype surface.

**FIGURE 5:**
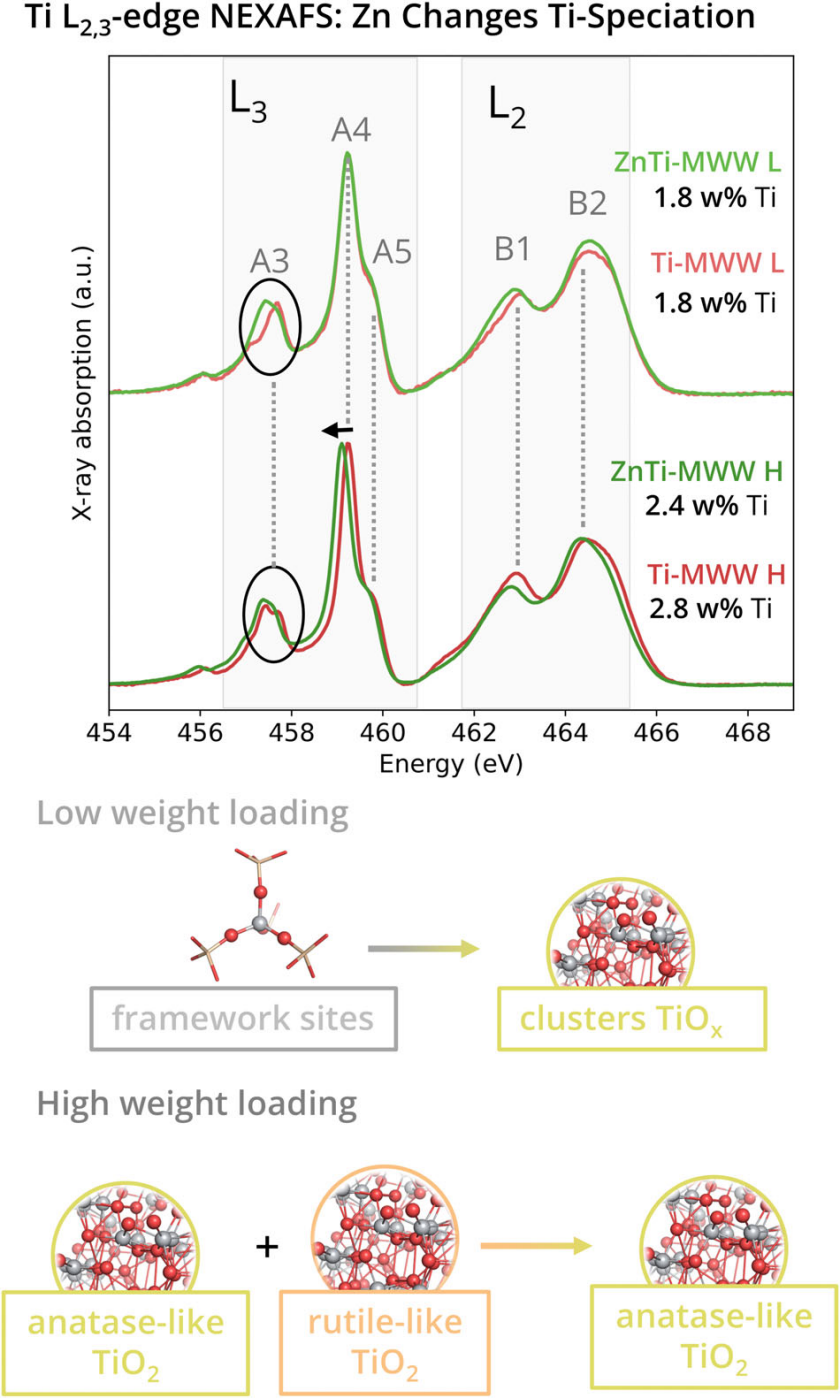
Ti L_2,3_‐edge NEXAFS spectra of Ti‐MWW H,L and ZnTi‐MWW H,L showing a change in TiO_2_ domains for ZnTi‐MWW H and formation of TiO_x_ clusters for ZnTi‐MWW L.

## 3 Discussion

Taken together, the catalytic results and the ^17^O NMR titration experiments highlight the strong influence of Zn toward (i) catalytic activity and (ii) the Ti‐peroxo formation and stability. Besides the generation of innocent dispersed Zn sites and extra‐framework Zn sites, Ti L_2,3_‐edge NEXAFS, Ti K‐edge XANES, and microscopy show that Zn also influences the Ti speciation as a function of weight loading. For low weight loading samples, Zn partially extracts Ti from the zeolite matrix and forms TiO_x_ or octahedral defect Ti sites and ZnO domains. Whereas for the high Ti‐weight loading samples Zn directly reacts with TiO_2_ surface domains, leading to the formation of ZnTiO_3_ (as evidenced by microscopy).

### 3.1 Structure‐Activity Relationships From Spectroscopic Descriptors

We thus next relate molecular insights with the catalytic activity data. First, we focus on the µ_2_‐η^2^:η^2^‐peroxo formation (ratio after 15 min based on ^17^O NMR titration) and its correlation with the acetone conversion in the ammoximation reaction (see Figure 6a). We find that in general, higher µ_2_‐η^2^:η^2^‐peroxo ratio correlates with higher conversion for both catalyst pairs, L and H, highlighting the formation of µ_2_‐η^2^:η^2^‐peroxo as descriptor of catalytic performance. This suggests that in analogy to the epoxidation reaction, the µ_2_‐η^2^:η^2^‐peroxo might as well be considered as an important resting species in the ammoximation reaction.

**FIGURE 6:**
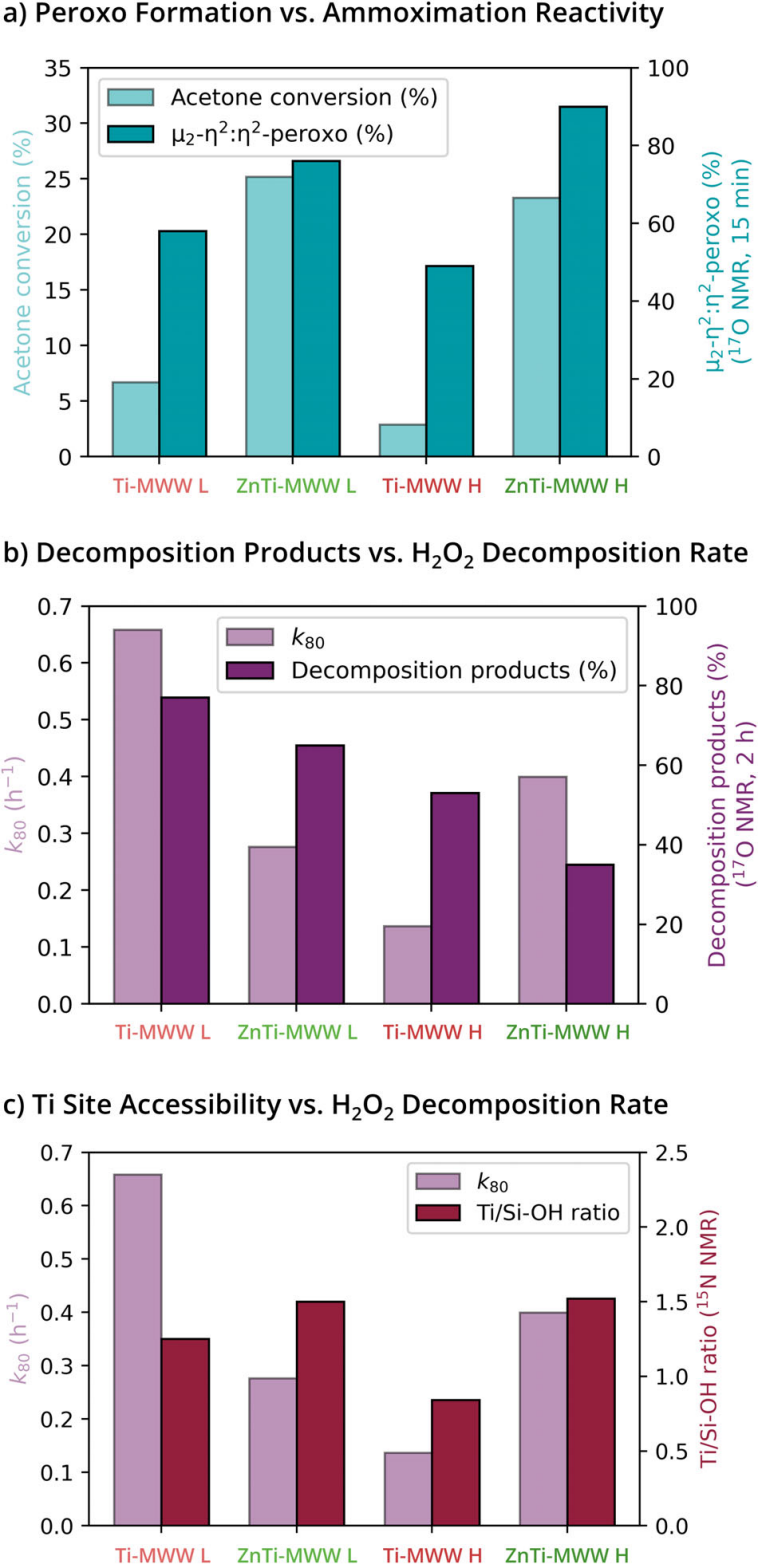
Comparison between catalytic performance data, (a) acetone conversion in the ammoximation reaction and (b) pseudo‐first order H_2_O_2_ decomposition rate constant (*k*_80_), and the ratio of oxygen species titrated on the catalyst (after 15 min or 2 h). (c) Ti site accessibility in relation to the pseudo‐first order H_2_O_2_ decomposition rate constant.

Second, regarding the H_2_O_2_ decomposition rate constant *k*_80_ and the ratio of decomposition products (after 2 h of impregnation) observed via ^17^O NMR titration within the families of (Zn)Ti‐MWW samples (H and L, see Figure 6b), both (i) the *k*_80_ value and (ii) the evolution of the oxygen species monitored by ^17^O NMR probe similar yet distinct quantities. Whereas the *k*_80_ test, performed with an excess of H_2_O_2_, probes the µ_2_‐η^2^:η^2^‐peroxo stability and Ti accessibility (accessibility might become the limiting factor) in a convoluted fashion, the ^17^O NMR measurement, performed with 1 equiv. of H_2_^17^O_2_ per Ti, is more closely monitoring the initial µ_2_‐η^2^:η^2^‐peroxo stability. Examining these quantities shows that the ratio of decomposition products and the *k*_80_ for the (Zn)Ti‐MWW L samples are in line with expectations, namely fewer decomposition products parallel low *k*_80_ values. In contrast the (Zn)Ti‐MWW H samples present the opposite trend, which we hypothesize, originates from a difference in Ti accessibility. In fact, the Ti‐py/SiOH‐py ratio (probed by ^15^N pyridine NMR) and Ti K‐edge XANES for the high Ti weight loading samples indicate that the zinc treatment leads, at least partially, to breaking up existing TiO_2_ domains, which explains the reverse trend in the decomposition rate constants. In contrast, for the low weight loading (Zn)Ti‐MWW family, Ti sites display a more similar availability, so that the peroxo stability likely dictates the decomposition rate and overall catalyst performance (see Figure 6c).

Overall, the origin of the promotion is linked to how Zn alters Ti‐speciation and silanol defects (see Scheme 2). At higher Ti weight loadings, where not all Ti can be incorporated into the framework and TiO_2_ domains are more readily available, Zn preferably agglomerates in TiO_2_ regions and can react with and break up TiO_2_ domains. Notably, Zn reacts preferably with rutile‐like TiO_2_ domains, transforming them into ZnTiO_3_‐like domains, leaving anatase‐like domains untouched, hence the improved catalytic performances.

**SCHEME 2:**
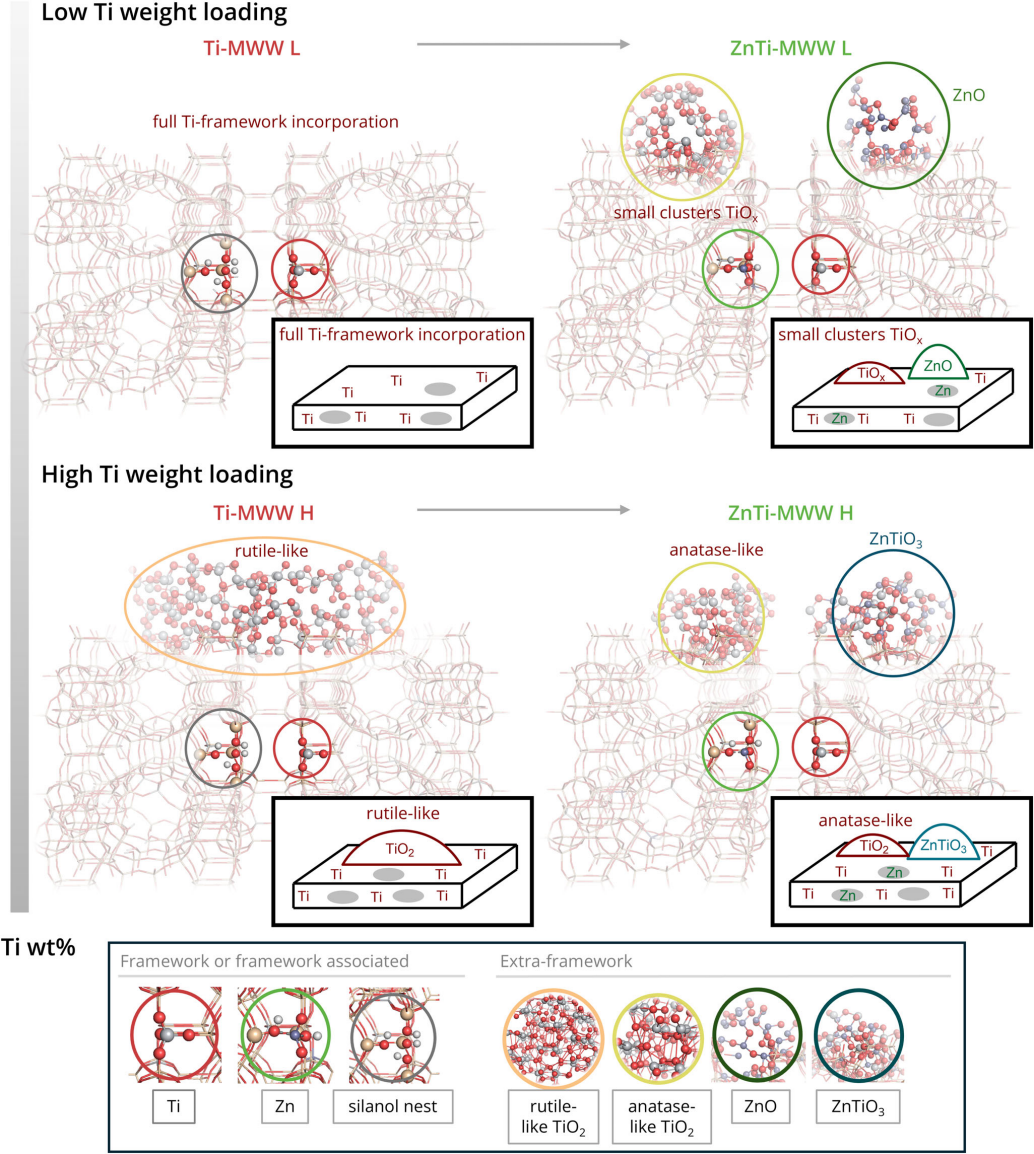
Effect of Zn treatment on Ti‐MWW samples. (top) Low Ti loading (Zn)Ti‐MWW, (bottom) high Ti loading (Zn)Ti‐MWW.

For lower Ti weight loading (Zn)TiMWW samples, when no extra‐framework TiO_2_ is available for Zn to react with, Zn removes a fraction of Ti from the framework to form small TiO_x_ domains or octahedral defect Ti sites, as evidenced by UV–vis DRS, Ti L_2,3_‐edge NEXAFS and Ti K‐edge XANES (specifically the pre‐edge intensity). Noteworthily, Zn does not mix with the emerging TiO_x_‐domains, but agglomerates to ZnO instead (see Scheme 2). In this case, ZnO is also able to participate in peroxo formation as evidenced by ^17^O NMR, but these *trans*‐peroxo species are highly stable and likely correspond to spectator species.

For both low and high Ti‐weight loading ZnTi‐MWW samples, the healing of hydrophilic sites (blockage of silanol nests with Zn) in concert with aforementioned emergence of mobile Ti (originating from small TiO_x_ clusters or octahedral defect Ti sites) are found to be the overarching phenomena and hence are proposed to enable the fast formation of stable µ_2_‐η^2^:η^2^‐peroxos, which are directly linked to increased catalyst performance. Especially the interconversion of Ti species upon Zn treatment highlights the dynamic nature of the often as *single‐site* classified Ti‐zeotype catalysts.

## 4 Conclusion

Altogether, we find that the promotional role of Zn in ZnTi‐MWW zeolite catalysts is linked to the alteration of Ti species to form small TiO_x_ clusters (precursors of mobile Ti) and the healing of hydrophilic silanol defects, increasing the formation and stability of µ_2_‐η^2^:η^2^‐peroxos. These main factors depend on the actual Ti weight‐loading. At high Ti weight loading (∼2.5 wt% Ti), where extra‐framework TiO_2_ is usually readily available, the zinc treatment transforms unfavorable rutile‐like TiO_2_ domains into ZnTiO_3_ domains, reducing the size of TiO_2_ domains and increasing the Ti site accessibility and µ_2_‐η ^2^:η^2^‐peroxo stability. At low weight loading samples (< 2.0 wt% Ti), the zinc treatment has the opposite effect, namely it generates small TiO_x_ domains from framework incorporated tetrahedral Ti‐sites, while zinc itself agglomerates to small ZnO clusters. However, independent of the Ti weight loading Zn treatment boosts activity, which we attribute to the common factors of mobile Ti originating from small TiO_x_ clusters or octahedral defect Ti sites and the healing of defect silanol sites.

Overall, this study further highlights the dynamic nature of Ti‐zeotypes and yields a rational understanding for post‐synthetic modifications and provides detailed structure‐activity relationships in Zn‐promoted Ti‐zeotype oxidation catalysts. These findings are expected to be translatable to other selective oxidation reactions, where H_2_O_2_ activation/decomposition is also a key parameter, such as the epoxidation of olefins.

## Author Contributions

**Christoph J. Kaul**: conceptualization, investigation, writing – original draft, methodology, validation, writing – review and editing, formal analysis. **Lukas Lätsch**: conceptualization, investigation, writing – original draft, methodology, validation, writing – review and editing, formal analysis. **Alexander V. Yakimov**: conceptualization, investigation, methodology, writing – review and editing. **Milivoj Plodinec**: investigation, methodology, writing – review and editing. **Xing Huang**: investigation. **Trees De Baerdemaeker**: investigation. **Jaroslaw Mormul**: investigation. **Peter Oechsle**: investigation. **Andrei–Nicolae Parvulescu**: conceptualization, investigation, project administration, writing – review and editing. **J. Henrique Teles**: conceptualization, investigation, writing – review and editing, project administration. **Christophe Copéret**: conceptualization, investigation, funding acquisition, writing – original draft, methodology, writing – review and editing, formal analysis, project administration, supervision.

## Conflicts of Interest

The authors declare no conflicts of interest.

## Supporting information



Experimental details include synthetic protocols of (Zn)TiMWW catalysts and additional characterizations (pXRD, surface area, FT‐IR, DR UV–vis, and XAS), catalytic performance tests (H_2_O_2_ decomposition and ammoximation reaction), ^17^O NMR quantification, ^15^N NMR measurements, microscopy analysis, and computational details.**Supporting File 1**: anie74240‐sup‐0001‐SuppMat.pdf.

**Supporting File 2**: anie74240‐sup‐0002‐SuppMat.zip.

## Data Availability

The data that support the findings of this study are available on request from the corresponding author. The data are not publicly available due to privacy or ethical restrictions.
