# Supplementary material for: Post‐Synthetic Zinc Modifications to Correct Speciation in Ti‐MWW Zeolites for Catalytic Oxidation
Source: Angew Chem Int Ed Engl. 2026 Aug 23;65(42):e2516685. doi: 10.1002/anie.2516685 (PMC13653587; doi:10.1002/anie.2516685)
Supplement: Supplementary file 1 [file ANIE-65-e2516685-s002.pdf]

# Supporting Information

# Post-Synthetic Zinc Modifications to Correct Speciation in Ti-MWW Zeolites for Catalytic Oxidation

Christoph J. Kaul,[a,+] Lukas Lätsch,[a,+] Alexander V. Yakimov,[a] Milivoj Plodinec,[a,b] Xing Huang,[a,b] Trees De Baerdemaeker,[c] Jaroslaw Mormul,[c] Peter Oechsle,[c] Andrei-Nicolae Parvulescu,[c] J. Henrique Teles,[c] Christophe Copéret[a,*]

[+] CJK and LL contributed equally to this work.

a Department of Chemistry and Applied Biosciences, ETH Zurich, Vladimir-Prelog Weg 2, 8093 Zurich, Switzerland

b Scientific Center for Optical and Electron Microscopy (ScopeM), ETH Zurich, Otto-Stern-Weg 3, 8093 Zurich, Switzerland

c BASF SE, Carl-Bosch-Str. 38, 67056 Ludwigshafen, Germany

**Corresponding Author**
*Christophe Copéret ccoperet@ethz.ch

# Contents


**SA Synthesis** S1

SA.1 Synthetic Procedure for (Zn)Ti-MWW L and (Zn)Ti-MWW H . . . S1

SA.1.1 Ti-MWW L and ZnTi-MWW L . . . S1

SA.1.2 Ti-MWW H and ZnTi-MWW H . . . S1

SA.2 ZnSi-MWW . . . S1

SA.3 Synthetic Procedure of Zn Single Sites . . . S1

SA.4 Commercial Details for $TiO_2$, $ZnTiO_3$ and ZnO Nanoparticles . . . S1

**SB Characterization** S2

SB.1 pXRD Characterization of (Zn)Ti-MWW L and (Zn)Ti-MWW H . . . S2

SB.2 Surface Area Determination . . . S4

SB.3 FT-IR Measurements of (Zn)Ti-MWW L and (Zn)Ti-MWW H . . . S4

SB.4 DR UV-Vis Spectra of (Zn)Ti-MWW L and (Zn)Ti-MWW H . . . S4

SB.5 Ti K-edge XANES Characterization of (Zn)Ti-MWW L and (Zn)Ti-MWW H . . . S5

SB.5.1 Ti K-edge XANES of hydrated and dehydrated (Zn)Ti-MWW . . . S5

SB.5.2 Ti K-edge XANES of Ti-MWW L upon pyridine adsorption . . . S6

SB.5.3 Ti K-edge XANES of (Zn)Ti-MWW H upon impregnation with aqueous $H_2O_2$ . . . S7

SB.6 Ti $L_{2,3}$-edge NEXAFS Characterization of (Zn)Ti-MWW L and (Zn)Ti-MWW H . . . S7

SB.7 Zn K-edge XANES Characterization of ZnTi-MWW L and ZnTi-MWW H . . . S7

**SC Catalytic Performance** S9

SC.1 (Zn)Ti-MWW Catalyzed $H_2O_2$ Decomposition Rate . . . S9

SC.1.1 Experimental Procedure . . . S9

SC.1.2 Analytics . . . S9

SC.1.3 Data Analysis . . . S9

SC.2 (Zn)Ti-MWW Catalyzed Ammoximation Reaction . . . S11

**SD $^{17}O$ NMR Quantification of Peroxo Formation and Stability** S12

SD.1 Experimental Details $^{17}O$ NMR Spectroscopy . . . S12

SD.2 $\mu_2$-$\eta^2$:$\eta^2$-Peroxo Formation and Stability on (Zn)Ti-MWW L and (Zn)Ti-MWW H . . . S12

SD.3 Investigated Peroxo Formation on ZnSi-MWW . . . S14

SD.4 Peroxo Formation and Stability on $ZnTiO_3$ and ZnO . . . S14

**SE $^{15}N$ Low Temperature NMR upon Pyridine Adsorption** S16

SE.1 Adsorption of $^{15}N$-pyridine . . . S16

SE.2 Experimental Details $^{15}N$ NMR Spectroscopy . . . S16

SE.3 Recorded $^{15}N$ NMR LT Spectra and Deconvoluted Signals . . . S17

**SF Microscopy Analysis of (Zn)Ti-MWW L and (Zn)Ti-MWW H** S22

SF.1 Experimental Details Microscopy . . . S22

SF.2 Additional STEM of (Zn)Ti-MWW L and (Zn)Ti-MWW H . . . S22

SF.3 HRTEM of (Zn)Ti-MWW L . . . S23

SF.4 EDX Mapping of (Zn)Ti-MWW L and (Zn)Ti-MWW H . . . S23

**SG Computational Details** S25

SG.1 General Details for Geometry Optimization and $^{17}O$ NMR Calculations . . . S25

SG.1.1 Geometry Optimization . . . S25

SG.1.2 $^{17}O$ NMR Parameter Calculations . . . S25

SG.2 Benchmark $^{17}O$ NMR Parameter Calculations . . . S25

SG.2.1 $^{17}O$ Isotropic Chemical Shift . . . S25

SG.2.2 $^{17}O$ Chemical Shift Anisotropy . . . S26

SG.2.3 $^{17}O$ Quadrupole Coupling Constant . . . S27

SG.3 Models for Peroxo Formation on Ti Sites in Ti-MWW . . . S27

SG.4 Models for Peroxo Formation on $ZnTiO_3$ and ZnO . . . S28

**SH Additional Software Packages** S29

**References** S30

# SA Synthesis

## SA.1 Synthetic Procedure for (Zn)Ti-MWW L and (Zn)Ti-MWW H

### SA.1.1 Ti-MWW L and ZnTi-MWW L

Ti-MWW L and ZnTi-MWW L were prepared following a recipe described in Reference 1.

### SA.1.2 Ti-MWW H and ZnTi-MWW H

The Ti-MWW H studied herein (Sample Ti-MWW B in Reference 2, 2.8 wt% Ti) and ZnTi-MWW H were synthesized according to a recipe described in Reference 3.

## SA.2 ZnSi-MWW

In a round bottom flask containing 400 g of demineralized water, 9.15 g of $Zn(OAc)_2 \cdot 2\,H_2O$ were added under stirring. The Zn salt is completely dissolved, before 50 g of a Si-MWW prepared according to a recipe described in Reference 1 are added under stirring. The suspension is than heated to 100 °C and kept at this temperature for 4 h under mechanical stirring. Afterwards the suspension is cooled down to room temperature and the solids are separated by filtration, washed intensively with demineralized water, dried for 4 h by 120 °C, before the temperature is increased for calcination at 550 °C (heating ramp of 2 °C/min) for 5 h under an air atmosphere.

## SA.3 Synthetic Procedure of Zn Single Sites

Zn single sites on silica, Zn@$SiO_2$ (0.8 Zn atoms per $nm^2$), were synthesized according to literature procedure.[4]

## SA.4 Commercial Details for $TiO_2$, $ZnTiO_3$ and ZnO Nanoparticles

$TiO_2$ anatase (Sigma Aldrich 637254, LOT: MKBP2332V), $TiO_2$ rutile (Sigma Aldrich 637262, LOT: BCBF1496V), $ZnTiO_3$ (Sigma Aldrich 634409, LOT: MKCL4119) and ZnO (Alfa Aesar 087812, LOT: W12A051) nanopowders were acquired from Sigma Aldrich and Thermo Scientific Alfa Aesar, respectively, and used without further treatment.

# SB Characterization

## SB.1 pXRD Characterization of (Zn)Ti-MWW L and (Zn)Ti-MWW H

Powder XRD data were collected using a diffractometer (D8 Advance Series II, Bruker AXS) equipped with a LYNXEYE detector operated with a copper anode X-ray tube, running at 40 kV and 40 mA. The geometry was Bragg–Brentano, and air scattering was reduced using an air-scatter shield. For measurements, the samples were homogenized in a mortar and then pressed into a standard flat sample holder provided by Bruker AXS for Bragg–Brentano geometry data collection. The flat surface was achieved using a glass plate to compress and flatten the sample powder. The data were collected from the angular range 2°–70° $2\theta$, with a step size of 0.02° $2\theta$; the variable divergence slit was set to an angle of 0.1°.

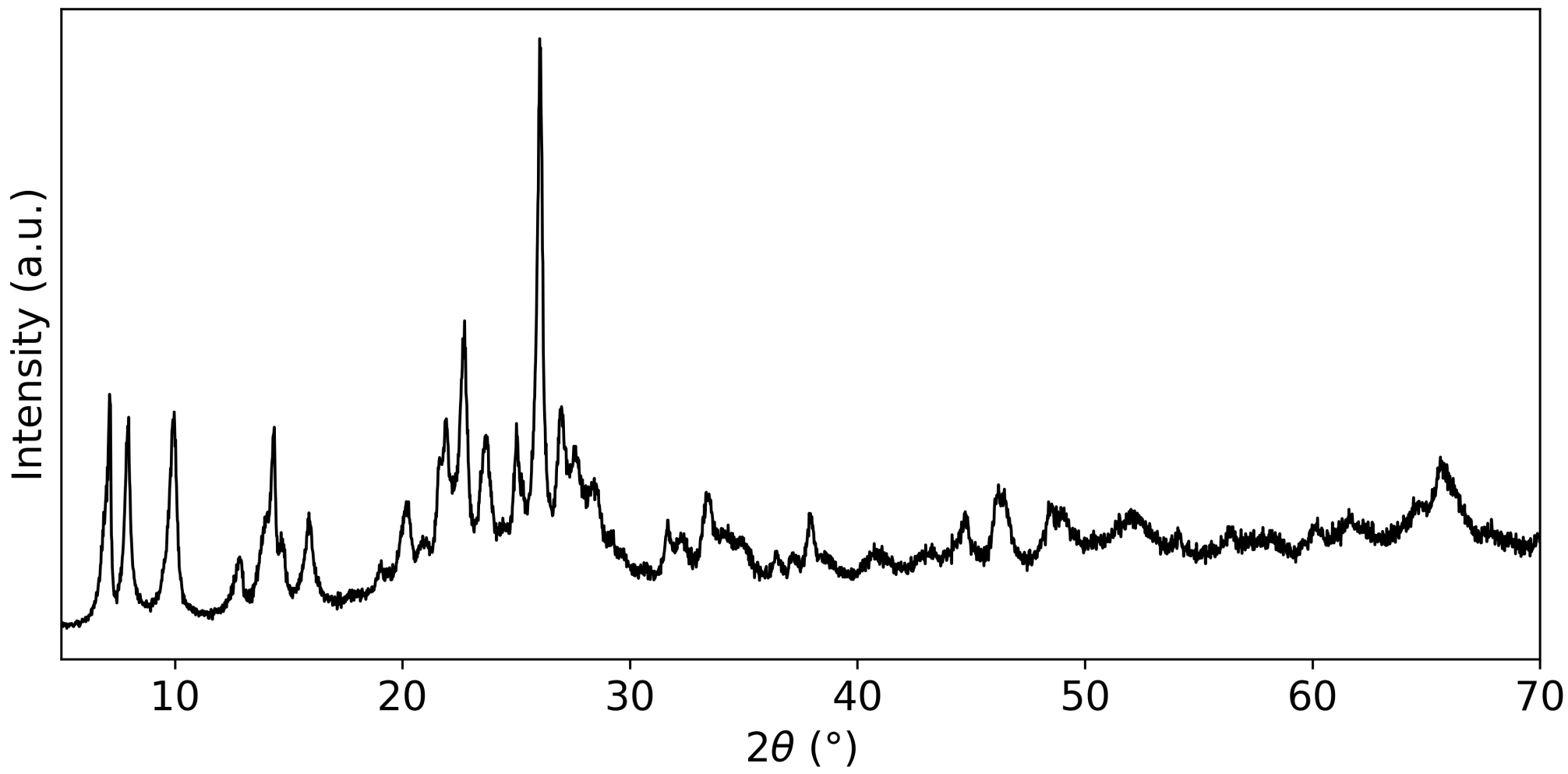


**Figure S1:** Powder XRD pattern of Ti-MWW L.

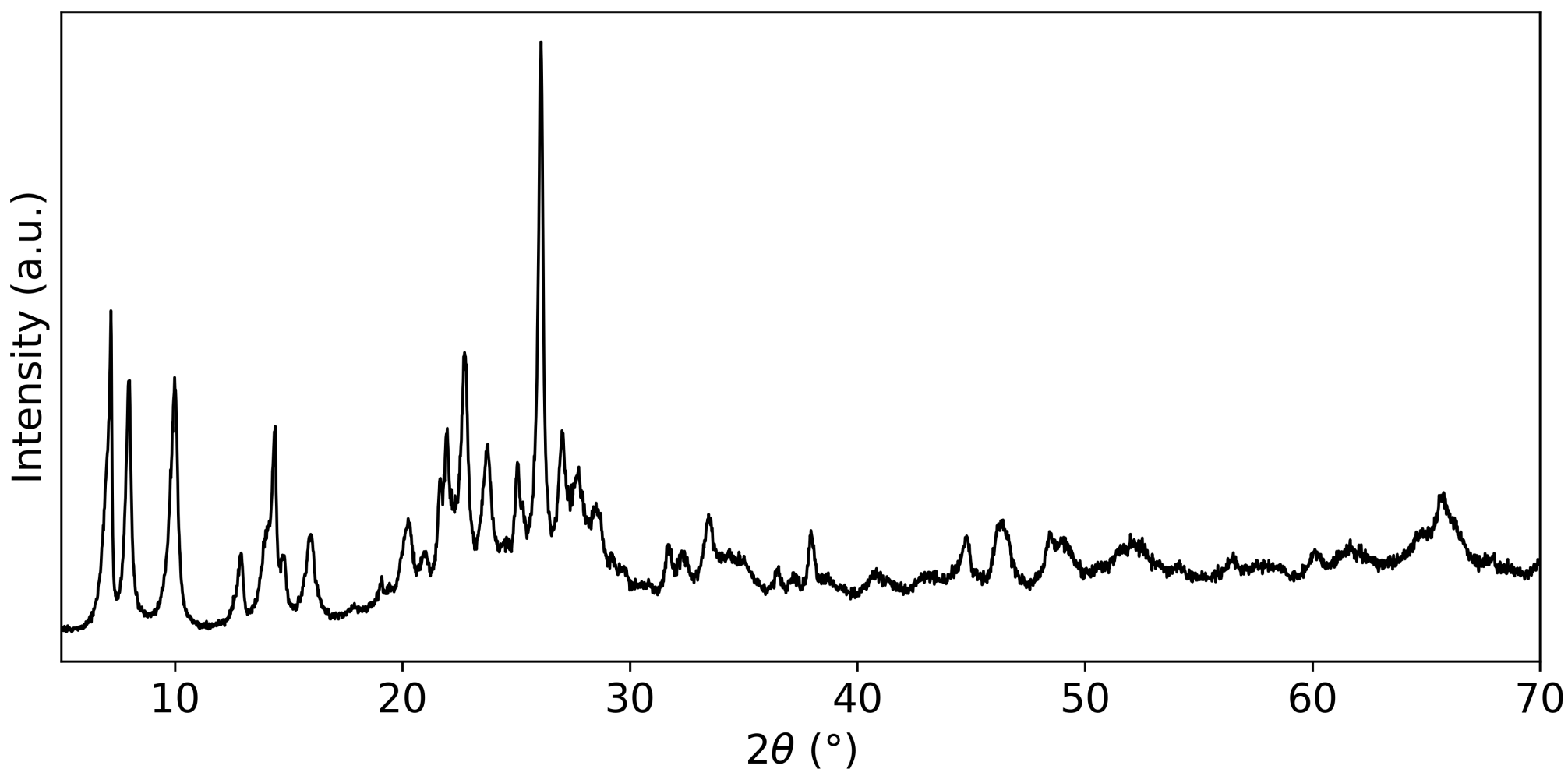


**Figure S2:** Powder XRD pattern of ZnTi-MWW L.

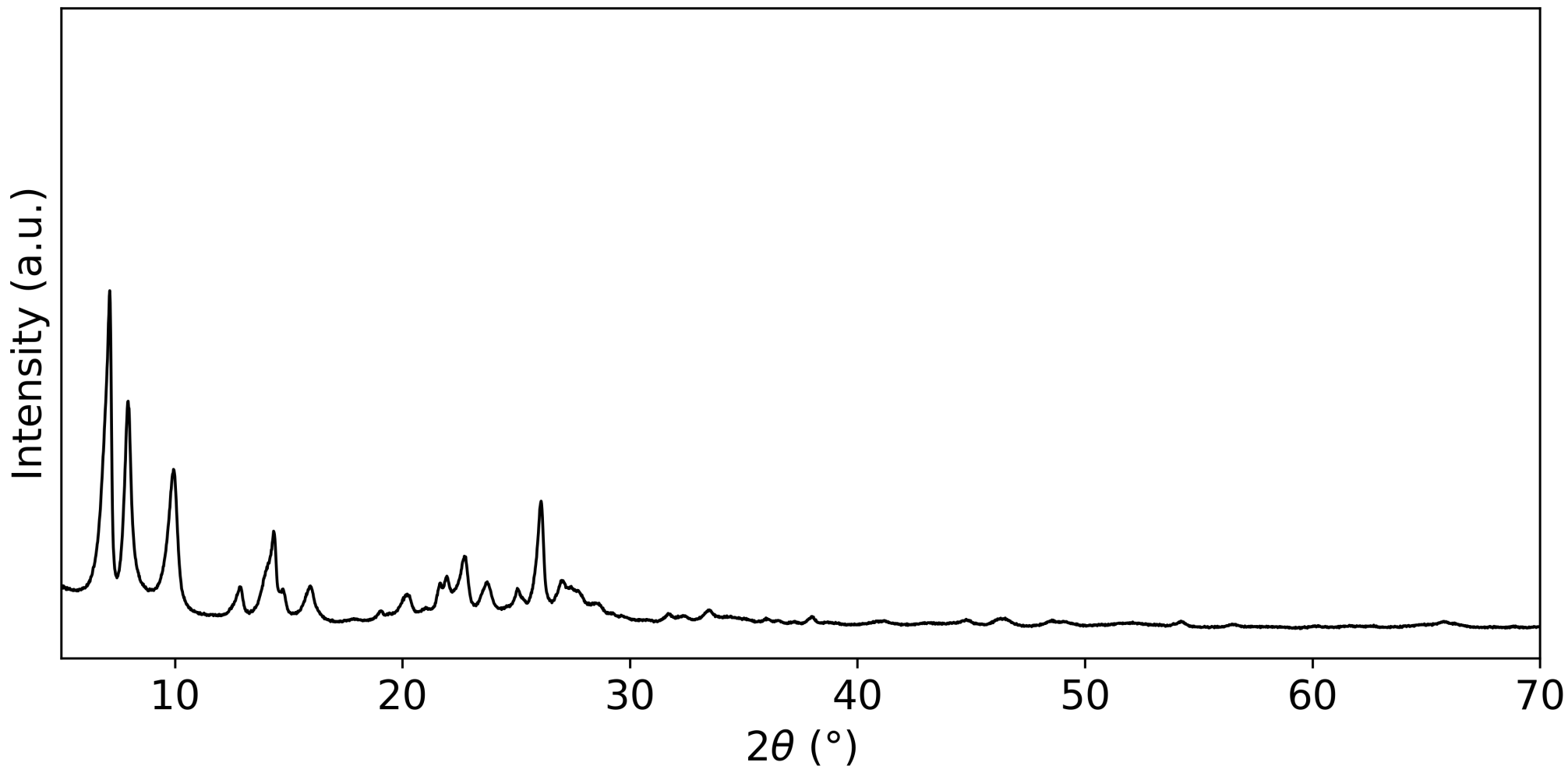


**Figure S3:** Powder XRD pattern of Ti-MWW H. The powder XRD data was already previously reported in Reference 2.

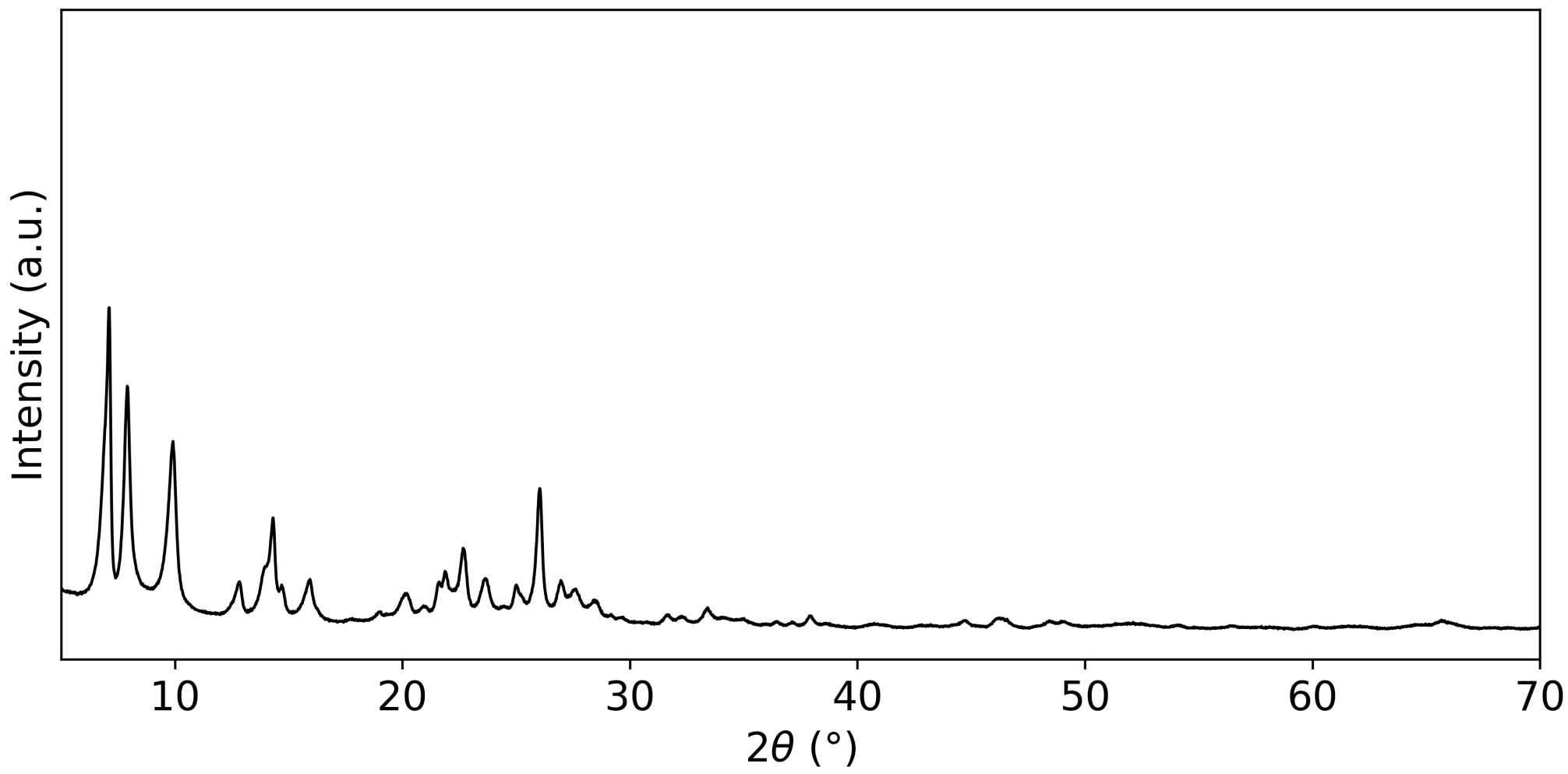


**Figure S4:** Powder XRD pattern of ZnTi-MWW H.

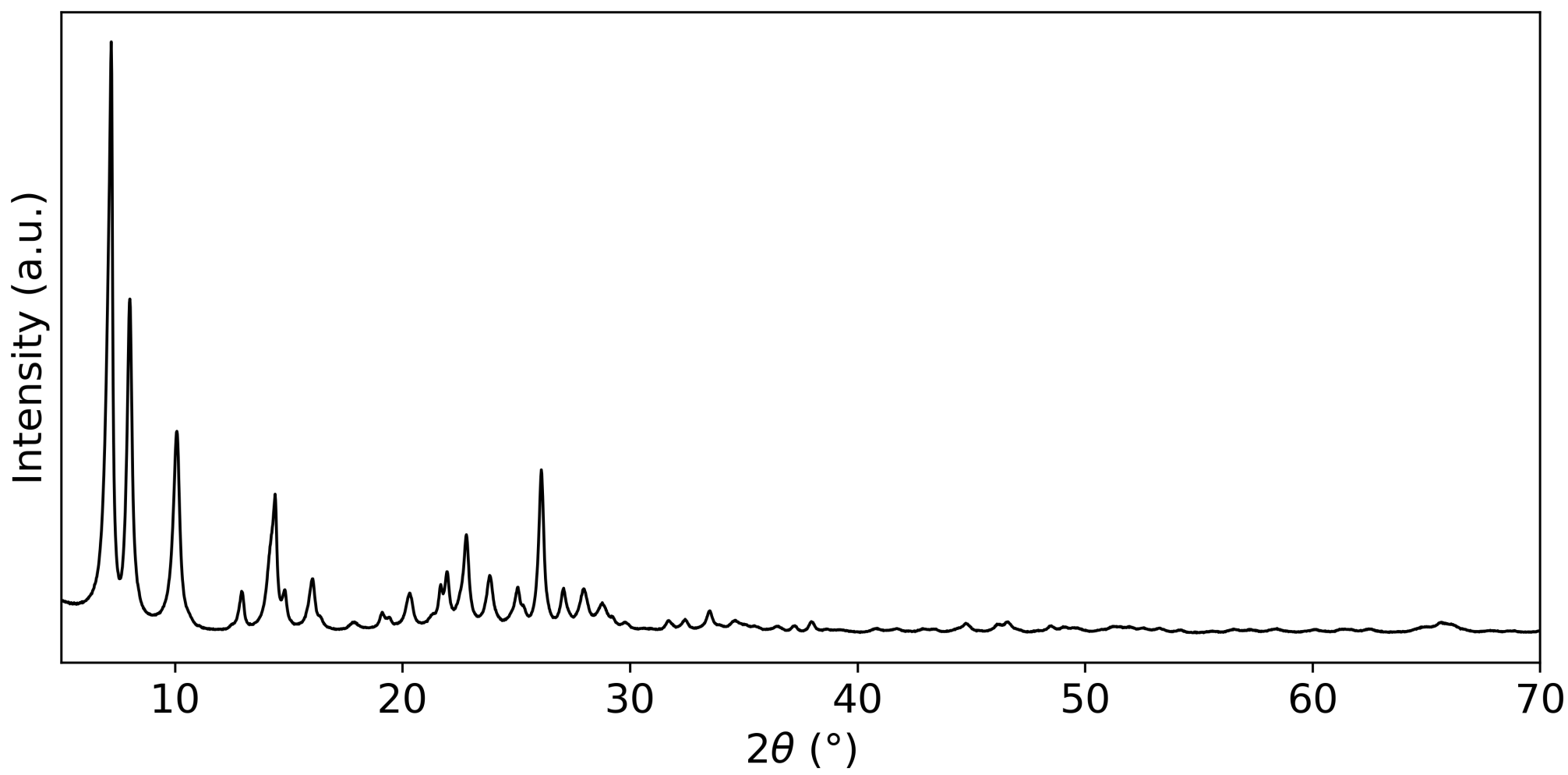


**Figure S5:** Powder XRD pattern of ZnSi-MWW.

## SB.2 Surface Area Determination

$N_2$ adsorption isotherms to determine the BET surface areas for the zeotype samples studied herein were collected on a Micromeritics TriStar device after outgassing the samples for 16 h at 200 °C under vacuum.

- Ti-MWW L: 459 $m^2/g$
- ZnTi-MWW L: 440 $m^2/g$
- Ti-MWW H: 514 $m^2/g$ (reported in Reference 2)
- ZnTi-MWW H: 568 $m^2/g$
- ZnSi-MWW: 461 $m^2/g$

The BET surface area of $ZnTiO_3$ and ZnO was determined from a $N_2$ physisorption isotherm recorded at 77 K on a BEL JAPAN BELSORP-mini II apparatus. The samples were degassed at 300 °C under vacuum ($10^{-3}$ mbar) for 5 h prior to measurement. The data was analyzed by the BET method with a $p/p_0$ range between 0.05 and 0.25.

- $ZnTiO_3$: 9.7 $m^2/g$
- ZnO: 4.1 $m^2/g$

## SB.3 FT-IR Measurements of (Zn)Ti-MWW L and (Zn)Ti-MWW H

All infrared (IR) spectra were recorded using a Bruker $\alpha$-T spectrometer placed in an Ar glovebox, equipped with OPUS software. The zeotype samples were pressed into self-standing pellets. A typical experiment consisted in the measurement of transmission spectra in 24 scans in the region from 4000 to 400 $cm^{-1}$.

## SB.4 DR UV-Vis Spectra of (Zn)Ti-MWW L and (Zn)Ti-MWW H

The Diffuse Reflectance (DR) UV-Vis measurements were performed with a PerkinElmer Lambda 950 instrument. The measurements were performed at room temperature, in the spectral range 200 to 800 nm with an integration time of 0.2 s and a scan speed of 267 nm/min. Before the measurement the powder sample was pressed into a powder cuvette for solid samples.

## SB.5 Ti K-edge XANES Characterization of (Zn)Ti-MWW L and (Zn)Ti-MWW H

Ti K-edge XANES (X-ray absorption near edge structure) spectra were recorded at the European Synchrotron Radiation Facility (Grenoble, France). The X-ray beam was monochromatized using a liquid nitrogen cooled Si(111) monochromator. The energy was calibrated at 4966 eV using the first inflection point of the Ti K-edge of a Ti metal foil standard material.[5] The XAS Ti K-edge measurements were carried out with a fluorescent scheme of detection using a silicon drift diode with associated digital electronics. Anatase and rutile nanoparticle references were measured as pellets prepared at ambient conditions and were mixed with cellulose (1:2 w%) prior to the pellet preparation in order to reduce Ti concentration. The Ti K-edge XANES spectra of anatase and rutile nanoparticle references were previously reported in Reference 2.

### SB.5.1 Ti K-edge XANES of hydrated and dehydrated (Zn)Ti-MWW

Ti-MWW L, ZnTi-MWW L, Ti-MWW H and ZnTi-MWW H were measured as pellets prepared at ambient conditions and in sealed quartz capillaries (dehydrated overnight at 400 °C). The Ti K-edge XANES spectrum of Ti-MWW H in the hydrated state was previously reported in Reference 2.

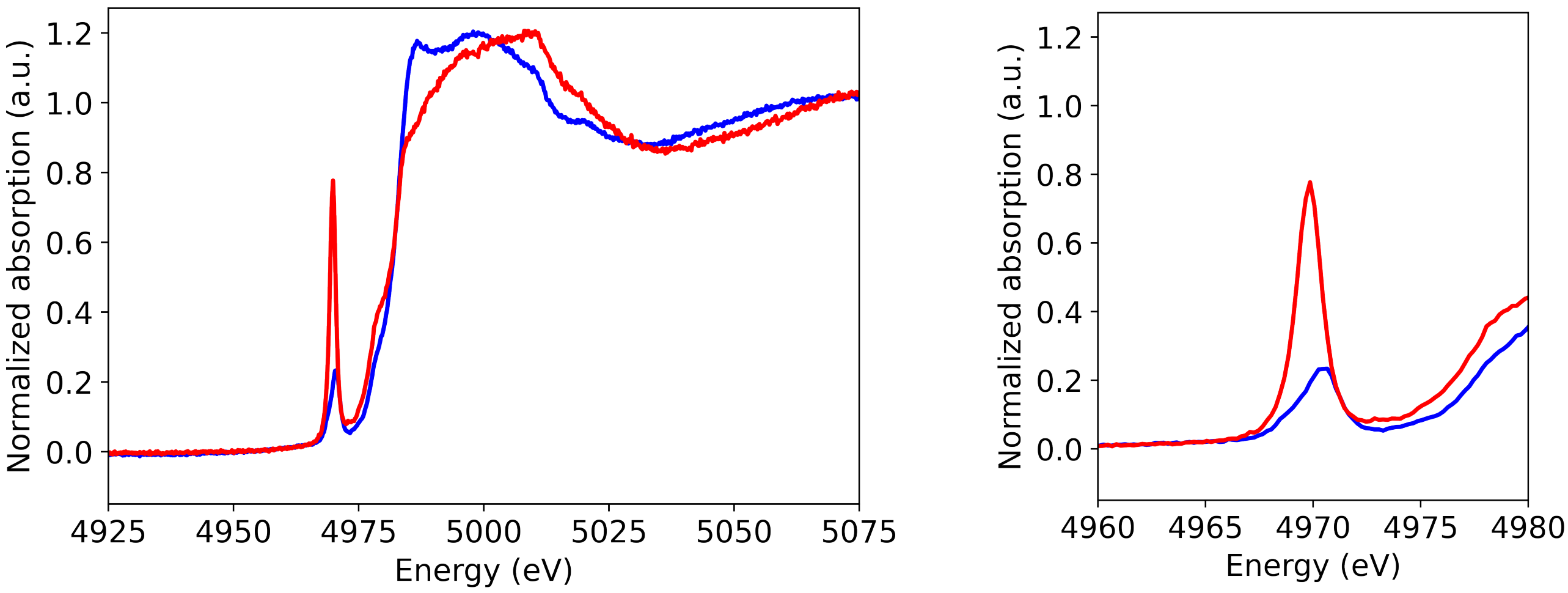


**Figure S6:** Ti K-edge XANES spectra of Ti-MWW L in the hydrated (blue) and dehydrated state (red).

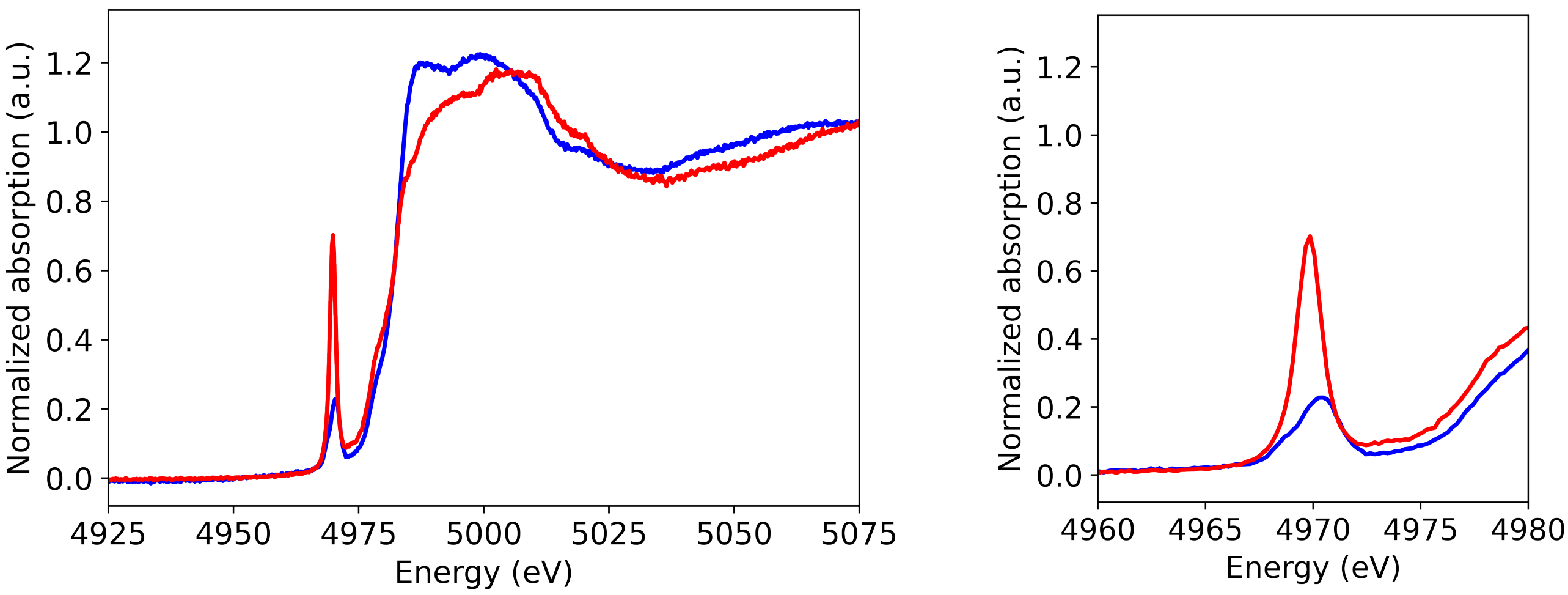


**Figure S7:** Ti K-edge XANES spectra of ZnTi-MWW L in the hydrated (blue) and dehydrated state (red).

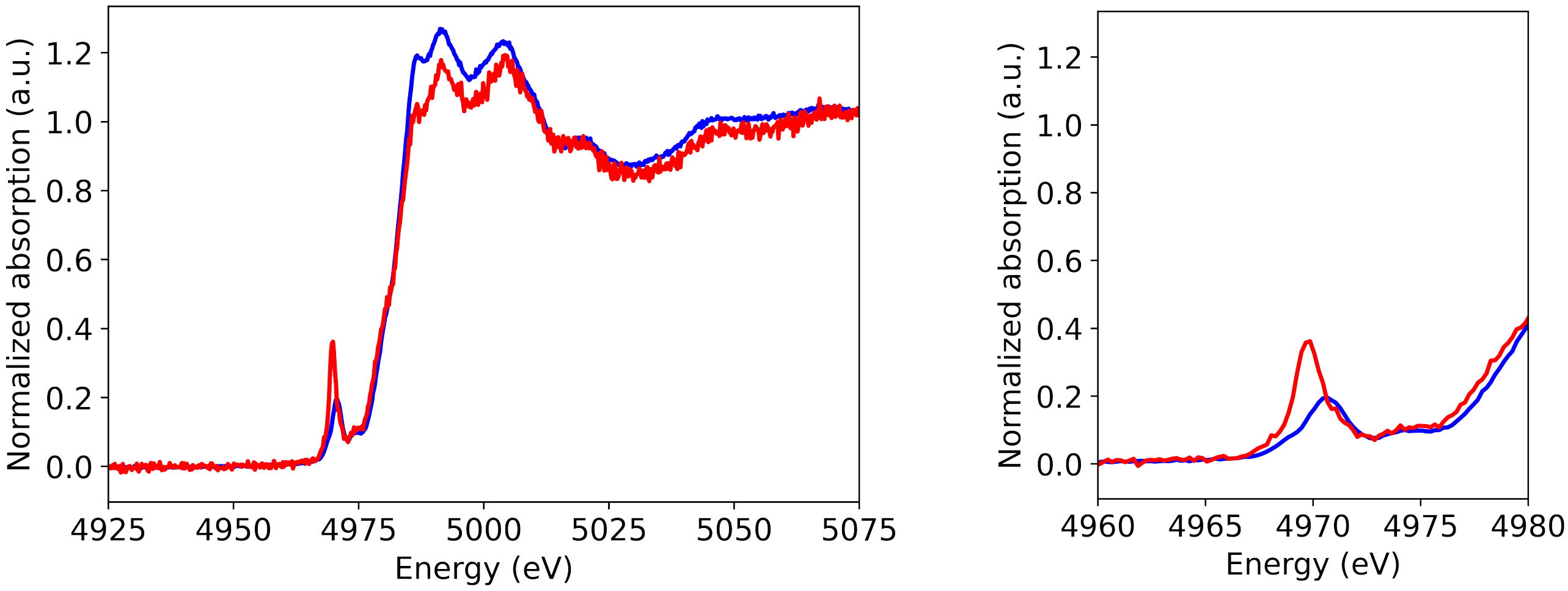


**Figure S8:** Ti K-edge XANES spectra of Ti-MWW H in the hydrated[2] (blue) and dehydrated state (red).

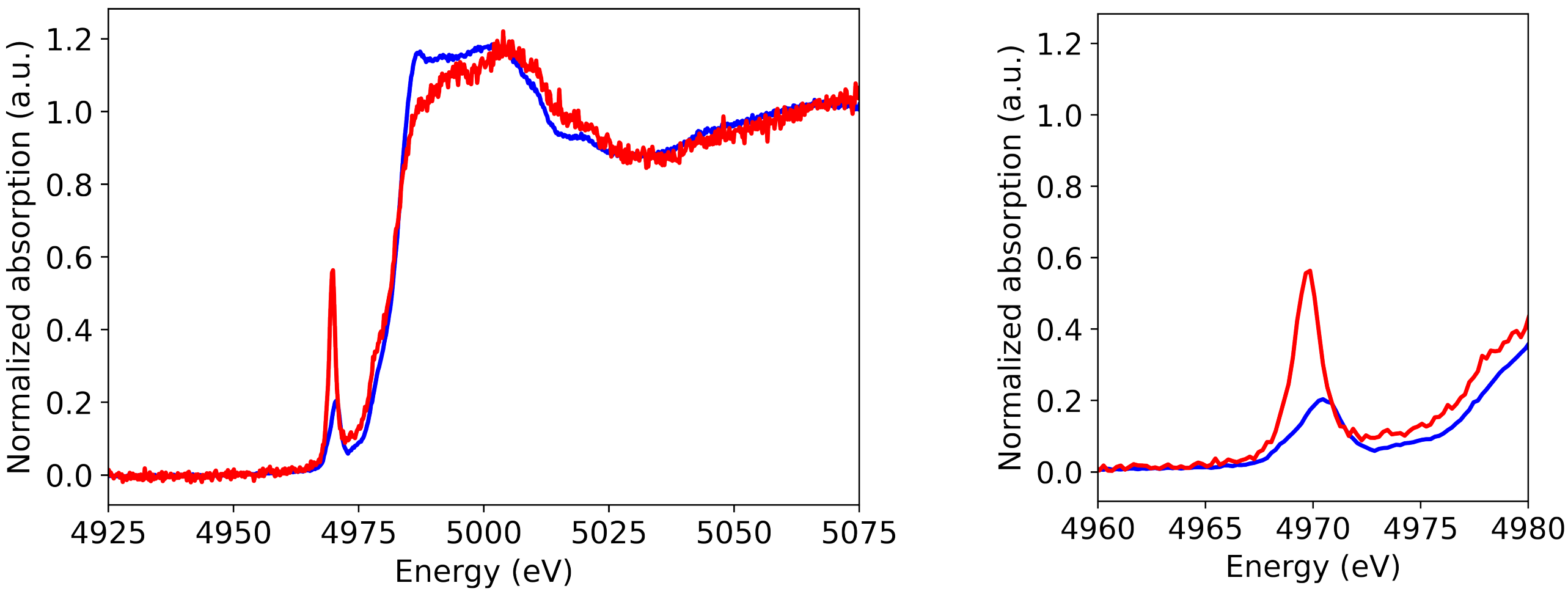


**Figure S9:** Ti K-edge XANES spectra of ZnTi-MWW H in the hydrated (blue) and dehydrated state (red).

### SB.5.2 Ti K-edge XANES of Ti-MWW L upon pyridine adsorption

The pyridine adsorption of the Ti-MWW L sample was performed as discussed in Section SE.1.

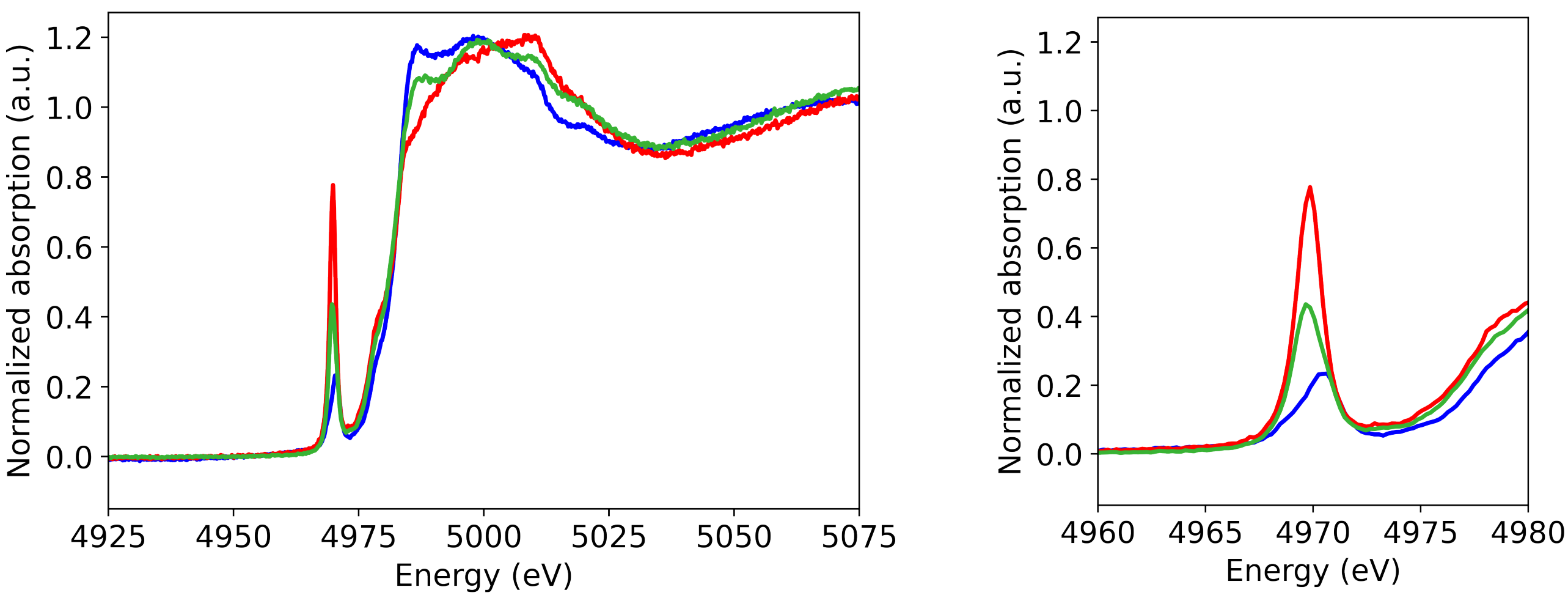


**Figure S10:** Ti K-edge XANES spectra of Ti-MWW L in the hydrated state (blue), dehydrated state (red) and upon pyridine adsorption (green).

### SB.5.3 Ti K-edge XANES of (Zn)Ti-MWW H upon impregnation with aqueous $H_2O_2$

The (Zn)Ti-MWW H samples were impregnated with 1 equiv. $H_2O_2$ per Ti. Therefore 75 mg of the catalysts were impregnated with the corresponding amount of a 1.6 M aq. $H_2O_2$ solution (see Table S1). The samples were directly transferred to the sample holder and the initial measurement was immediately performed ($t_0 = 0$ h).

**Table S1:** Sample preparation of (Zn)Ti-MWW H samples impregnated with aqueous $H_2O_2$ solution for Ti K-edge XANES measurements.

| Sample | mass of catalyst (mg) | Ti wt% | volume of aq. $H_2O_2$ solution ($c = 1.6$ M) (µL) |
|---|---|---|---|
| Ti-MWW H | 75 | 2.8 | 27.5 |
| ZnTi-MWW H | 75 | 2.4 | 23.5 |

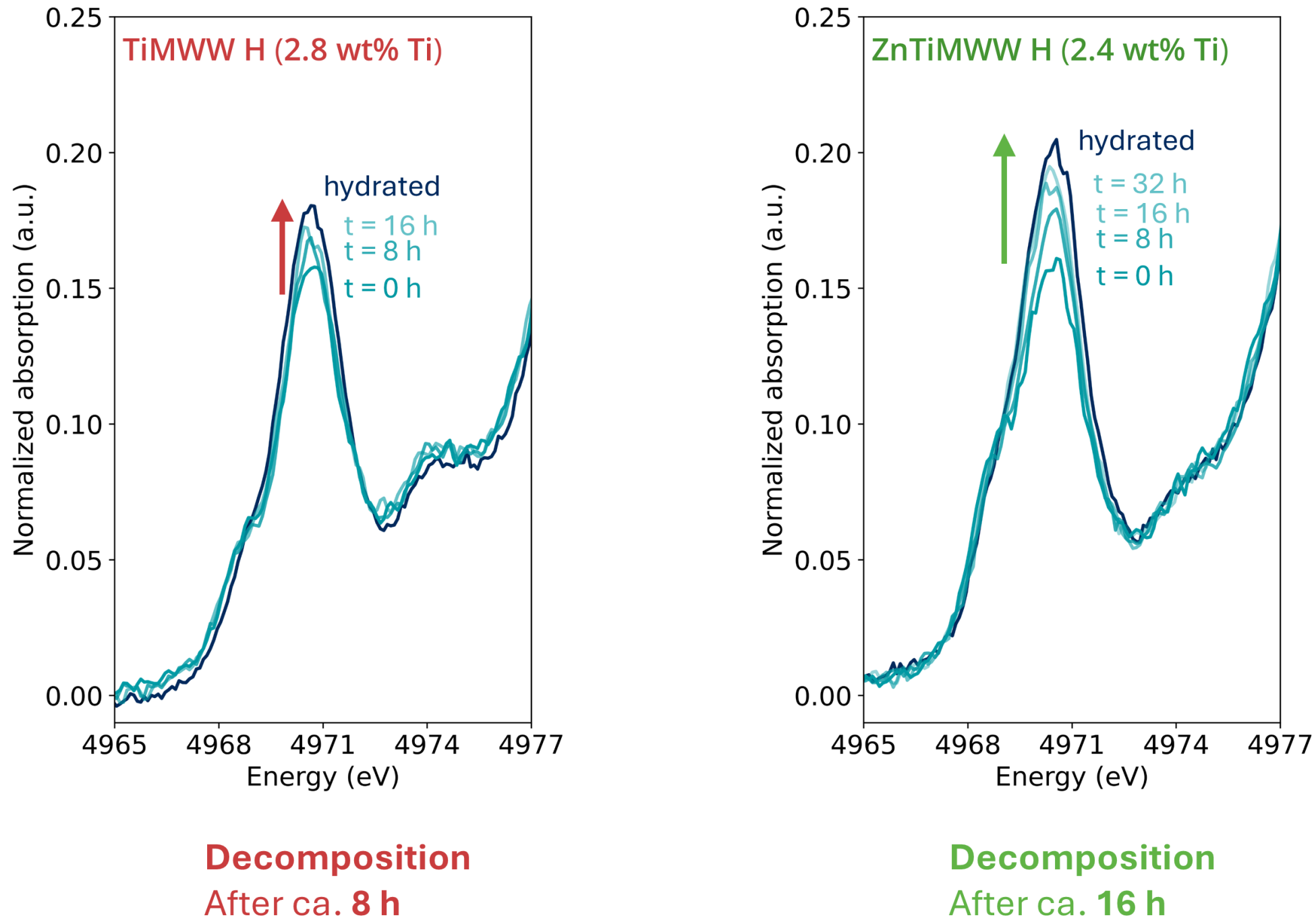


**Figure S11:** Ti K-edge XANES spectra of Ti-MWW and ZnTi-MWW H upon impregnation with aqueous $H_2O_2$, measured at different time points.

## SB.6 Ti $L_{2,3}$-edge NEXAFS Characterization of (Zn)Ti-MWW L and (Zn)Ti-MWW H

Ti $L_{2,3}$-edge NEXAFS (Near Edge X-ray Absorption Spectroscopy) spectra were recorded at the X-Treme beam line at the Swiss Light Source (SLS, PSI, Villigen, Switzerland).[6] The X-ray beam was used with normal incidence on the sample and the total electron yield (TEY) of the sample was measured. The total electron yield detection is performed by measuring the drain current induced by the absorption of X-rays and the subsequent ejection of electrons, as described in, e.g. Reference 7. A small (static) magnetic field of 50 mT parallel to the beam to enhance the yield was utilized. The magnetic field was always collinear with the X-ray beam direction. The powder samples (2-3 mg) were distributed on conducting carbon tape islands (ca. 1 $cm^2$) on a gold sample holder, with a spacing of >0.3 cm to avoid cross contamination (samples were distributed on the carbon tape before placing them on the sample holder). All samples were measured at room temperature and under UHV conditions ($10^{-9}$ mbar). After background subtraction, the spectra have been normalized to the intensity of the most intense feature of the Ti $L_3$-edge. The photon energy has been calibrated using the sharp 2p-to-3d transition in the rutile $TiO_2$ powder at 457.2 eV.[8]

## SB.7 Zn K-edge XANES Characterization of ZnTi-MWW L and ZnTi-MWW H

Zn K-edge XANES of ZnTi-MWW L and ZnTi-MWW H spectra were recorded at the European Synchrotron Radiation Facility (Grenoble, France). The X-ray beam was monochromatized using a liquid nitrogen cooled Si(111) monochromator. The energy was calibrated at 9659 eV using the first inflection point of the Zn K-edge of

a zinc metal foil standard material. The XAS Zn K-edge measurements were carried out with a fluorescent scheme of detection using a silicon drift diode with associated digital electronics. ZnTi-MWW L and ZnTi-MWW H were measured in sealed quartz capillaries (dehydrated overnight at 400 °C).
The Zn K-edge XANES reference spectra of Zn@$SiO_2$, $ZnTiO_3$, ZnO were measured as previously reported.[4]

# SC Catalytic Performance

## SC.1 (Zn)Ti-MWW Catalyzed $H_2O_2$ Decomposition Rate

The hydrogen peroxide decomposition reaction was studied to quantitatively determine the effect of different catalysts (here: (Zn)Ti-MWW) on the decomposition rate constant of $H_2O_2$, as previously described in Reference 2.

### SC.1.1 Experimental Procedure

In a clean 50 ml flask with a magnetic stirring bar and a thermometer, 30 g of deionized water and 6.5 g of a 40 weight-% hydrogen peroxide solution were placed at room temperature (22 °C). At this point a $t = 0$ aliquot of ca. 0.3 ml was taken with a pipette. The flask was lightly stoppered and then immersed in a previously equilibrated thermostating bath set to 80 °C. To obtain reproducible results it was important to use always the same amount of catalyst and to control the temperature to better than ±1 °C during the experiment. As soon as the hydrogen peroxide solution was in thermal equilibrium with the thermostating bath, 500 mg (± 1 mg) of the catalyst were added. The suspension was stirred and samples of the supernatant liquid were then taken at regular intervals. The aliquot should be taken with a 1 ml syringe with a one-way filter Millipore Millex-HV SLHV 013 NL or equivalent. First 0.6 ml of solution were sucked into the syringe through the filter. Then 0.3 ml of the solution were back-flushed through the filter into the flask. This was necessary in order to minimize loss of catalyst. The remaining 0.3 ml in the syringe were then used for the peroxide determination. The interval between aliquots was usually between 30 and 60 min depending on the catalyst activity. The experiment was finished after 7 hours.

### SC.1.2 Analytics

The aliquots were analysed for $H_2O_2$ content by using a standard cerimetric titration. It was advisable to analyse the aliquots as soon as possible after they are collected. In order to ensure a good precision, the amount of titrating solution used should be at least 5 ml. If necessary, a larger amount of aliquot had to be weighed in.

### SC.1.3 Data Analysis

The natural logarithm of the $H_2O_2$ concentration was plotted against time. Using least squares methods, the slope is extracted. This slope is the pseudo-first order decomposition rate constant of $H_2O_2$ in the presence of the catalyst (in $h^{-1}$). The error of $k_{80}$ is in the order of 0.05 $h^{-1}$.

#### SC.1.3.1 Ti-MWW L

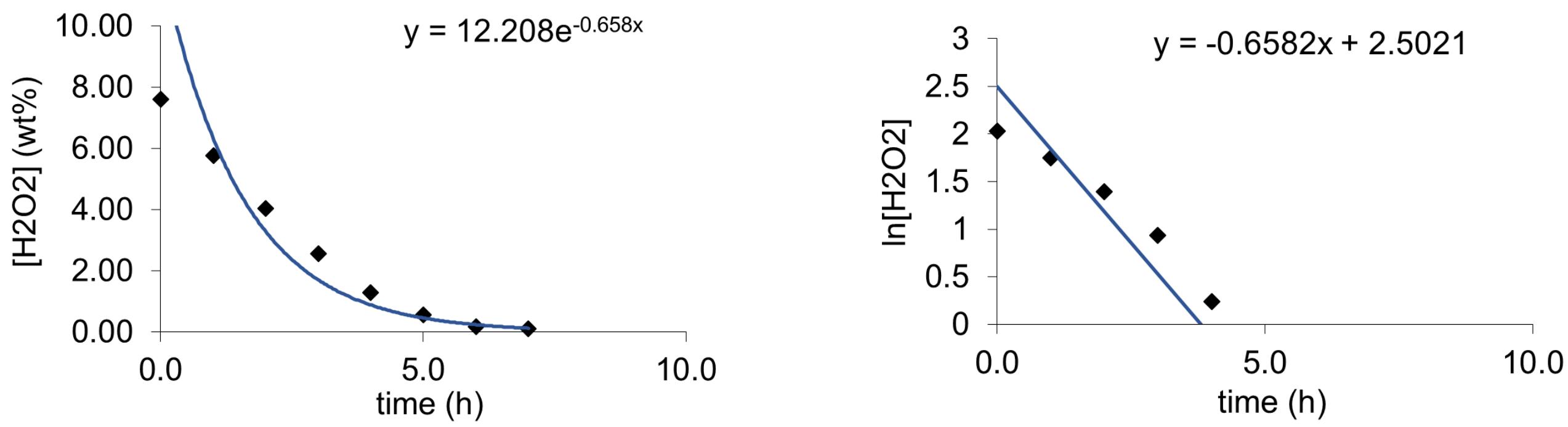


**Figure S12:** Decomposition rate constant ($k_{80} = 0.66$ $h^{-1}$) as determined for Ti-MWW L.

### SC.1.3.2 ZnTi-MWW L

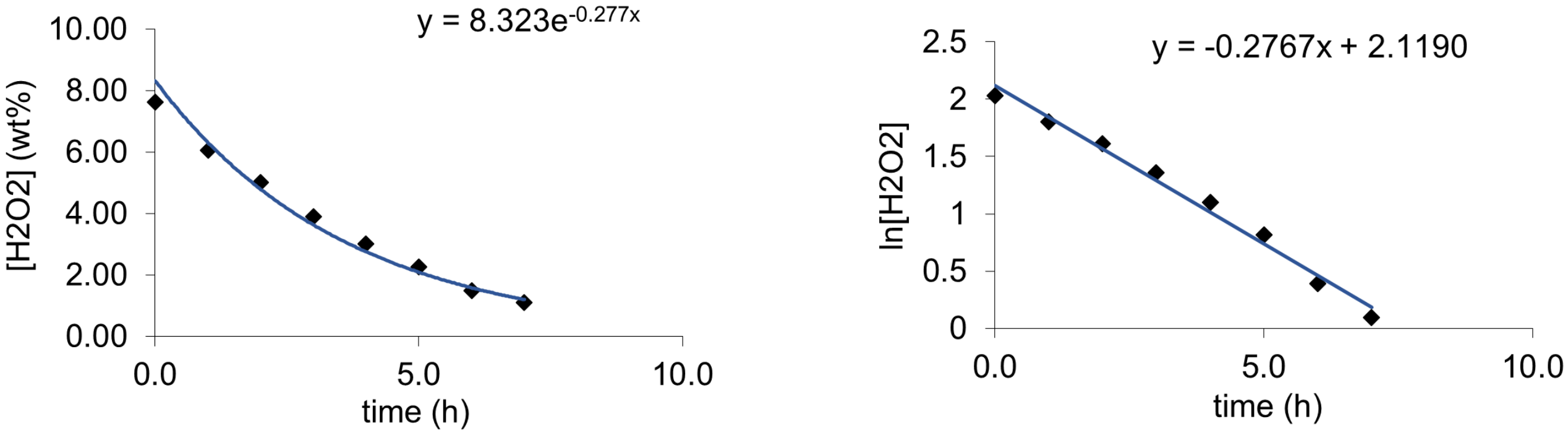


**Figure S13:** Decomposition rate constant ($k_{80} = 0.28\ h^{-1}$) as determined for ZnTi-MWW L.

### SC.1.3.3 Ti-MWW H

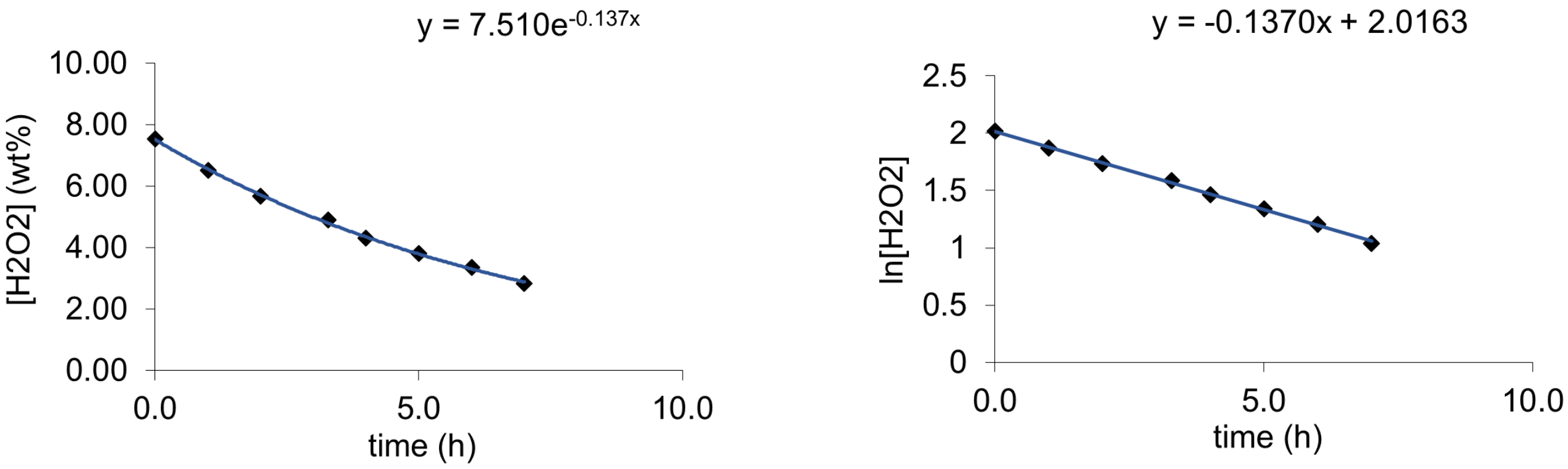


**Figure S14:** Decomposition rate constant ($k_{80} = 0.14\ h^{-1}$) as determined for Ti-MWW H. The $H_2O_2$ decomposition rate constant was already previously reported in Reference 2.

### SC.1.3.4 ZnTi-MWW H

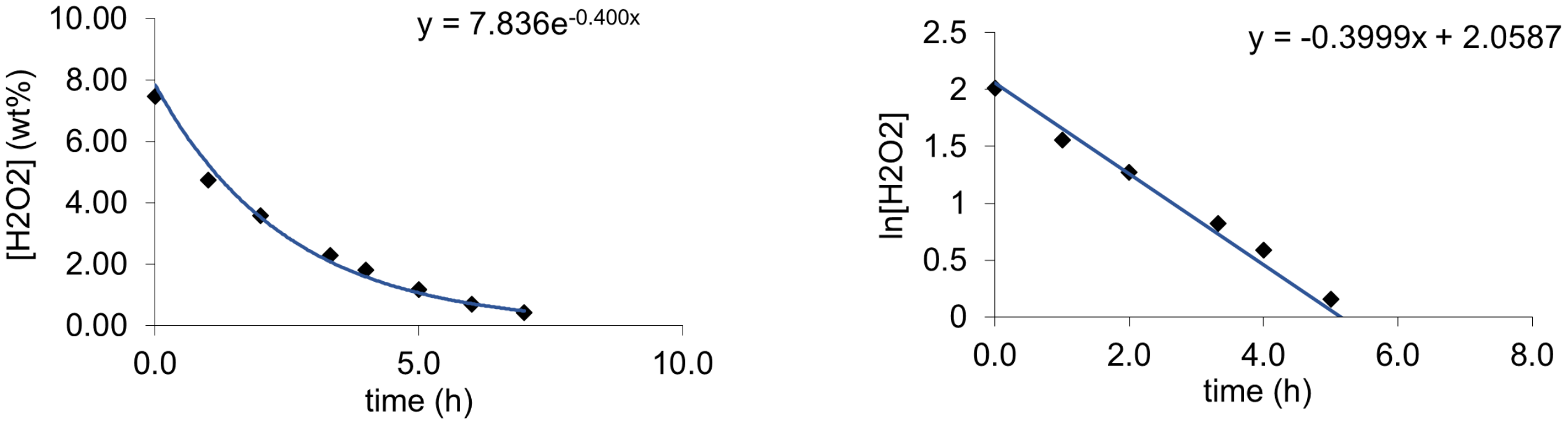


**Figure S15:** Decomposition rate constant ($k_{80} = 0.40\ h^{-1}$) as determined for ZnTi-MWW H.

### SC.1.3.5 ZnSi-MWW

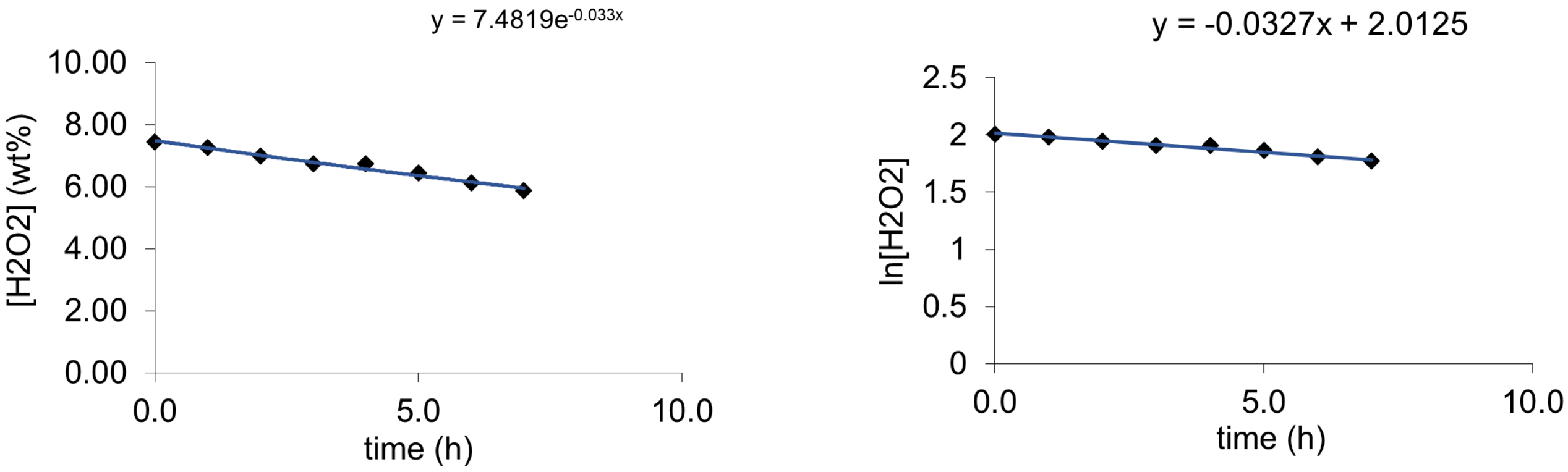


**Figure S16:** Decomposition rate constant ($k_{80} = 0.03\ h^{-1}$) as determined for ZnSi-MWW.

### SC.1.3.6 $ZnTiO_3$

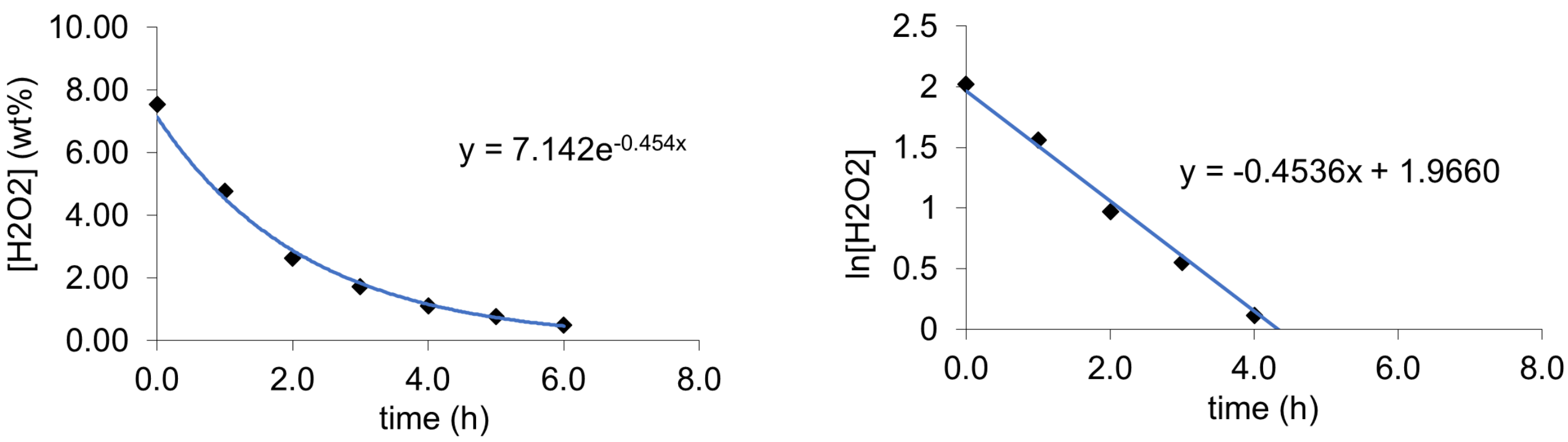


**Figure S17:** Decomposition rate constant ($k_{80} = 0.45\ h^{-1}$) as determined for $ZnTiO_3$.

## SC.2 (Zn)Ti-MWW Catalyzed Ammoximation Reaction

In a crimp-capped glass vial (20 mL) equipped with a magnetic stir bar 30 mg of the respective catalyst were suspended in a mixture of 6.00 g of a water/acetone mixture (13.7 wt.-% acetone) and 1.12 g of 25 wt.-% aqueous $NH_3$-solution. Then, 0.93 g of a 30 wt.-% aqueous $H_2O_2$ solution was added, the vial was transferred to a preheated aluminium heat block, and the mixture was stirred for 2 hours at 70 °C. Afterwards, the vial was removed from the heat block and chilled using an ice bath. Ca. 2 mL of the mixture was removed from the vial using a syringe and transferred into a GC-vial through a cellulose filter (0.20 µm). 1,4-Dioxane was added as internal standard and the sample was analyzed using GC-FID (column: Rtx-5 Amine, length = 30 m, inner diameter = 0.32 mm, film thickness = 1.5 µm, carrier gas: $N_2$, flow: 0.8 mL/min., split: 50:1, sequence: 40 °C (5 min.) – 4 °C/min. – 250 °C (0 min.)). In general, the ammoximation conversion was determined by running duplicates and the maximum deviation from the mean is 1.5%.

# SD $^{17}O$ NMR Quantification of Peroxo Formation and Stability

## SD.1 Experimental Details $^{17}O$ NMR Spectroscopy

The solid-state $^{17}O$ NMR spectra were all acquired on a Bruker Avance III 600 MHz (14.1 T) DNP NMR spectrometer equipped with a low-temperature 3.2 mm triple-resonance MAS cryo-probe operating at Larmor frequencies of 599.900 and 81.3414 MHz for $^1H$ and $^{17}O$, respectively. (Zn)Ti-MWW samples were impregnated with 1 equiv. of $H_2{}^{17}O_2$ with respect to Ti using an aq. solution of $H_2{}^{17}O_2$. The aq. solution of $H_2{}^{17}O_2$ was synthesized according to literature reports.[2,9] $ZnTiO_3$ and ZnO nanoparticles were impregnated using an aq. solution of $H_2{}^{17}O_2$ resulting in a surface coverage of 4 $H_2O_2/nm^2$. Each sample was packed into a sapphire MAS NMR rotor with a zirconia cap, transferred to the NMR spectrometer and rapidly inserted into the pre-cooled (100 K) MAS NMR probe head. Static spectra were recorded using a wideband WURST-CPMG (WCPMG) sequence[10,11] with a 50 µs, 0.5 MHz and shape 80 WURST pulse. WURST-CPMG spectra were acquired with a low-to-high frequency sweep. SPINAL64 with 100 kHz radio frequency was used for $^1H$ decoupling. The recycle delay was set to 0.7 s. $^{17}O$ NMR chemical shifts were referenced externally with respect to $H_2O$ at 0 ppm (at room temperature).[12] For all WCPMG measurements, the spectra were obtained by coadding the individual echoes of the WCPMG-echo train in the time domain prior to Fourier transform and magnitude calculation. The resulting full echoes were apodized using a Hamming window function with $\alpha = 0.54$. Simulations and deconvolution of the recorded spectroscopic signatures were performed using the DMFit software.[13] The recorded spectroscopic signatures were deconvoluted similar as previously reported and established in Reference 2, the parameters used for fitting are listed in Table S2. Additionally, the NMR parameters used for fitting the $^{17}O$ species formed on $ZnTiO_3$ and ZnO (transforms to $ZnO_2$) are listed in Table S3.

**Table S2:** $^{17}O$ NMR parameters used for fitting (deconvolution) of the impregnated (1.6 M $H_2{}^{17}O_2$) (Zn)Ti-MWW samples. NMR parameters are given in the Herzfeld-Berger notation (n.d. = not determined).

| Species | $\delta_{iso}$ (ppm) | $\Omega$ (ppm) | $\kappa$ | $C_Q$ (MHz) | $\eta_Q$ | $\varphi$ | $\chi$ | $\psi$ |
|---|---|---|---|---|---|---|---|---|
| $H_2O$ | 0 | n.d. | n.d. | 7.1 | 0.71 | n.d. | n.d. | n.d. |
| Si−O−Si | 25 | n.d. | n.d. | 6.1 | 0.10 | n.d. | n.d. | n.d. |
| $H_2O_2$ | 195 | 368 | 0.26 | 15.2 | 0.78 | 90 | 0 | 0 |
| Ti $\mu_2$-$\eta^2$:$\eta^2$-peroxo | 425 | 179 | -0.35 | 16.0 | 0.24 | 90 | 0 | 0 |

**Table S3:** $^{17}O$ NMR parameters used for fitting the peroxo formed upon impregnation (1.1 M $H_2{}^{17}O_2$) of $ZnTiO_3$ and ZnO (transforms to $ZnO_2$). NMR parameters are given in the Herzfeld-Berger notation (n.d. = not determined).

| Compound | $\delta_{iso}$ (ppm) | $\Omega$ (ppm) | $\kappa$ | $C_Q$ (MHz) | $\eta_Q$ | $\varphi$ | $\chi$ | $\psi$ |
|---|---|---|---|---|---|---|---|---|
| $ZnTiO_3$ | 405 | 179 | -0.35 | 16.4 | 0.24 | 90 | 0 | 0 |
| $ZnO_2$ | 230 | n.d. | n.d. | 18.1 | 0.00 | n.d. | n.d. | n.d. |

## SD.2 $\mu_2$-$\eta^2$:$\eta^2$-Peroxo Formation and Stability on (Zn)Ti-MWW L and (Zn)Ti-MWW H

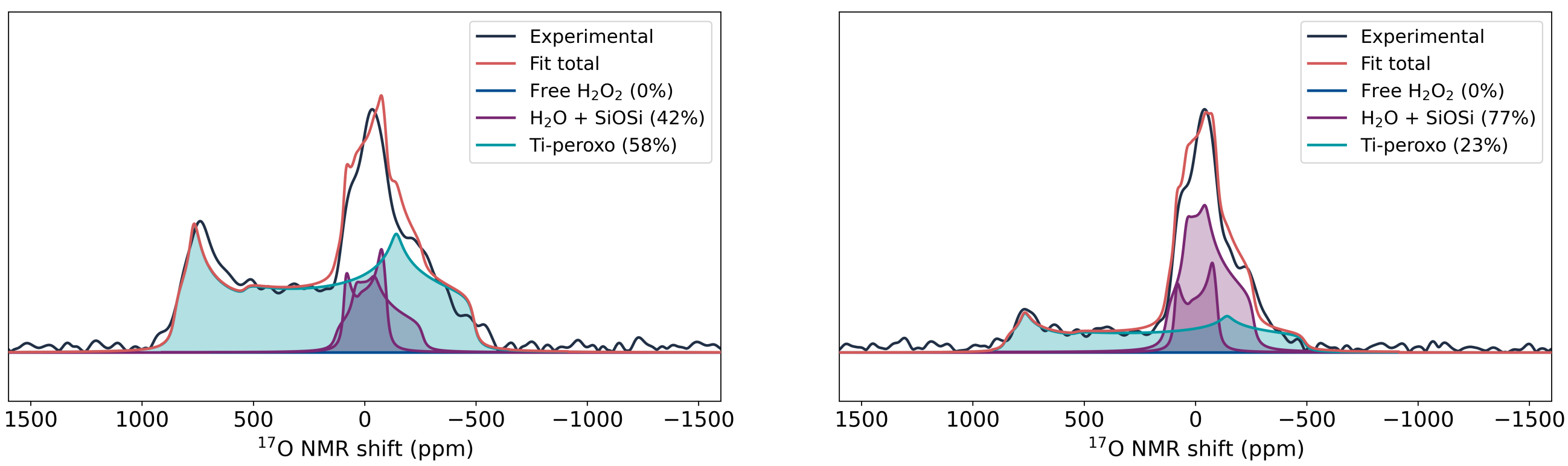


**Figure S18:** $^{17}O$ WURST CPMG NMR spectra of aqueous 1.6 M $H_2{}^{17}O_2$ on Ti-MWW L (left: 15 min after impregnation (number of scans: 52672), right: after prolonged (2 h) contact time (number of scans: 45000)), as well as the respective deconvolutions (fit).

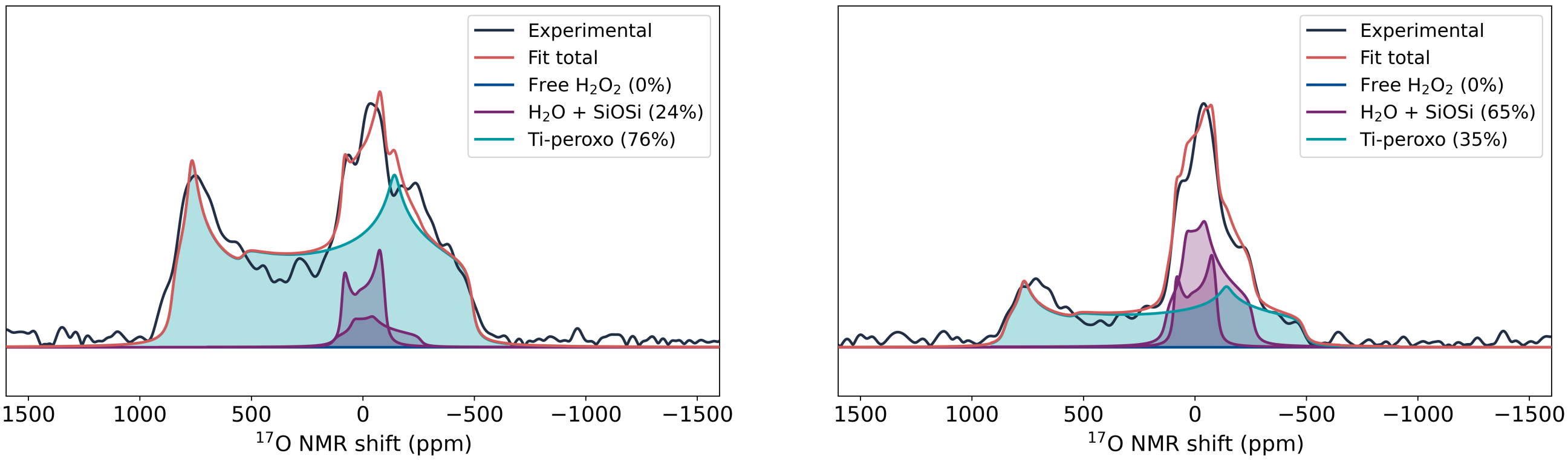


**Figure S19:** $^{17}O$ WURST CPMG NMR spectra of aqueous 1.6 M $H_2{}^{17}O_2$ on ZnTi-MWW L (left: 15 min after impregnation (number of scans: 26384), right: after prolonged (2 h) contact time (number of scans: 38416)), as well as the respective deconvolutions (fit).

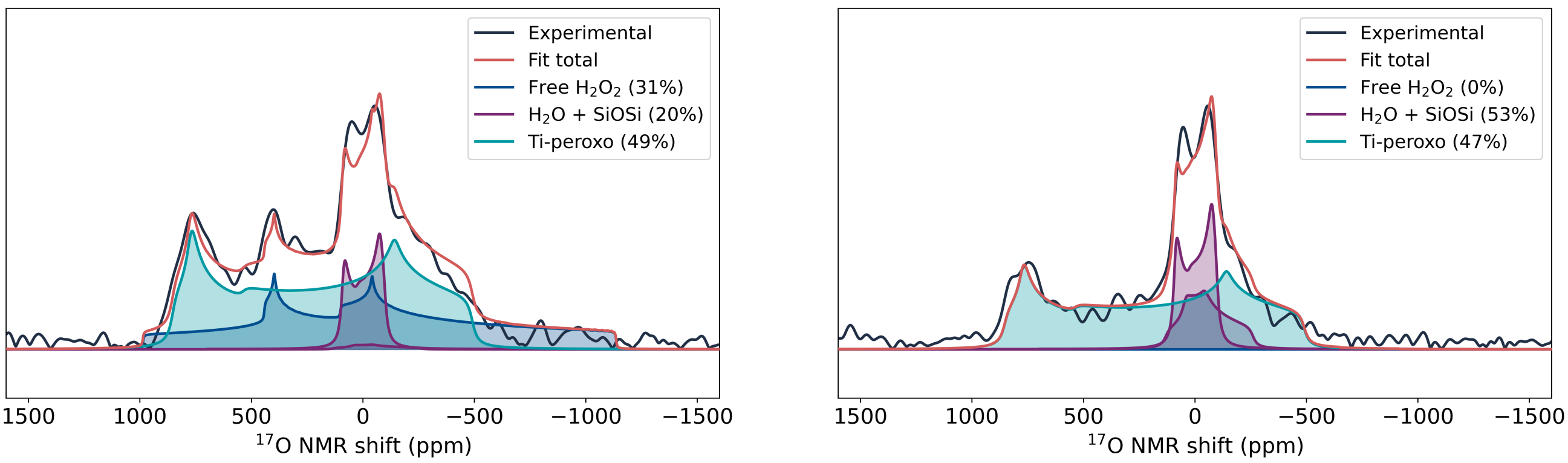


**Figure S20:** $^{17}O$ WURST CPMG NMR spectra of aqueous 1.6 M $H_2{}^{17}O_2$ on Ti-MWW H previously reported in Reference 2 (left: 15 min after impregnation (number of scans: 78304), right: after prolonged (2 h) contact time (number of scans: 63848)), as well as the respective deconvolutions (fit).

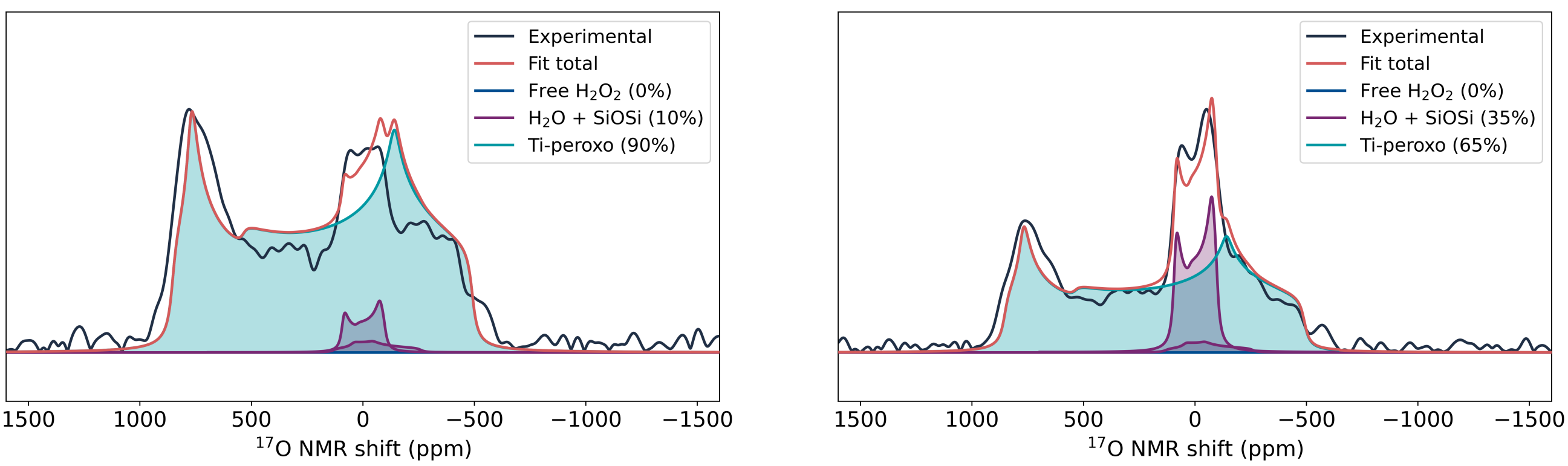


**Figure S21:** $^{17}O$ WURST CPMG NMR spectra of aqueous 1.6 M $H_2{}^{17}O_2$ on ZnTi-MWW H (left: 15 min after impregnation (number of scans: 12232), right: after prolonged (2 h) contact time (number of scans: 53528)), as well as the respective deconvolutions (fit).

## SD.3 Investigated Peroxo Formation on ZnSi-MWW

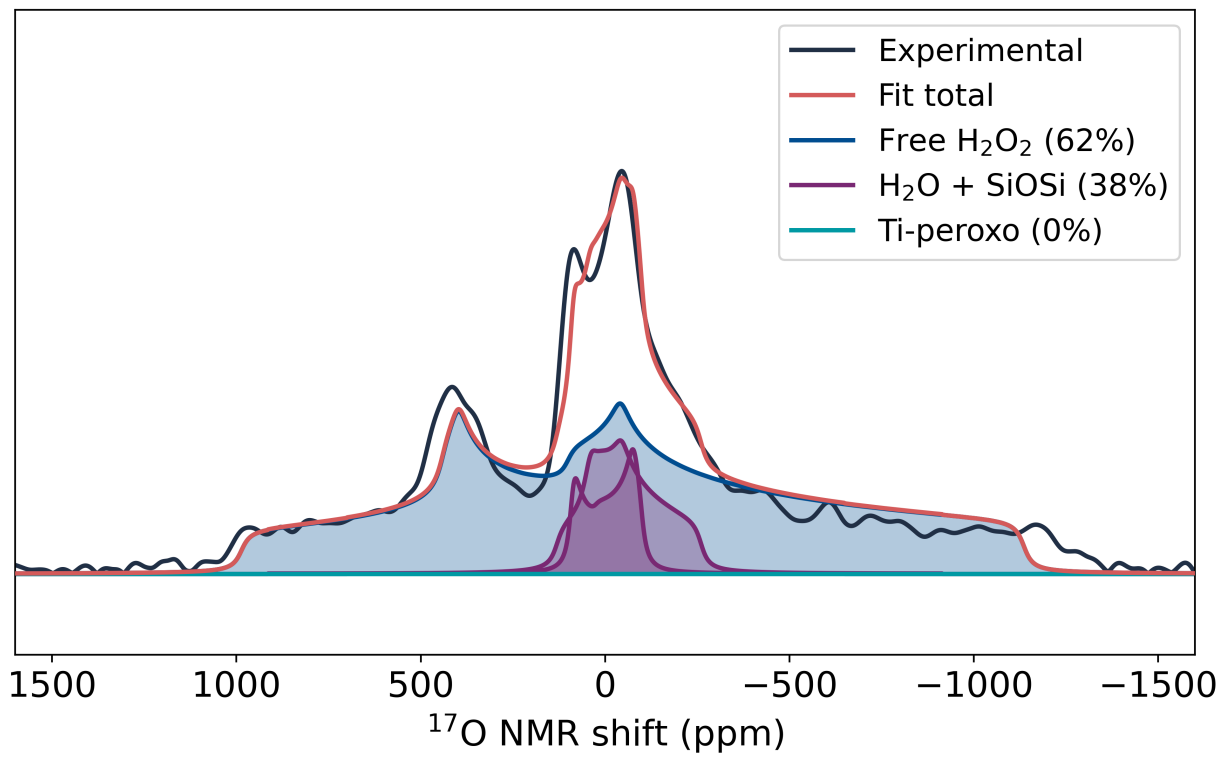


**Figure S22:** $^{17}O$ WURST CPMG NMR spectrum of aqueous 1.1 M $H_2{}^{17}O_2$ on ZnSi-MWW (15 min after impregnation (number of scans: 76408)), as well as the respective deconvolution (fit).

## SD.4 Peroxo Formation and Stability on $ZnTiO_3$ and ZnO

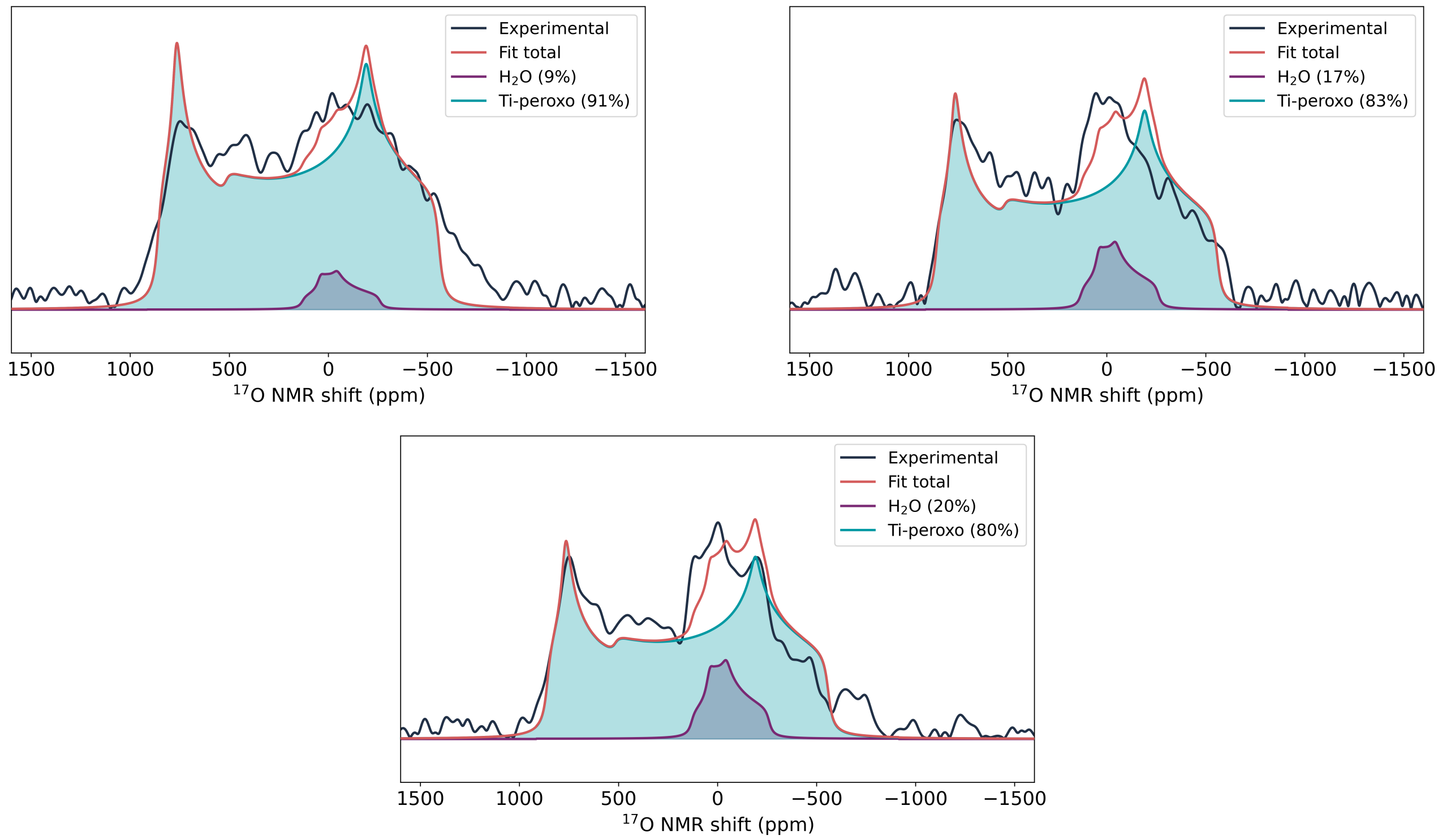


**Figure S23:** $^{17}O$ WURST CPMG NMR spectra of aqueous 1.1 M $H_2{}^{17}O_2$ on $ZnTiO_3$ (top left: 15 min after impregnation (number of scans: 57264), top right: after prolonged (6 h) contact time (number of scans: 65040), bottom: after prolonged (12 h) contact time (number of scans: 117920)), as well as the respective deconvolutions (fit).

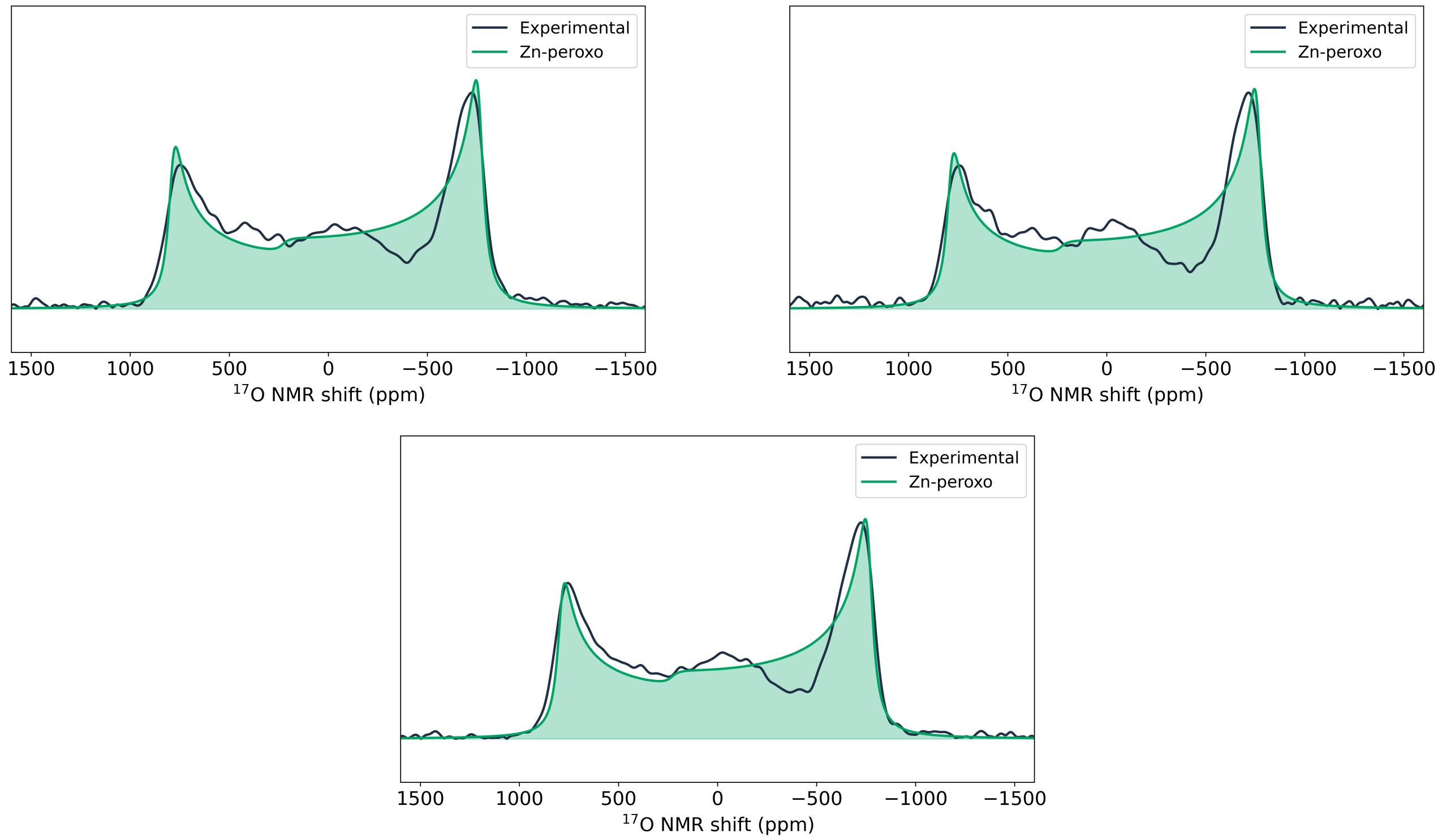


**Figure S24:** $^{17}$O WURST CPMG NMR spectra of aqueous 1.1 M $H_2{}^{17}O_2$ on ZnO (top left: 15 min after impregnation (number of scans: 45672), top right: after prolonged (12 h) contact time (number of scans: 42160), bottom: after prolonged (24 h) contact time (number of scans: 89160)), as well as the respective deconvolutions (fit).

# SE $^{15}$N Low Temperature NMR upon Pyridine Adsorption

## SE.1 Adsorption of $^{15}$N-pyridine

For all pyridine adsorption studies $^{15}$N-pyrdine (99% $^{15}$N isotopic enrichment) purchased from CortecNet Corp. was used. It was dried at 60°C (72 hours) over $CaH_2$ under Ar atmosphere and degassed using three consecutive freeze-pump-thaw cycles before every adsorption experiment. Typically, 100-150 mg of each sample was evacuated overnight at ca. 400°C under high vacuum conditions prior to adsorption of pyridine. Pyridine was adsorbed at the samples via direct saturation for 5 min, followed by evacuation of physisorbed pyridine for 45 min under high vacuum ($10^{-5}$ mbar).

## SE.2 Experimental Details $^{15}$N NMR Spectroscopy

Low-temperature (100 K) solid-state $^{1}$H-$^{15}$N Cross-Polarization Magic Angle Spinning (CPMAS) NMR spectra were acquired on a Bruker Avance III 600 MHz (14.1 T) DNP NMR spectrometer equipped with a low-temperature 3.2 mm double-resonance MAS DNP probe operating at Larmor frequencies of 600.020 and 60.812 MHz for $^{1}$H and $^{15}$N, respectively.
Samples where pyridine was adsorbed on the dehydrated material were subsequently prepared for solid-state NMR analyses under dry and oxygen-free argon atmosphere in an MBraun glovebox equipped with a purifier unit (<0.5 ppm $O_2$, <0.5 ppm $H_2O$). 3.2 mm zirconia rotors closed with a VESPEL drive cap (marked with three layers of permanent marker) were used to avoid oxygen and moisture contamination. The NMR rotors were introduced into the glovebox while hot, and all plastic NMR rotor caps and packing tools were introduced into the glovebox overnight under vacuum to avoid contamination with air or water. After sample packing, the NMR rotors and caps were placed in argon vials with screw-cap tops and transferred into glove bags filled with dry synthetic air, where the rotors where closed. The presence of oxygen in the NMR rotor reduces the measurement time by an order of magnitude due to dramatic shortening of the relaxation times. The NMR rotors were transferred to the NMR spectrometer, rapidly inserted into the MAS NMR probehead, and spun with dry $N_2$ gas. The MAS NMR probehead was pre-cooled to 100 K (measured in the gas stream in the MAS spinning housing) prior to pneumatic insertion of the MAS NMR rotors.
The $^{1}$H-$^{15}$N CPMAS spectra of the $^{15}$N-pyridine adsorbed samples were acquired with $^{1}$H-$^{15}$N contact times of 3 ms, at 8 kHz MAS, and with 100 kHz SPINAL-64 $^{1}$H heteronuclear decoupling during the acquisition periods. The recycle delay was set to 1 s and the number of scans were typically between 5k and 65k (some experiments have larger number of scans than required due to overnight runtimes). The signals were referenced with respect to liquid $NH_3$ at 0 ppm via the secondary reference glycine at 33.4 ppm at room temperature.[14] The processing and fitting of $^{15}$N NMR data was conducted using TopSpin (version 3.6.5, Bruker BioSpin GmbH) and DMFit[13] software packages. For processing of the acquired $^{15}$N NMR spectra, apodization was performed using an exponential line broadening of 32 Hz. The NMR parameters used for fitting the $^{15}$N species are listed in Tables S4 - S8.

## SE.3 Recorded $^{15}$N NMR LT Spectra and Deconvoluted Signals

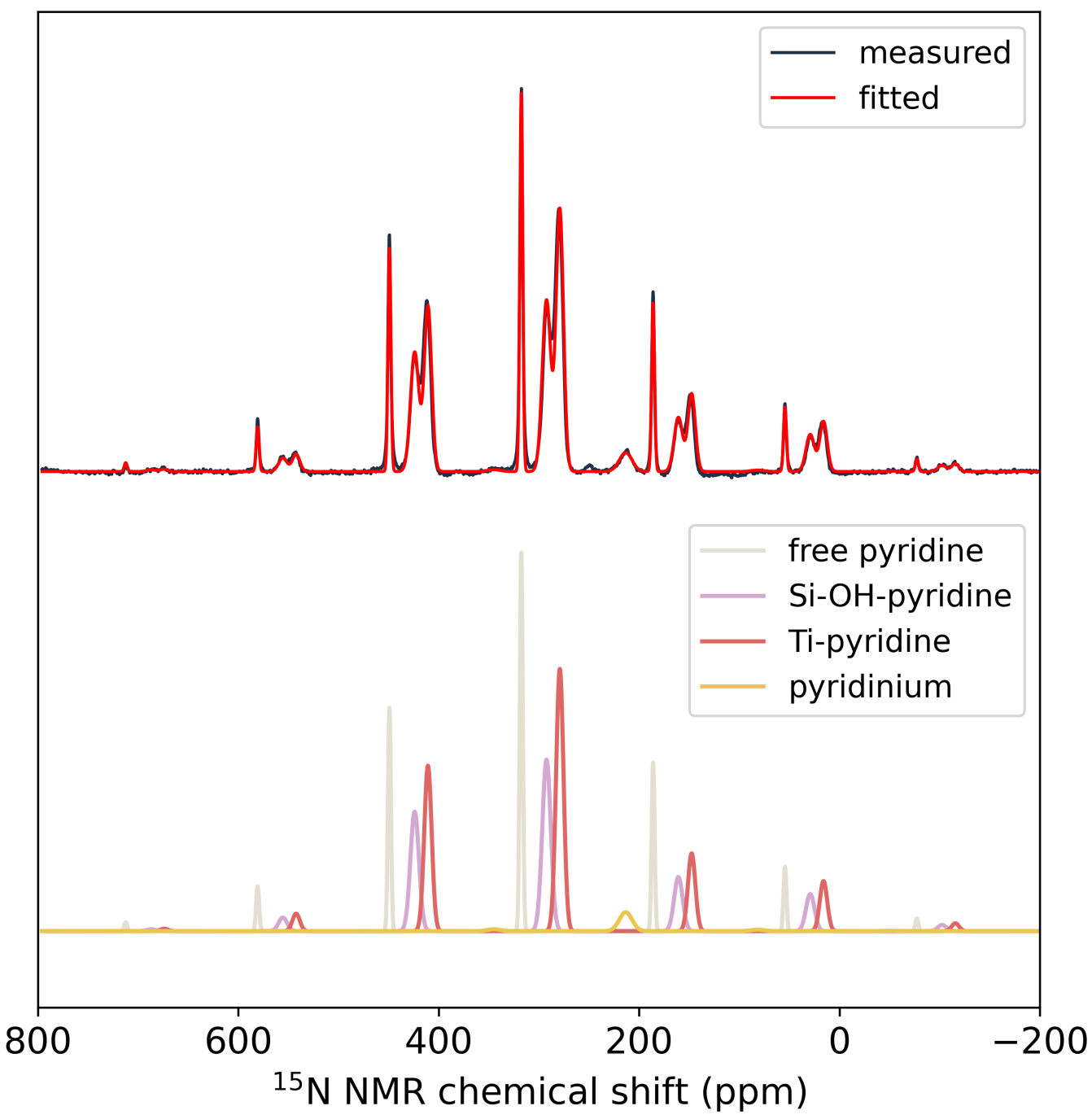


**Figure S25:** $^{1}$H-$^{15}$N CPMAS NMR spectrum of $^{15}$N-pyridine adsorbed on Ti-MWW L with 1.8 wt.% Ti. a) Measured (gray) and fitted spectrum (red). b) Deconvoluted spectrum (beige: free pyridine, purple: pyridine bound to silanol, red: pyridine bound to Lewis acidic Ti sites, yellow: pyridinium). Number of scans was set to 15704.

**Table S4:** $^{15}$N NMR parameters obtained from the $^{1}$H-$^{15}$N CPMAS NMR spectrum of $^{15}$N-pyridine adsorbed on Ti-MWW L. Integral intensity was normalized to the peak of Si-OH-pyridine. NMR parameters are given in the Herzfeld-Berger notation.

| Species | $\delta_{iso}$ (ppm) | $\Omega$ (ppm) | $\kappa$ | Integral Intensity |
|---|---|---|---|---|
| Free pyridine | 317.6 | 551 | 0.22 | 0.78 |
| Si-OH-pyridine | 292.5 | 479 | 1.00 | 1.00 |
| Ti-pyridine | 279.2 | 462 | 1.00 | 1.25 |
| pyridinium | 213.5 | 242 | 0.53 | 0.09 |

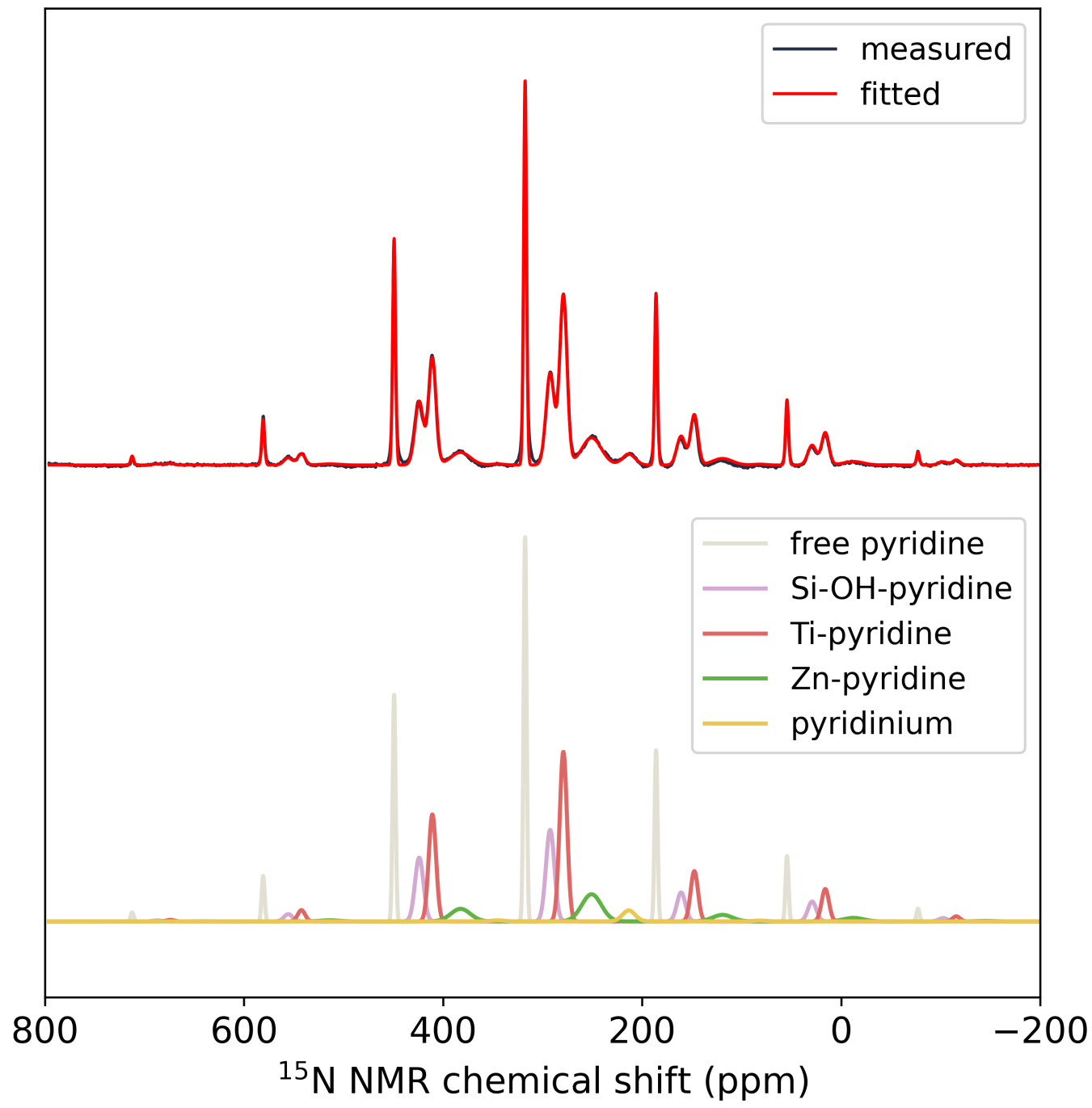


**Figure S26:** $^{1}$H-$^{15}$N CPMAS NMR spectrum of $^{15}$N-pyridine adsorbed on ZnTi-MWW L with 1.8 wt.% Ti. a) Measured (gray) and fitted spectrum (red). b) Deconvoluted spectrum (beige: free pyridine, purple: pyridine bound to silanol, red: pyridine bound to Lewis acidic Ti sites, green: pyridine bound to Lewis acidic Zn sites, yellow: pyridinium). Number of scans was set to 45000.

**Table S5:** $^{15}$N NMR parameters obtained from the $^{1}$H-$^{15}$N CPMAS NMR spectrum of $^{15}$N-pyridine adsorbed on ZnTi-MWW L. Integral intensity was normalized to the peak of Si-OH-pyridine. NMR parameters are given in the Herzfeld-Berger notation.

| Species | $\delta_{iso}$ (ppm) | $\Omega$ (ppm) | $\kappa$ | Integral Intensity |
|---|---|---|---|---|
| Free pyridine | 317.6 | 551 | 0.22 | 1.45 |
| Si-OH-pyridine | 292.5 | 479 | 1.00 | 1.00 |
| Ti-pyridine | 279.2 | 462 | 1.00 | 1.50 |
| Zn-pyridine | 250.9 | 411 | 1.00 | 0.55 |
| pyridinium | 213.5 | 242 | 0.53 | 0.09 |

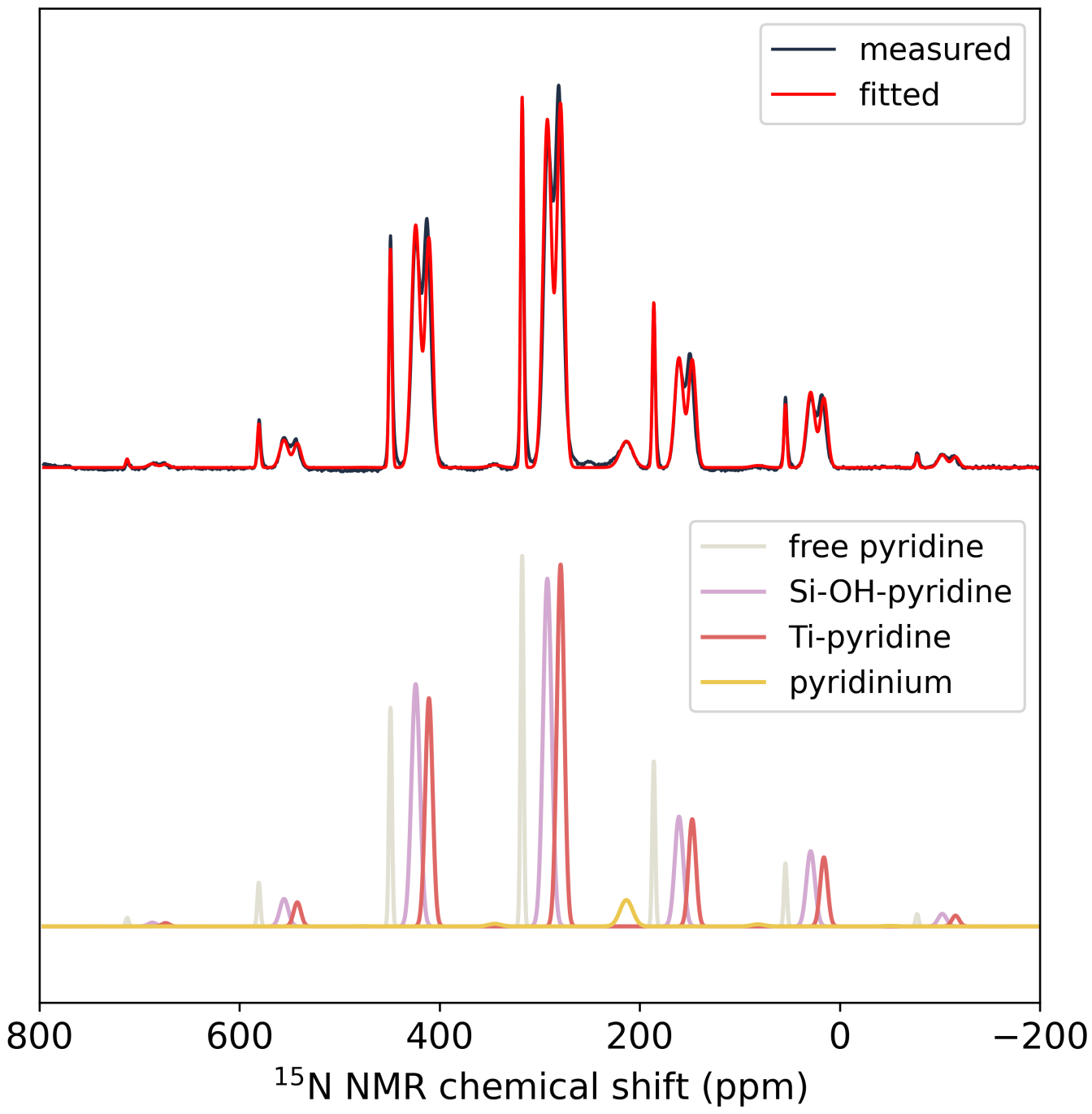


**Figure S27:** $^{1}$H-$^{15}$N CPMAS NMR spectrum of $^{15}$N-pyridine adsorbed on Ti-MWW H with 2.8 wt.% Ti. a) Measured (gray) and fitted spectrum (red). b) Deconvoluted spectrum (beige: free pyridine, purple: pyridine bound to silanol, red: pyridine bound to Lewis acidic Ti sites, yellow: pyridinium). Number of scans was set to 63576.

**Table S6:** $^{15}$N NMR parameters obtained from the $^{1}$H-$^{15}$N CPMAS NMR spectrum of $^{15}$N-pyridine adsorbed on Ti-MWW H. Integral intensity was normalized to the peak of Si-OH-pyridine. NMR parameters are given in the Herzfeld-Berger notation.

| Species | $\delta_{iso}$ (ppm) | $\Omega$ (ppm) | $\kappa$ | Integral Intensity |
|---|---|---|---|---|
| Free pyridine | 317.6 | 551 | 0.22 | 0.36 |
| Si-OH-pyridine | 292.5 | 479 | 1.00 | 1.00 |
| Ti-pyridine | 279.2 | 462 | 1.00 | 0.84 |
| pyridinium | 213.5 | 242 | 0.53 | 0.07 |

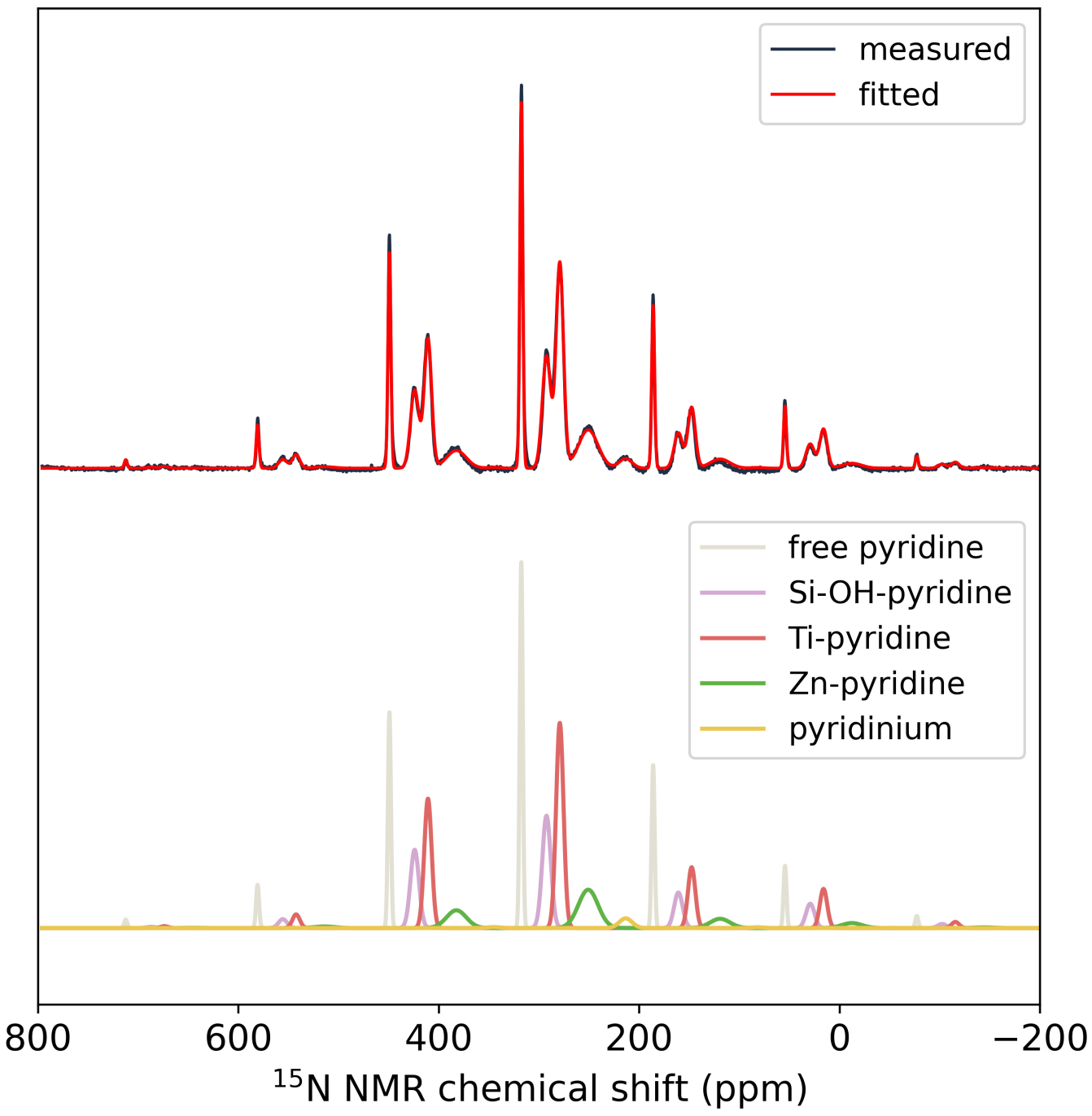


**Figure S28:** $^{1}H$-$^{15}N$ CPMAS NMR spectrum of $^{15}N$-pyridine adsorbed on ZnTi-MWW H with 2.4 wt.% Ti. a) Measured (gray) and fitted spectrum (red). b) Deconvoluted spectrum (beige: free pyridine, purple: pyridine bound to silanol, red: pyridine bound to Lewis acidic Ti sites, green: pyridine bound to Lewis acidic Zn sites, yellow: pyridinium). Number of scans was set to 17040.

**Table S7:** $^{15}N$ NMR parameters obtained from the $^{1}H$-$^{15}N$ CPMAS NMR spectrum of $^{15}N$-pyridine adsorbed on ZnTi-MWW H. Integral intensity was normalized to the peak of Si-OH-pyridine. NMR parameters are given in the Herzfeld-Berger notation.

| Species | $\delta_{iso}$ (ppm) | $\Omega$ (ppm) | $\kappa$ | Integral Intensity |
|---|---|---|---|---|
| Free pyridine | 317.6 | 551 | 0.22 | 1.13 |
| Si-OH-pyridine | 292.5 | 479 | 1.00 | 1.00 |
| Ti-pyridine | 279.2 | 462 | 1.00 | 1.52 |
| Zn-pyridine | 250.9 | 411 | 1.00 | 0.65 |
| pyridinium | 213.5 | 242 | 0.53 | 0.09 |

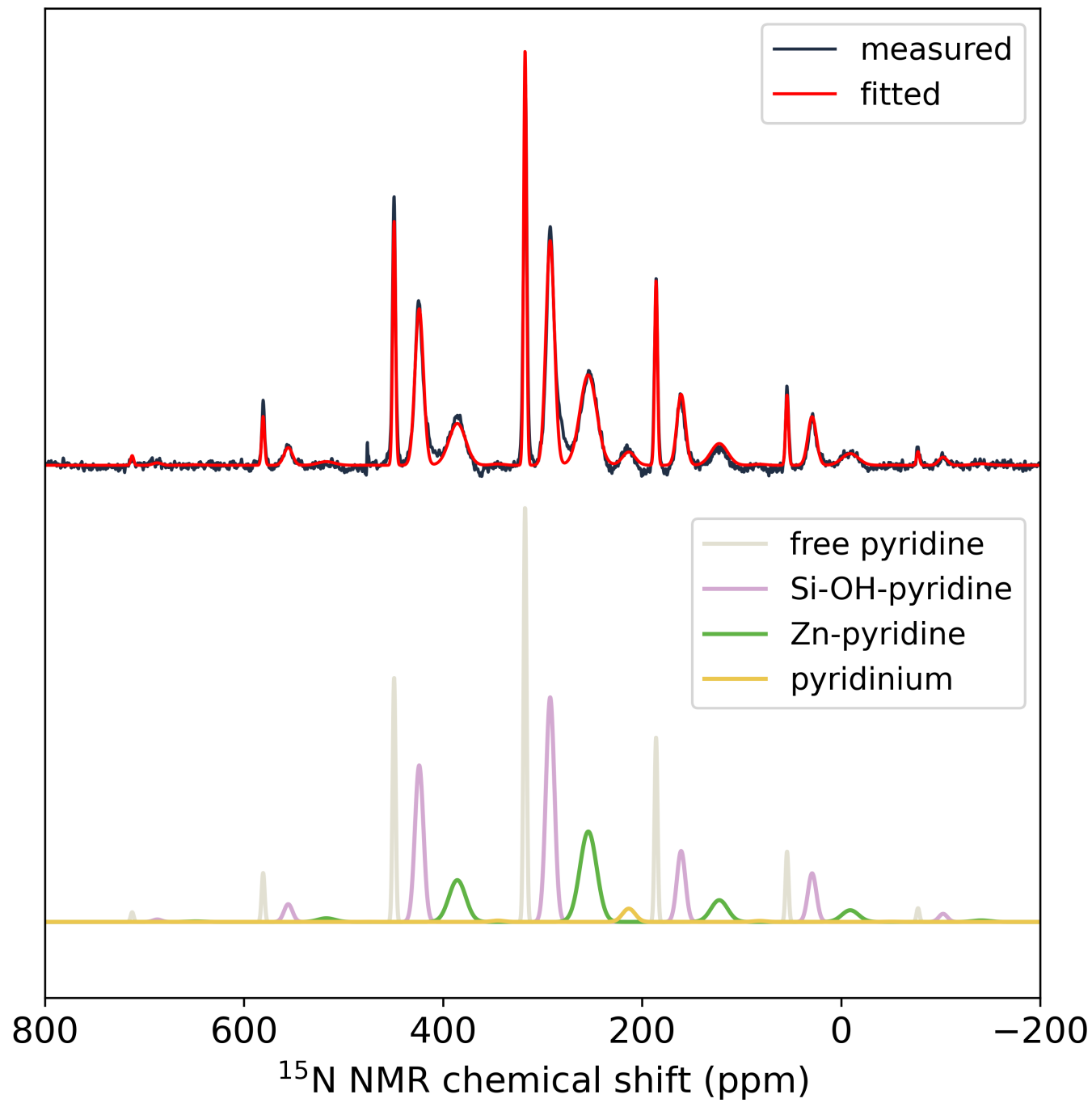


**Figure S29:** $^{1}H$-$^{15}N$ CPMAS NMR spectrum of $^{15}N$-pyridine adsorbed on ZnSi-MWW. a) Measured (gray) and fitted spectrum (red). b) Deconvoluted spectrum (beige: free pyridine, purple: pyridine bound to silanol, green: pyridine bound to Lewis acidic Zn sites, yellow: pyridinium). Number of scans was set to 5112.

**Table S8:** $^{15}N$ NMR parameters obtained from the $^{1}H$-$^{15}N$ CPMAS NMR spectrum of $^{15}N$-pyridine adsorbed on ZnSi-MWW. Integral intensity was normalized to the peak of Si-OH-pyridine. NMR parameters are given in the Herzfeld-Berger notation.

| Species | $\delta_{iso}$ (ppm) | $\Omega$ (ppm) | $\kappa$ | Integral Intensity |
|---|---|---|---|---|
| Free pyridine | 317.6 | 551 | 0.22 | 0.65 |
| Si-OH-pyridine | 292.5 | 479 | 1.00 | 1.00 |
| Zn-pyridine | 254.2 | 411 | 1.00 | 0.63 |
| pyridinium | 213.5 | 242 | 0.53 | 0.05 |

# SF Microscopy Analysis of (Zn)Ti-MWW L and (Zn)Ti-MWW H

## SF.1 Experimental Details Microscopy

Transmission electron microscopy (TEM) and scanning transmission electron microscopy (STEM) investigations were performed on a JEOL JEM-ARM300F Grand ARM "Vortex" microscope operated at 300 kV and equipped with a cold field emission gun (CFEG). Elemental analysis was carried out using EDX mapping with dual large-area SDD detectors (100 $mm^2$ active area each), providing a total solid angle of 1.6 sr. The system offered an energy resolution of $\leq$133 eV for the Mn K$\alpha$ line. Elemental maps were acquired under STEM conditions to ensure high spatial resolution and compositional sensitivity. Samples were prepared by dispersing the powder in ethanol via ultrasonication, followed by drop-casting onto copper TEM grids and drying under ambient conditions prior to insertion into the microscope.

## SF.2 Additional STEM of (Zn)Ti-MWW L and (Zn)Ti-MWW H

Additional representative STEM images of the (Zn)TiMWW samples are displayed in Figure S30.

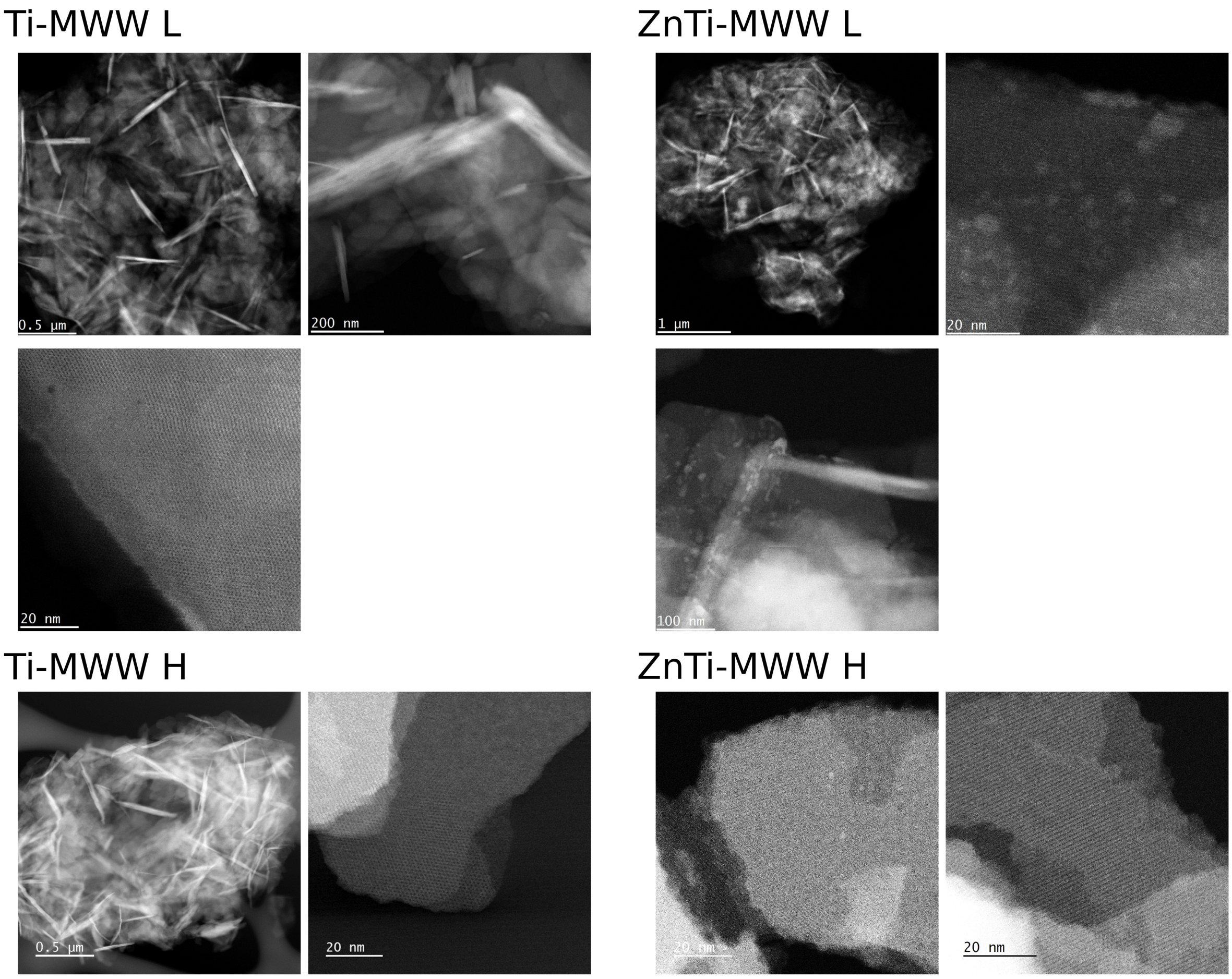


**Figure S30:** Additional STEM images of the (Zn)Ti-MWW L and (Zn)Ti-MWW H catalysts.

## SF.3 HRTEM of (Zn)Ti-MWW L

For the low Ti weight loading samples HRTEM images were acquired, depicting the regular framework structure of the (Zn)Ti-MWW L samples (see Figure S31).

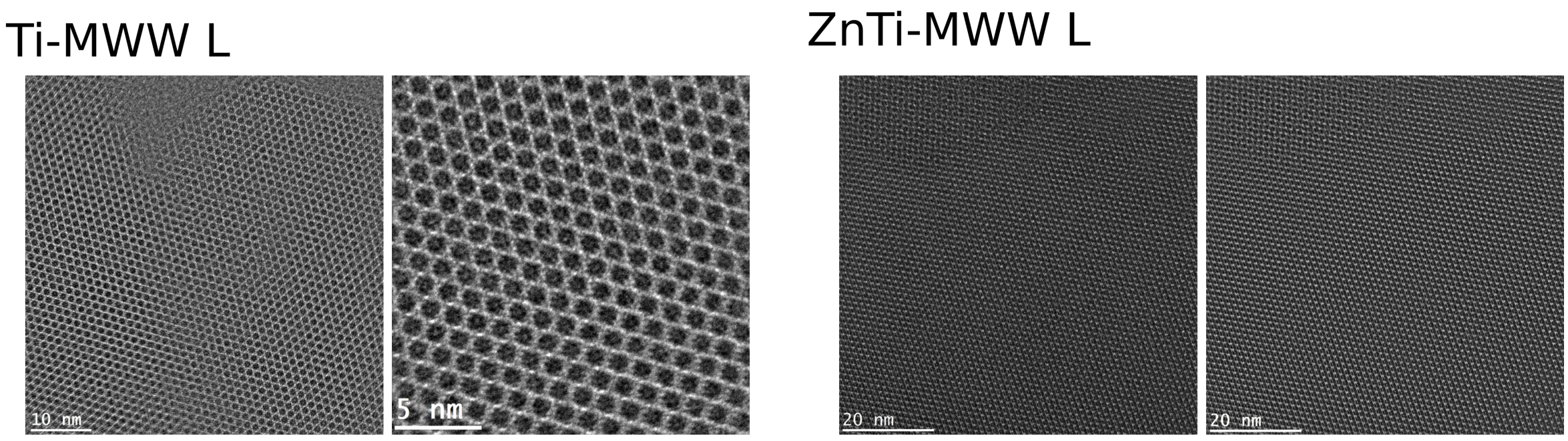


**Figure S31:** HRTEM images of the low Ti weight loading ZnTi-MWW L sample and its parent catalyst Ti-MWW L.

## SF.4 EDX Mapping of (Zn)Ti-MWW L and (Zn)Ti-MWW H

The EDX spectra of the EDX maps shown in Figure 4 are displayed in Figure S32. Additional EDX maps of the parent Ti-MWWs and the zinc treated samples are displayed in Figures S33 and S34.

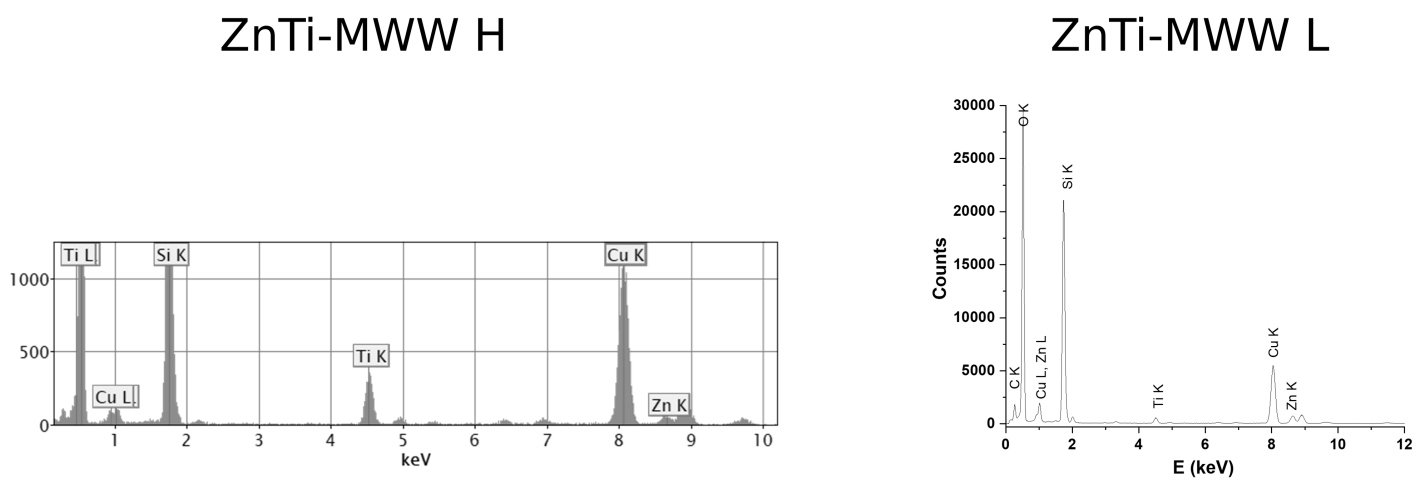


**Figure S32:** EDX spectra of the EDX maps shown in Figure 4.

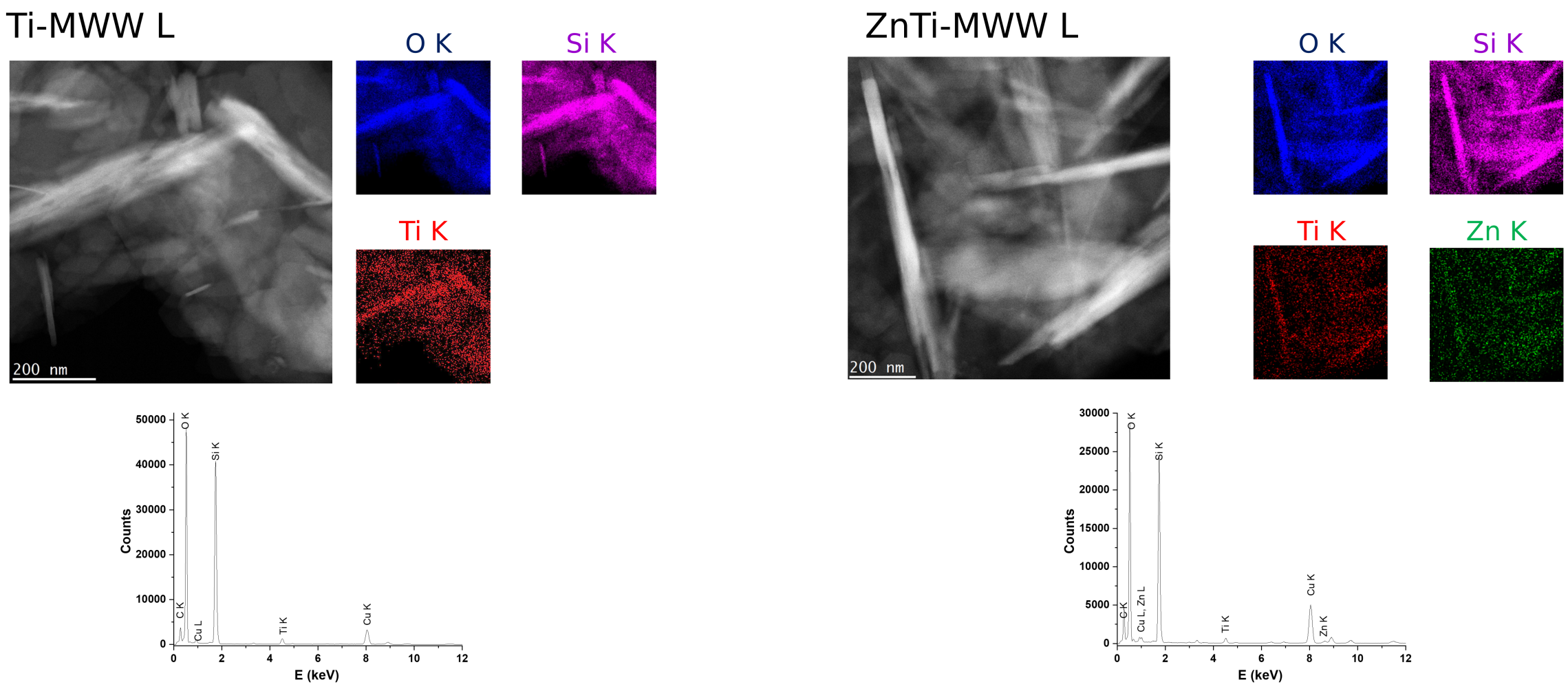


**Figure S33:** EDX maps of the low Ti weight loading ZnTi-MWW L sample and its parent catalyst Ti-MWW L.

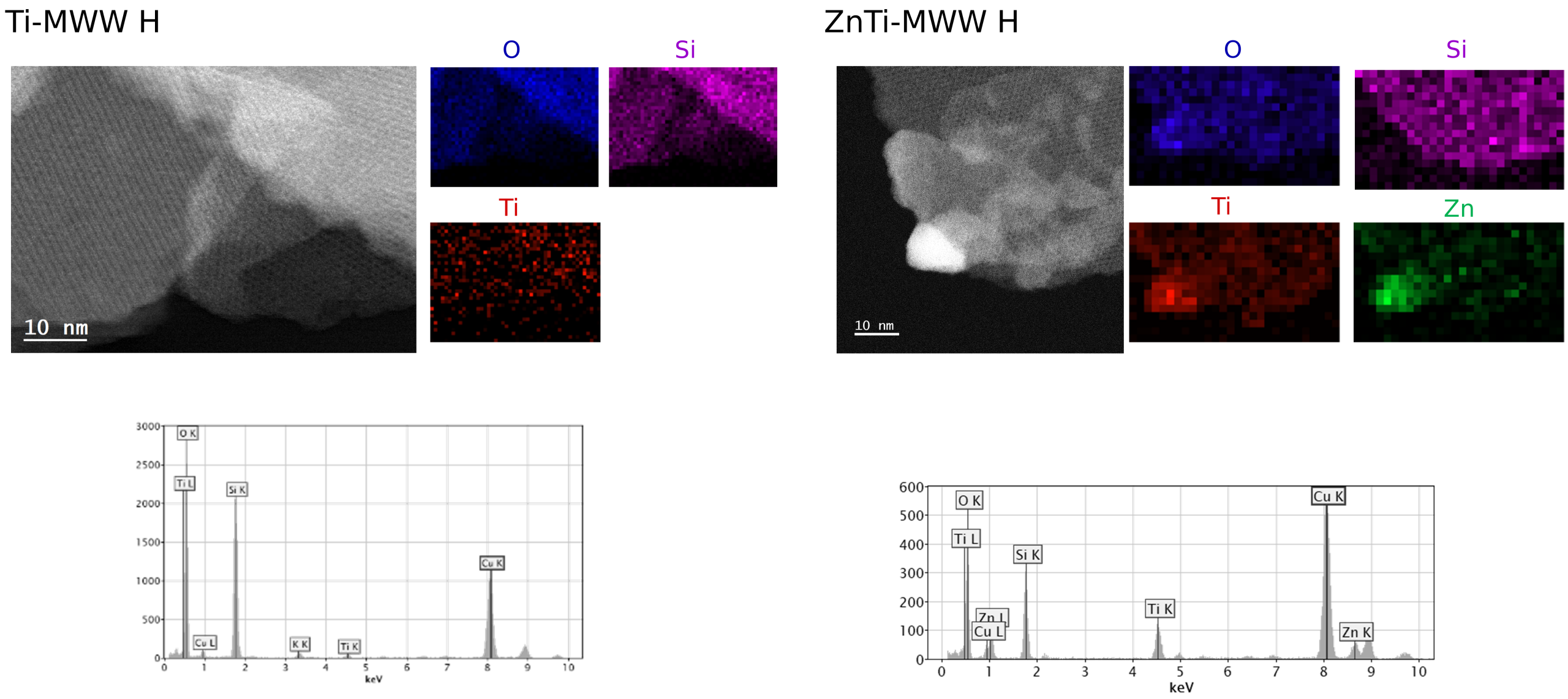


**Figure S34:** EDX maps of the high Ti weight loading ZnTi-MWW H sample and its parent catalyst Ti-MWW H.

# SG Computational Details

## SG.1 General Details for Geometry Optimization and $^{17}$O NMR Calculations

The calculations were performed on the *Euler* high-performance computing cluster of ETH Zürich.

### SG.1.1 Geometry Optimization

Geometry optimizations were performed using the $r^2$SCAN-3c[15] implementation in the ORCA 6.0.1 package,[16,17] which is based on the $r^2$SCAN[18] meta-GGA functional combined with the D4 dispersion correction[19] and the geometrical counter-poise correction,[20] employing the modified triple-$\zeta$ basis set, def2-mTZVPP. Vibrational frequency analyses of all fully relaxed structures verified minima structures (zero imaginary frequencies).

### SG.1.2 $^{17}$O NMR Parameter Calculations

The optimized structures employed in the NMR parameter calculations were obtained as described in Section SG.1.1. $^{17}$O NMR parameter calculations within the GIAO framework[21–23] employed an all-electron spin-orbit relativistic ZORA[24–27] approach, using the hybrid PBE0[28] functional and Slater-type TZ2P[29] basis sets on all elements, available in the Amsterdam Density Functional (ADF) program[30,31] (version 2023.101) integrated into the Amsterdam Modeling Suite (AMS).[32] The numerical quality was set to VeryGood and symmetry was set to NOSYM.

## SG.2 Benchmark $^{17}$O NMR Parameter Calculations

The benchmark study used a set of molecular references in close analogy to the the benchmark set used in Reference 33.

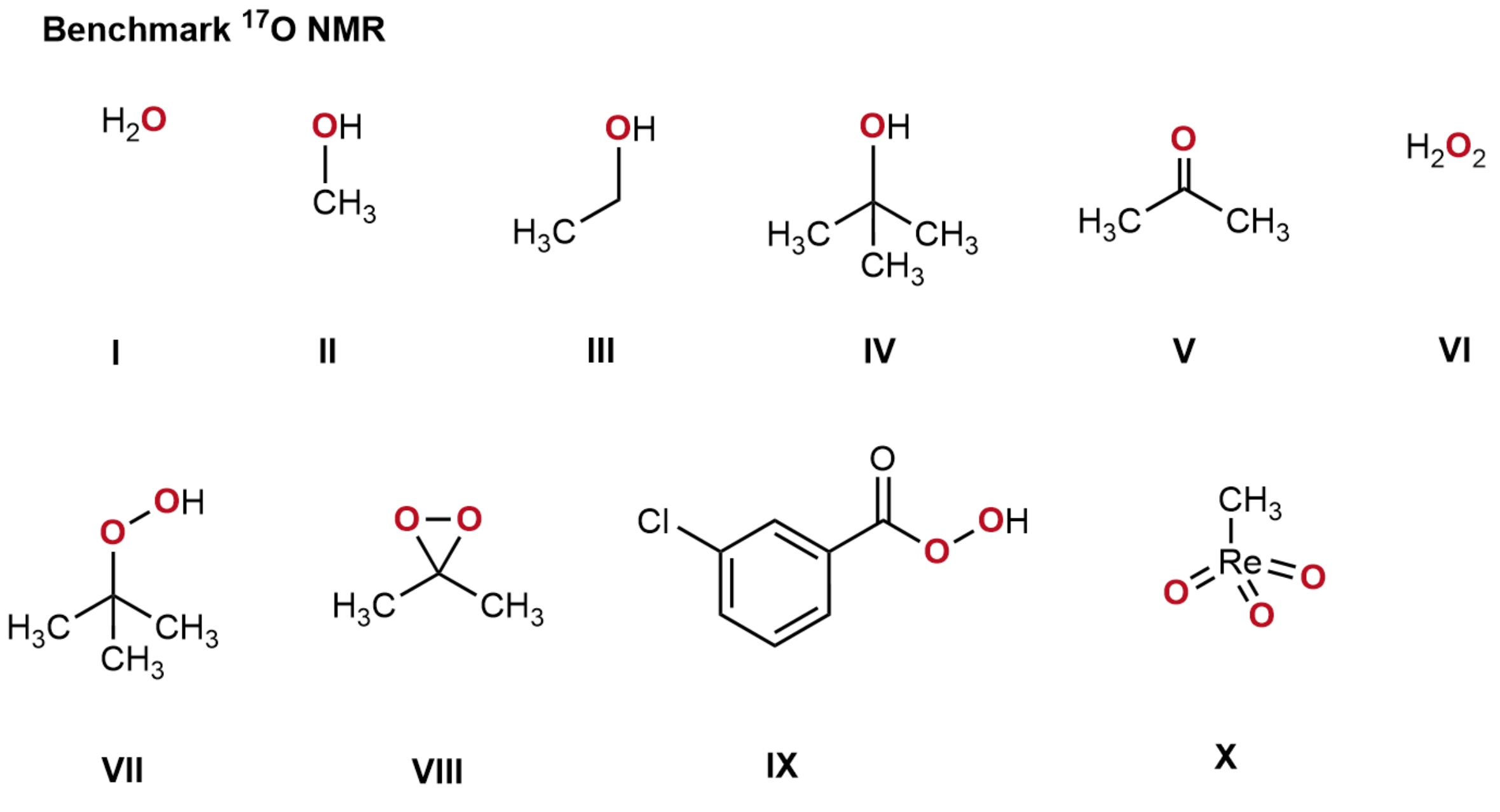


**Figure S35:** Library of oxygen-containing molecules used for the benchmark study of the $^{17}$O NMR parameter calculations.

### SG.2.1 $^{17}$O Isotropic Chemical Shift

In general, the isotropic $^{17}$O NMR chemical shifts of the computed structures $i$ ($\delta_{\text{iso, calc},i}(^{17}\text{O})$) were obtained from the calculated $^{17}$O NMR shielding values ($\sigma_{\text{iso, calc},i}(^{17}\text{O})$) via the correlation obtained from the benchmark set (Equation SG.1). The calculated isotropic $^{17}$O NMR chemical shifts values of the benchmark structures (**I** - **X**) yielded very good agreement with the experimental isotropic $^{17}$O chemical shift values reported in literature.[33–37]

$$\delta_{\text{iso, calc},i}(^{17}\text{O}) = 290.46 - 0.912 \times \sigma_{\text{iso, calc},i}(^{17}\text{O}) \qquad \text{(SG.1)}$$

**Table S9:** Summary of computed and experimental $^{17}$O NMR parameters of the isotropic chemical shift benchmark set. Calculated isotropic shielding $\sigma_{\text{iso, calc}}(^{17}\text{O})$ values obtained via the protocol described in Section SG.1.2 were used to calculate the isotropic chemical shift $\delta_{\text{iso, calc}}(^{17}\text{O})$ values via the correlation plotted in Figure S36.

| | Formula | $\sigma_{\text{iso, calc}}(^{17}\text{O})$ (ppm) | $\delta_{\text{iso, calc}}(^{17}\text{O})$ (ppm) | $\delta_{\text{iso, exp}}(^{17}\text{O})$ (ppm) |
|---|---|---|---|---|
| **I** | $H_2O$ | 332 | -13 | 0 |
| **II** | $CH_3OH$ | 329 | -9 | -37[34] |
| **III** | $CH_3CH_2OH$ | 297 | 20 | 6[34] |
| **IV** | ($t$Bu)OH | 234 | 77 | 62[34] |
| **V** | $(CH_3)_2CO$ | -342 | 603 | 625[33] |
| **VI** | $H_2O_2$ | 113 | 187 | 195[33] |
| **VII** | ($t$Bu)OOH (O) | 19 | 273 | 246[35] |
| | ($t$Bu)OOH (OH) | 103 | 197 | 206[35] |
| **VIII** | DMDO | 8 | 283 | 302[36] |
| **IX** | $m$CPBA (O) | 6 | 285 | 320[37] |
| | $m$CPBA (OH) | 25 | 268 | 275[37] |
| **X** | methyltrioxorhenium (MTO) | -613 | 850 | 820[33] |

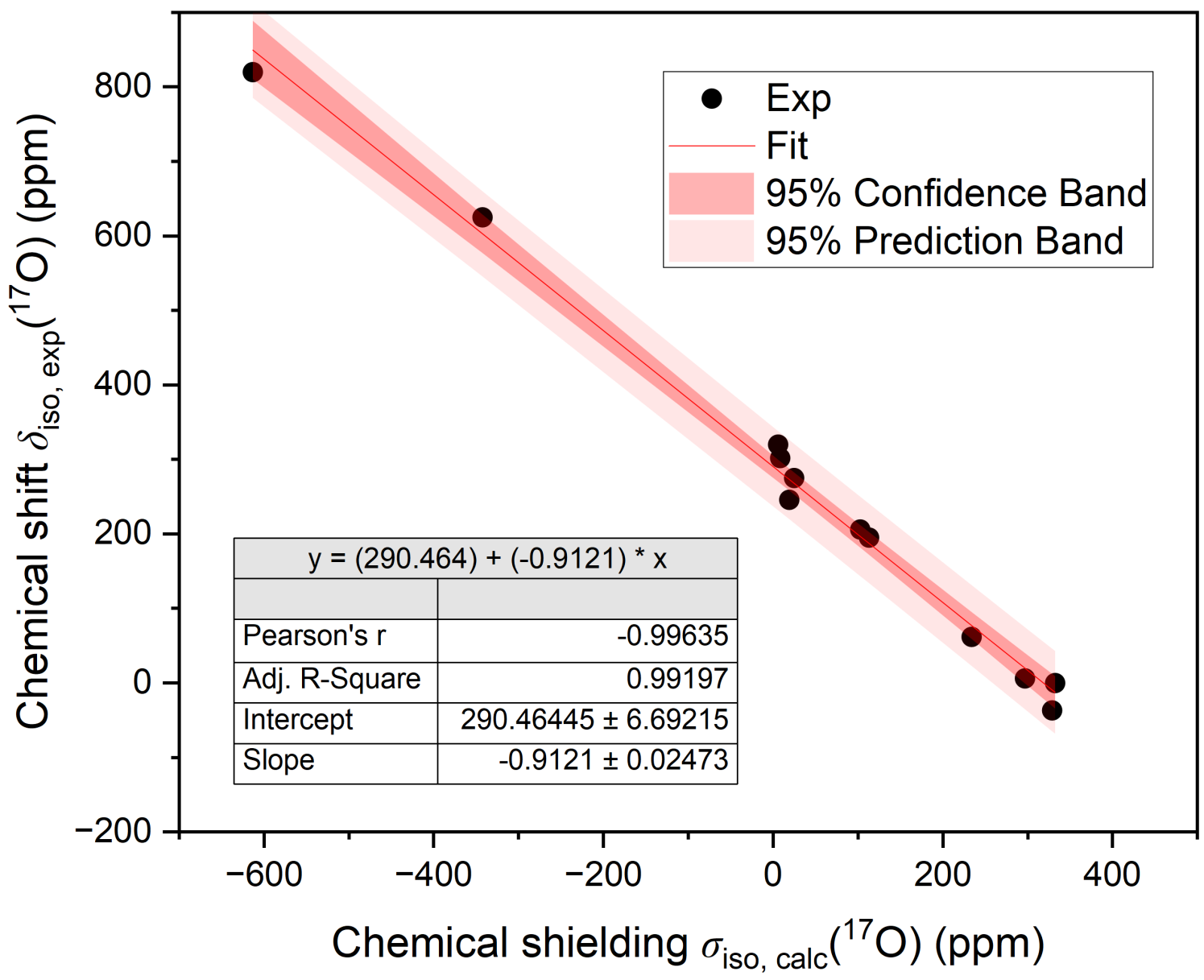


**Figure S36:** Correlation between the calculated isotropic shieldings $\sigma_{\text{iso, calc}}(^{17}\text{O})$ and the respective experimental isotropic chemical shifts $\delta_{\text{iso, exp}}(^{17}\text{O})$ reported in literature.[33–37]

## SG.2.2 $^{17}$O Chemical Shift Anisotropy

The principal components of the $^{17}$O NMR chemical shift tensors of the computed structures $j$ ($\delta_{ii,\text{ calc},j}(^{17}\text{O})$) were obtained from the calculated principal components of the $^{17}$O NMR shielding tensors ($\sigma_{ii,\text{ calc},j}(^{17}\text{O})$) via the correlation established for the isotropic values from the benchmark set (Equation SG.1). The calculated principal components of the $^{17}$O NMR chemical shift tensors of the benchmark structures (**V**, **VI** and **X**) yielded good agreement with the experimental principal components of the $^{17}$O NMR chemical shift tensors reported in literature.[33]

**Table S10:** Summary of computed and experimental principal components of the $^{17}$O NMR chemical shift tensors of the benchmark set. Calculated principal components of the shielding tensor $\sigma_{ii,\ \mathrm{calc}}(^{17}\mathrm{O})$ were obtained via the protocol described in Section SG.1.2 and used to calculate the principal components of the $^{17}$O NMR chemical shift tensors $\delta_{ii,\ \mathrm{calc}}(^{17}\mathrm{O})$ values via the correlation plotted in Figure S36.

| | Formula | $\sigma_{11,\ \mathrm{calc}}$ (ppm) | $\sigma_{22,\ \mathrm{calc}}$ (ppm) | $\sigma_{33,\ \mathrm{calc}}$ (ppm) | $\delta_{11,\ \mathrm{calc}}$ (ppm) | $\delta_{22,\ \mathrm{calc}}$ (ppm) | $\delta_{33,\ \mathrm{calc}}$ (ppm) | $\delta_{11,\ \mathrm{exp}}$ (ppm) | $\delta_{22,\ \mathrm{exp}}$ (ppm) | $\delta_{33,\ \mathrm{exp}}$ (ppm) |
|---|---|---|---|---|---|---|---|---|---|---|
| **V** | $(CH_3)_2CO$ | -919 | -418 | 309 | 1128 | 672 | 8 | 1185[33] | 705[33] | -15[33] |
| **VI** | $H_2O_2$ | -92 | 94 | 337 | 374 | 204 | -17 | 362[33] | 232[33] | -9[33] |
| **X** | MTO | -1128 | -429 | -283 | 1319 | 682 | 548 | 1326[33] | 629[33] | 506[33] |

### SG.2.3 $^{17}$O Quadrupole Coupling Constant

For the evaluation of the $^{17}$O quadrupole coupling constant calculations, computed values ($C_{\mathrm{Q,\ calc}}$) were compared with the experimental values ($C_{\mathrm{Q,\ exp}}$), reported in literature.[33] Overall, the calculations showed good agreement with the experimental values.

**Table S11:** Summary of computed and experimental $^{17}$O NMR quadrupole coupling constants of the benchmark set.

| | Formula | $C_{\mathrm{Q,\ calc}}$ (MHz) | $C_{\mathrm{Q,\ exp}}$ (MHz) |
|---|---|---|---|
| **V** | $(CH_3)_2CO$ | 11.9 | 12.0[33] |
| **VI** | $H_2O_2$ | 17.1 | 16.0[33] |
| **X** | MTO | 4.5 | 4.0[33] |

## SG.3 Models for Peroxo Formation on Ti Sites in Ti-MWW

The constructed models were geometry optimized as described in Section SG.1.1 followed by the same $^{17}$O NMR calculation protocol as described in Section SG.1.2. The considered peroxo models formed on Ti sites in Ti-MWW are displayed in Figure S37 and their respective $^{17}$O NMR parameters are given in Table S12. These parameters were used to simulate the $^{17}$O NMR lineshapes depicted in Figure 1b of the main text.

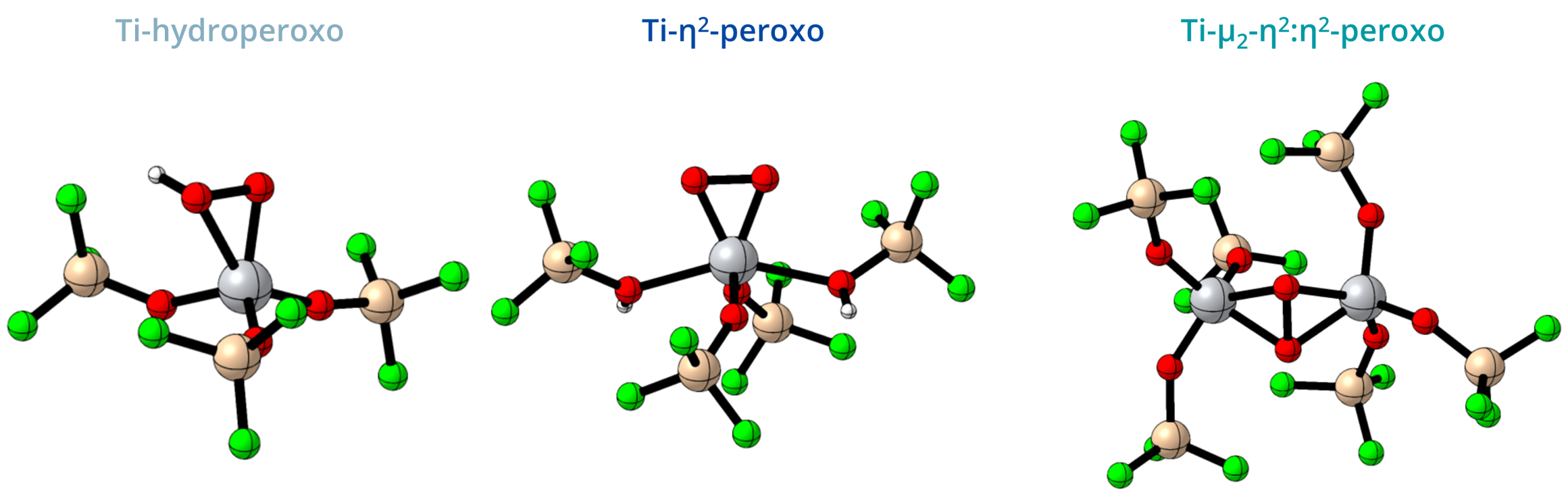


**Figure S37:** Models for the peroxo formation on Ti sites in Ti-MWW. (left) Ti-hydroperoxo model, (middle) Ti $\eta^2$-peroxo formed on mononuclear Ti sites, (right) Ti-$\mu_2$-$\eta^2$:$\eta^2$-peroxo formed on dinuclear Ti sites.

**Table S12:** Summary of $^{17}$O NMR parameters computed for the respective Ti-peroxo models. The Euler angles were taken from the corresponding fit of the experimental spectrum see Table S2.

| Model | Species | $\delta_{\mathrm{iso}}$ (ppm) | $\Omega$ (ppm) | $\kappa$ (ppm) | $C_\mathrm{Q}$ (MHz) | $\eta_\mathrm{Q}$ | $\varphi$ | $\chi$ | $\psi$ |
|---|---|---|---|---|---|---|---|---|---|
| **Ti-hydroperoxo** | O | 598 | 346 | -0.41 | 19.2 | 0.21 | 90 | 0 | 0 |
| | OH | 152 | 210 | 0.22 | 14.3 | 0.74 | 90 | 0 | 0 |
| **Ti-$\eta^2$-peroxo** | | 692 | 818 | -0.38 | 15.7 | 0.38 | 90 | 0 | 0 |
| **Ti-$\mu_2$-$\eta^2$:$\eta^2$-peroxo** | | 385 | 62 | -0.32 | 16.7 | 0.28 | 90 | 0 | 0 |

## SG.4 Models for Peroxo Formation on $ZnTiO_3$ and ZnO

The peroxo model constructed for the peroxo formation on $ZnTiO_3$ used a similar approach as described in Section SG.3, only deviating in the selected termination (see Figure S38). For the peroxo formed on ZnO the crystal structure of $ZnO_2$ (ICSD-38979,[38] ICSD release 2026)[39] was selected as an initial structure and cut to a cluster of the form $Zn_{13}O_{26}$ (see Figure S38). The cluster was allowed to optimized with all atoms being frozen expect the central two oxygen atoms for which the $^{17}$O NMR parameters have been calculated. The respective $^{17}$O NMR parameters of the calculated model structures are given in Table S13. These parameters were used to simulate the $^{17}$O NMR lineshapes depicted in Figure 1d of the main text.

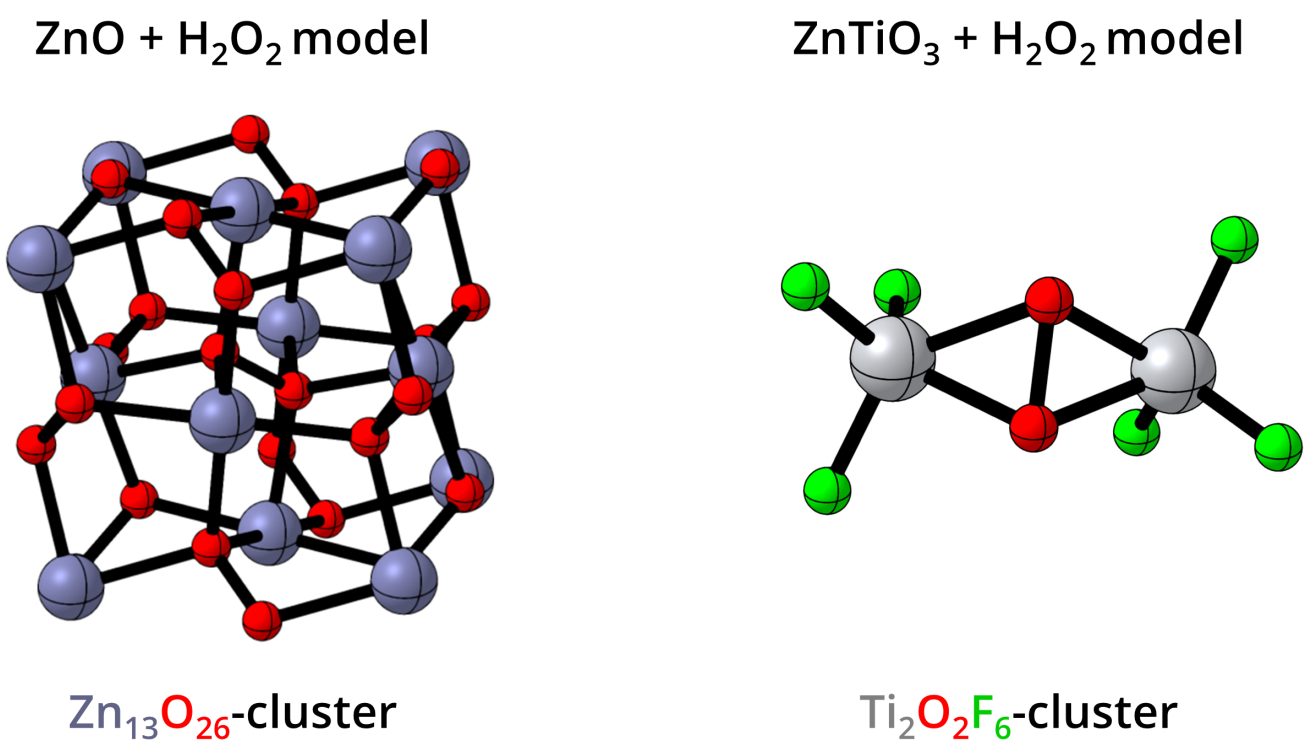


**Figure S38:** Models for the peroxo formation on $ZnTiO_3$ and ZnO. (left) *trans*-peroxo on ZnO, and (right) Ti-$\mu_2$-$\eta^2$:$\eta^2$-peroxo on $ZnTiO_3$.

**Table S13:** Summary of $^{17}$O NMR parameters computed for the respective Ti-peroxo models. The Euler angles were taken from the corresponding fit of the experimental spectrum see Table S3.

| Model | $\delta_{iso}$ (ppm) | $\Omega$ (ppm) | $\kappa$ (ppm) | $C_Q$ (MHz) | $\eta_Q$ | $\varphi$ | $\chi$ | $\psi$ |
|---|---|---|---|---|---|---|---|---|
| **Zn trans-peroxo** | 144 | 299 | 0.93 | 17.3 | 0.04 | 0 | 0 | 0 |
| **Ti-$\mu_2$-$\eta^2$:$\eta^2$-peroxo** | 404 | 102 | -0.63 | 16.9 | 0.31 | 90 | 0 | 0 |

## SH Additional Software Packages

In general graphics were made employing Python (version 3.12.13)[40] with the libraries Numpy (version 2.4.4)[41] and Matplotlib (version 3.10.9).[42] Additionally Nmrglue (version 0.11)[43] and TopSpin (version 3.6.5, Bruker BioSpin GmbH) were used to process NMR data. The OriginPro (version 2023)[44] software package was used for linear regressions. Molecular and surface structures were visualized using CYLView20[45] and Pymol (version 2.5.5)[46] respectively. For visualization purposes the MWW framework structure from the Database of Zeolite Structures[47] was used.

## References


(1) Parvulescu, A.-N.; Müller, U.; Teles, J. H.; Seelig, B.; Kampe, P.; Weber, M.; Bayer, R.; Seidel, K.; Resch, P. US20130204018 A1**, 2013**.

(2) Lätsch, L.; Müller, I. B.; Kaul, C. J.; Baerdemaeker, T. D.; Parvulescu, A.-N.; Seidel, K.; Teles, J. H.; Trukhan, N.; Copéret, C. Formation and Stability of µ2-Peroxo on Titanosilicates, Anatase, and Rutile: Implications for Zeotype Catalysts. *The Journal of Physical Chemistry C* **2024**, *128* (13), 5553–5558, DOI: 10.1021/acs.jpcc.3c08292.

(3) Parvulescu, A.-N.; Müller, U.; Lützel, H.-J.; Uhl, G.; Teles, J. H.; Riedel, D.; Urbanczyk, D.; Wegerle, U.; Weber, M.; Wörz, N. T.; Müller, C. US20200070134 A1**, 2017**.

(4) Rochlitz, L.; Searles, K.; Alfke, J.; Zemlyanov, D.; Safonova, O. V.; Copéret, C. Silica-supported, narrowly distributed, subnanometric Pt–Zn particles from single sites with high propane dehydrogenation performance. *Chemical Science* **2020**, *11* (6), 1549–1555, DOI: 10.1039/C9SC05599A.

(5) George, S. D. B.; Brant, P.; Solomon, E. I. Metal and ligand K-edge XAS of organotitanium complexes: Metal 4p and 3d contributions to pre-edge intensity and their contributions to bonding. *Journal of the American Chemical Society* **2005**, *127* (2), DOI: 10.1021/ja044827v.

(6) Piamonteze, C.; Flechsig, U.; Rusponi, S.; Dreiser, J.; Heidler, J.; Schmidt, M.; Wetter, R.; Calvi, M.; Schmidt, T.; Pruchova, H.; Krempasky, J.; Quitmann, C.; Brune, H.; Nolting, F. X-Treme beamline at SLS: X-ray magnetic circular and linear dichroism at high field and low temperature. *Journal of Synchrotron Radiation* **2012**, *19* (5), 661–674, DOI: 10.1107/S0909049512027847.

(7) Van der Laan, G.; Figueroa, A. I. X-ray magnetic circular dichroism—A versatile tool to study magnetism. *Coordination Chemistry Reviews* **2014**, *277-278*, 95–129, DOI: 10.1016/j.ccr.2014.03.018.

(8) Vásquez, G. C.; Maestre, D.; Cremades, A.; Ramírez-Castellanos, J.; Magnano, E.; Nappini, S.; Karazhanov, S. Z. Understanding the effects of Cr doping in rutile TiO2 by DFT calculations and X-ray spectroscopy. *Scientific Reports* **2018**, *8* (1), 8740, DOI: 10.1038/s41598-018-26728-3.

(9) Yamamoto, K.; Tanaka, S.; Hosoya, H.; Tsurugi, H.; Mashima, K.; Copéret, C. Activation of O2 by Organosilicon Reagents Yields Quantitative Amounts of H2O2 or (Me3Si)2O2 for Efficient O-Transfer Reactions. *Helvetica Chimica Acta* **2018**, *101* (12), DOI: 10.1002/hlca.201800156.

(10) O'Dell, L. A.; Schurko, R. W. QCPMG using adiabatic pulses for faster acquisition of ultra-wideline NMR spectra. *Chemical Physics Letters* **2008**, *464* (1-3), 97–102, DOI: 10.1016/j.cplett.2008.08.095.

(11) Schurko, R. W. Ultra-Wideline Solid-State NMR Spectroscopy. *Accounts of Chemical Research* **2013**, *46* (9), 1985–1995, DOI: 10.1021/ar400045t.

(12) Keeler, E. G.; Michaelis, V. K.; Wilson, C. B.; Hung, I.; Wang, X.; Gan, Z.; Griffin, R. G. High-Resolution 17O NMR Spectroscopy of Structural Water. *The Journal of Physical Chemistry B* **2019**, *123* (14), 3061–3067, DOI: 10.1021/acs.jpcb.9b02277.

(13) Massiot, D.; Fayon, F.; Capron, M.; King, I.; Calvé, S. L.; Alonso, B.; Durand, J.; Bujoli, B.; Gan, Z.; Hoatson, G. Modelling one- and two-dimensional solid-state NMR spectra. *Magnetic Resonance in Chemistry* **2002**, *40* (1), 70–76, DOI: 10.1002/mrc.984.

(14) Bertani, P.; Raya, J.; Bechinger, B. 15N chemical shift referencing in solid state NMR. *Solid State Nuclear Magnetic Resonance* **2014**, *61-62*, 15–18, DOI: 10.1016/j.ssnmr.2014.03.003.

(15) Grimme, S.; Hansen, A.; Ehlert, S.; Mewes, J.-M. r2SCAN-3c: A "Swiss army knife" composite electronic-structure method. *The Journal of Chemical Physics* **2021**, *154* (6), DOI: 10.1063/5.0040021.

(16) Neese, F.; Wennmohs, F.; Becker, U.; Riplinger, C. The ORCA quantum chemistry program package. *The Journal of Chemical Physics* **2020**, *152* (22), DOI: 10.1063/5.0004608.

(17) Neese, F. Software Update: The ORCA Program System—Version 6.0. *WIREs Computational Molecular Science* **2025**, *15* (2), DOI: 10.1002/wcms.70019.

(18) Furness, J. W.; Kaplan, A. D.; Ning, J.; Perdew, J. P.; Sun, J. Accurate and Numerically Efficient r2SCAN Meta-Generalized Gradient Approximation. *The Journal of Physical Chemistry Letters* **2020**, *11* (19), 8208–8215, DOI: 10.1021/acs.jpclett.0c02405.

(19) Ehlert, S.; Huniar, U.; Ning, J.; Furness, J. W.; Sun, J.; Kaplan, A. D.; Perdew, J. P.; Brandenburg, J. G. r2SCAN-D4: Dispersion corrected meta-generalized gradient approximation for general chemical applications. *The Journal of Chemical Physics* **2021**, *154* (6), DOI: 10.1063/5.0041008.

(20) Kruse, H.; Grimme, S. A geometrical correction for the inter- and intra-molecular basis set superposition error in Hartree-Fock and density functional theory calculations for large systems. *The Journal of Chemical Physics* **2012**, *136* (15), DOI: 10.1063/1.3700154.

(21) Schreckenbach, G.; Ziegler, T. Calculation of NMR Shielding Tensors Using Gauge-Including Atomic Orbitals and Modern Density Functional Theory. *The Journal of Physical Chemistry* **1995**, *99* (2), 606–611, DOI: 10.1021/j100002a024.

(22) Krykunov, M.; Ziegler, T.; van Lenthe, E. Hybrid density functional calculations of nuclear magnetic shieldings using Slater-type orbitals and the zeroth-order regular approximation. *International Journal of Quantum Chemistry* **2009**, *109* (8), 1676–1683, DOI: 10.1002/qua.21985.

(23) Van Lenthe, E.; Baerends, E. J. Density functional calculations of nuclear quadrupole coupling constants in the zero-order regular approximation for relativistic effects. *The Journal of Chemical Physics* **2000**, *112* (19), 8279–8292, DOI: 10.1063/1.481433.

(24) Van Lenthe, E.; Baerends, E. J.; Snijders, J. G. Relativistic regular two-component Hamiltonians. *The Journal of Chemical Physics* **1993**, *99* (6), 4597–4610, DOI: 10.1063/1.466059.

(25) Van Lenthe, E.; Baerends, E. J.; Snijders, J. G. Relativistic total energy using regular approximations. *The Journal of Chemical Physics* **1994**, *101* (11), 9783–9792, DOI: 10.1063/1.467943.

(26) Van Lenthe, E.; Ehlers, A.; Baerends, E.-J. Geometry optimizations in the zero order regular approximation for relativistic effects. *The Journal of Chemical Physics* **1999**, *110* (18), 8943–8953, DOI: 10.1063/1.478813.

(27) Wolff, S. K.; Ziegler, T.; van Lenthe, E.; Baerends, E. J. Density functional calculations of nuclear magnetic shieldings using the zeroth-order regular approximation (ZORA) for relativistic effects: ZORA nuclear magnetic resonance. *The Journal of Chemical Physics* **1999**, *110* (16), 7689–7698, DOI: 10.1063/1.478680.

(28) Adamo, C.; Barone, V. Toward reliable density functional methods without adjustable parameters: The PBE0 model. *The Journal of Chemical Physics* **1999**, *110* (13), 6158–6170, DOI: 10.1063/1.478522.

(29) Lenthe, E. V.; Baerends, E. J. Optimized Slater-type basis sets for the elements 1–118. *Journal of Computational Chemistry* **2003**, *24* (9), 1142–1156, DOI: 10.1002/jcc.10255.

(30) Te Velde, G.; Bickelhaupt, F. M.; Baerends, E. J.; Guerra, C. F.; van Gisbergen, S. J. A.; Snijders, J. G.; Ziegler, T. Chemistry with ADF. *Journal of Computational Chemistry* **2001**, *22* (9), 931–967, DOI: 10.1002/jcc.1056.

(31) ADF 2023.101, SCM, Theoretical Chemistry, Vrije Universiteit, Amsterdam, The Netherlands**, 2023**, http://www.scm.com.

(32) AMS, SCM, Theoretical Chemistry, Vrije Universiteit, Amsterdam, The Netherlands**, 2023**, http://www.scm.com.

(33) Ehinger, C.; Gordon, C. P.; Copéret, C. Oxygen transfer in electrophilic epoxidation probed by 17O NMR: differentiating between oxidants and role of spectator metal oxo. *Chemical Science* **2019**, *10* (6), 1786–1795, DOI: 10.1039/C8SC04868A.

(34) Crandall, J. K.; Centeno, M. A. Oxygen-17 nuclear magnetic resonance. 1. Alcohols. *The Journal of Organic Chemistry* **1979**, *44* (7), 1183–1184, DOI: 10.1021/jo01321a042.

(35) Barieux, J.; Schirmann, J. 17O-enriched hydrogen peroxide and t.butyl hydroperoxide: Synthesis, characterization and some applications. *Tetrahedron Letters* **1987**, *28* (51), 6443–6446, DOI: 10.1016/S0040-4039(00)96883-7.

(36) Cassidei, L.; Fiorentino, M.; Mello, R.; Sciacovelli, O.; Curci, R. Oxygen-17 and carbon-13 identification of the dimethyldioxirane intermediate arising in the reaction of potassium caroate with acetone. *The Journal of Organic Chemistry* **1987**, *52* (4), 699–700, DOI: 10.1021/jo00380a045.

(37) Antolini, L.; Benassi, R.; Ghelli, S.; Folli, U.; Sbardellati, S.; Taddei, F. The influence of steric constraints on the conformational properties and on the 17O NMR shielding of ortho-substituted perbenzoates. *Journal of the Chemical Society, Perkin Transactions 2* **1992**, (11), 1907, DOI: 10.1039/p29920001907.

(38) Bocharov, D.; Chesnokov, A.; Chikvaidze, G.; Gabrusenoks, J.; Ignatans, R.; Kalendarev, R.; Krack, M.; Kundzins, K.; Kuzmin, A.; Mironova-Ulmane, N.; Pudza, I.; Puust, L.; Sildos, I.; Vasil'chenko, E.; Zubkins, M.; Purans, J. A comprehensive study of structure and properties of nanocrystalline zinc peroxide. *Journal of Physics and Chemistry of Solids* **2022**, *160*, 110318, DOI: 10.1016/j.jpcs.2021.110318.

(39) Zagorac, D.; Müller, H.; Ruehl, S.; Zagorac, J.; Rehme, S. Recent developments in the Inorganic Crystal Structure Database: theoretical crystal structure data and related features. *Journal of Applied Crystallography* **2019**, *52* (5), 918–925, DOI: 10.1107/S160057671900997X.

(40) Python, 3.12.13, **2025**, https://www.python.org.

(41) Harris, C. R.; Millman, K. J.; van der Walt, S. J.; Gommers, R.; Virtanen, P.; Cournapeau, D.; Wieser, E.; Taylor, J.; Berg, S.; Smith, N. J.; Kern, R.; Picus, M.; Hoyer, S.; van Kerkwijk, M. H.; Brett, M.; Haldane, A.; del Río, J. F.; Wiebe, M.; Peterson, P.; Gérard-Marchant, P.; Sheppard, K.; Reddy, T.; Weckesser, W.; Abbasi, H.; Gohlke, C.; Oliphant, T. E. Array programming with NumPy, **2020**, DOI: 10.1038/s41586-020-2649-2.

(42) Hunter, J. D. Matplotlib: A 2D graphics environment. *Computing in Science and Engineering* **2007**, *9* (3), DOI: 10.1109/MCSE.2007.55.

(43) Helmus, J. J.; Jaroniec, C. P. Nmrglue: An open source Python package for the analysis of multidimensional NMR data. *Journal of Biomolecular NMR* **2013**, *55* (4), DOI: 10.1007/s10858-013-9718-x.

(44) OriginPro, 2023, OriginLab Corporation, Northampton, MA, USA.

(45) Legault, C. Y. CYLview20, Université de Sherbrooke, **2020**, http://www.cylview.org.

(46) The PyMOL Molecular Graphics System, Schrödinger, LLC.

(47) Baerlocher, C.; Brouwer, D.; Marler, B.; McCusker, L. B. Database of Zeolite Structures, **2025**, https://www.iza-structure.org/databases/.
